# Efficacy and Safety of a Krabbe Disease Gene Therapy

**DOI:** 10.1089/hum.2021.245

**Published:** 2022-05-16

**Authors:** Juliette Hordeaux, Brianne A. Jeffrey, Jinlong Jian, Gourav R. Choudhury, Kristofer Michalson, Thomas W. Mitchell, Elizabeth L. Buza, Jessica Chichester, Cecilia Dyer, Jessica Bagel, Charles H. Vite, Allison M. Bradbury, James M. Wilson

**Affiliations:** ^1^ Gene Therapy Program, Department of Medicine, Perelman School of Medicine, University of Pennsylvania, Philadelphia, Pennsylvania, USA; ^2^ Department of Clinical Sciences and Advanced Medicine, University of Pennsylvania, Philadelphia, Pennsylvania, USA.

**Keywords:** AAV, Krabbe, cisterna magna, galactosylceramidase (GALC), psychosine, galactosylceramide, lysosomal storage disease, Twitcher mouse, demyelination

## Abstract

Krabbe disease is a lysosomal storage disease caused by mutations in the gene that encodes galactosylceramidase, in which galactosylsphingosine (psychosine) accumulation drives demyelination in the central and peripheral nervous systems, ultimately progressing to death in early childhood. Gene therapy, alone or in combination with transplant, has been developed for almost two decades in mouse models, with increasing therapeutic benefit paralleling the improvement of next-generation adeno-associated virus (AAV) vectors. This effort has recently shown remarkable efficacy in the canine model of the disease by two different groups that used either systemic or cerebrospinal fluid (CSF) administration of AAVrh10 or AAV9. Building on our experience developing CSF-delivered, AAV-based drug products for a variety of neurodegenerative disorders, we conducted efficacy, pharmacology, and safety studies of AAVhu68 delivered to the CSF in two relevant natural Krabbe animal models, and in nonhuman primates. In newborn Twitcher mice, the highest dose (1 × 10^11^ genome copies [GC]) of AAVhu68.hGALC injected into the lateral ventricle led to a median survival of 130 days compared to 40.5 days in vehicle-treated mice. When this dose was administered intravenously, the median survival was 49 days. A single intracisterna magna injection of AAVhu68.cGALC at 3 × 10^13^ GC into presymptomatic Krabbe dogs increased survival for up to 85 weeks compared to 12 weeks in controls. It prevented psychosine accumulation in the CSF, preserved peripheral nerve myelination, ambulation, and decreased brain neuroinflammation and demyelination, although some regions remained abnormal. In a Good Laboratory Practice-compliant toxicology study, we administered the clinical candidate into the cisterna magna of 18 juvenile rhesus macaques at 3 doses that displayed efficacy in mice. We observed no dose-limiting toxicity and sporadic minimal degeneration of dorsal root ganglia (DRG) neurons. Our studies demonstrate the efficacy, scalability, and safety of a single cisterna magna AAVhu68 administration to treat Krabbe disease. ClinicalTrials.Gov ID: NCT04771416.

## INTRODUCTION

Krabbe disease (globoid cell leukodystrophy) is an autosomal recessive lysosomal storage disease caused by mutations in the gene encoding the hydrolytic enzyme galactosylceramidase (GALC).^1^ This enzyme is responsible for the degradation of galactosylceramide (GalCer; a type of ceramide) and galactosylsphingosine (psychosine). GALC deficiency causes toxic accumulation of psychosine in the plasma membrane of cells throughout the nervous system.^2,3^ The myelin-producing oligodendrocytes in the central nervous system (CNS) and Schwann cells in the peripheral nervous system (PNS) are sensitive to psychosine accumulation, which can cause death of these cell populations.^4^

However, recent work suggests that other cell-autonomous defects in neurons and macrophages also contribute to the pathophysiology of Krabbe disease.^5–8^ The breakdown of myelin in the CNS and PNS is accompanied by reactive astrocytic gliosis, microgliosis, and the infiltration of macrophages that cannot digest the myelin debris due to their enzymatic deficit. The phagocytic cells adopt a giant multinucleated morphology (“globoid cells”) that is characteristic of the progressive severe neuroinflammation associated with Krabbe disease.^8–10^

Clinically, Krabbe disease is divided into four forms, depending on age at symptom-onset: early infantile; late infantile; juvenile; and adolescent or adult Krabbe disease. The most severe early infantile form manifests before 6 months of age and is fatal by 2 years of age in most patients.^11–13^ Demyelination and globoid cell infiltration affects both the peripheral nerves and CNS. Disease progression in peripheral nerves can be monitored via nerve conduction velocity testing, while CNS white matter demyelination can be visualized on magnetic resonance imaging (MRI),^14^ and manifest as abnormal brainstem auditory evoked responses.^11,15,16^

The only disease-modifying treatment—hematopoietic stem cell transplantation (HSCT)—shows significant survival benefit when performed in presymptomatic newborns.^17^ However, the therapeutic window is narrow due to the competition between rapid disease progression and slow cell engraftment in the CNS. Transplant is only efficacious when performed within the first month of life; even then, motor outcomes are poor and peripheral neuropathy remains unaffected.^13,17–19^

Development of experimental treatments for Krabbe disease benefits from the availability of multiple relevant animal models from mouse and dog to nonhuman primates.^20–22^ Early work using recombinant enzyme replacement therapy administered weekly into the peritoneal cavity or once into the lateral ventricle of Twitcher mice demonstrated a modest survival benefit and reduction of psychosine levels, paving the way for other therapies that rely on cross correction.^23,24^

Rafi *et al.* demonstrated that direct administration of AAV1.mGALC into the cerebral ventricles of neonatal mice achieved neuronal transduction, cross-correction of glial cells, prevention of psychosine accumulation, and mild extension of the lifespan from 42 to up to 66 days.^25^ Lin *et al.* demonstrated the synergy of intracerebral AAV5 combined with bone marrow transplant (BMT) to extend survival beyond 150 days.^26^

The following decade saw multiple teams push the limit of disease prevention in the mouse model through the use of next-generation capsids, dose optimization, and/or combination therapies.

Using AAVrh10, Rafi *et al.* achieved greater efficacy in mice by either increasing the dose via combined route of administration,^27^ combination of systemic AAVrh10 with BMT,^28^ or more recently using systemic AAVrh10 at a very high dose, the latter of which led to a maximal survival of 430 days (median 280 days).^29^ Karumuthil-Melethil *et al.* compared AAV9, AAVrh10, or AAVolig001 via lumbar administration with or without BMT, and found the best outcomes with an ssAAV9 construct when combined with BMT.^30^ Both groups translated their studies to the clinically relevant dog model and obtained remarkable dose-dependent disease mitigation with AAVrh10^31^ or AAV9.^32^

Finally, combination therapy using intracerebroventricular (ICV) AAV9, substrate reduction, and BMT in newborn Twitcher mice achieved complete disease prevention, although long-term survivors developed hepatocellular carcinoma.^33^

We and others have demonstrated the remarkable translational efficacy of cerebrospinal fluid (CSF)-delivered adeno-associated virus (AAV) as a monotherapy for the treatment of neuropathic lysosomal storage disorders in large animal models.^32,34–41^ Robust transduction of lower motor neurons in the spinal cord and dorsal root ganglia (DRG) neurons, as well as scattered transduction of cortical neurons, cerebellar Purkinje cells, and medulla motor neurons, can provide a broad source of replacement enzyme that can support cross-correction of peripheral nerves and CNS cells.^36,38,42–45^

Compared to systemic administration of neurotropic vectors at high doses, the CSF-directed approach appears to be safer.^46–52^ This approach also consumes less vector and is not limited by preexisting neutralizing antibodies (NAbs).^42,50^ Good Laboratory Practice (GLP)-compliant studies in nonhuman primates demonstrated the safety of the intracisterna magna (ICM) AAV-mediated approach with several drug candidates that are being evaluated in clinical trials.^50,51^

We performed nonclinical studies supporting an investigational new drug (IND) application for the treatment of infantile Krabbe disease using an AAVhu68 vector expressing a codon-optimized *GALC* administered into the cisterna magna (NCT04771416). In this study, we report the findings from three preclinical studies. The first entailed route-of-administration (ROA) and dose-finding studies in neonatal and juvenile Twitcher mice using the ICV or intravenous (IV) routes. The second is a pilot efficacy study in Krabbe dogs using the intended clinical route (*i.e*., ICM). The third is a GLP-compliant toxicology study in juvenile rhesus macaques using clinically comparable fluoroscopy-guided ICM administration.

Our results demonstrate a translationally feasible and efficacious approach to treat Krabbe disease using a single ICM administration of AAVhu68.hGALC without concomitant transplant or immune suppression.

## MATERIALS AND METHODS

### Data availability statement

All data discussed in the article are available in the main text or Supplementary Data. Complete clinical pathology data are available in Supplementary Data S1 and S2.

### Animals

#### Mice

The Institutional Animal Care and Use Committee of the University of Pennsylvania approved all animal procedures in this study. We purchased the Twitcher mouse model, a spontaneous model identified in the C57BL6/J background at the Jackson Laboratory in 1976,^20^ from the Jackson Laboratory (stock No. 000845). We housed the animals in an Association for Assessment and Accreditation of Laboratory Animal Care (AAALAC) International-accredited mouse barrier vivarium at the Gene Therapy Program, University of Pennsylvania, in microisolator cages.

We provided enrichment (Nestlets nesting material). Cages, water bottles, and bedding substrates were autoclaved in the barrier facility, and cages were changed once per week. We maintained an automatically controlled 12-h light/dark cycle. Irradiated laboratory rodent food was provided *ad libitum*, and nutritional support (DietGel^®^ ClearH_2_0) was provided to all mice after weaning until the end of the study.

The mice were monitored twice daily and weighed three times per week. We defined the humane endpoint as a body weight loss exceeding 20% from the peak body weight or severe dragging of the hindlegs. The animals were randomly assigned to a treatment group, and the staff were blinded to the genotype and treatment group of the animals.

#### Dogs

We accessed the Krabbe dog, a naturally occurring model derived from a spontaneous missense A-to-C mutation in the *GALC* gene, through the Referral Center for Animal Models of Human Genetic Disease of the School of Veterinary Medicine of the University of Pennsylvania (NIH OD P40-10939). Two- to three-week-old (body weight: 0.270–0.350 kg) dogs were enrolled (*n* = 7; 6 homozygous for the *GALC* Y158C mutation and 1 wild type, WT).

Animals received humane care in accordance with the “Guide for the Care and Use of Laboratory Animals” (NIH). The Testing Facility at PennVet conducted the study in accordance with the Animal and Plant Health Inspection Service, United States Department of Agriculture (USDA), and the Animal Welfare Act, 9 CFR, Parts 1, 2, and 3 as applicable. The animal facility is AAALAC-accredited and USDA-registered.

Animals were housed in stainless steel cages with the mother (dam) and littermates until weaning. We pair-housed the animals with littermates after weaning. We fed the canine puppies IAMS commercial dry food mixed with Purina adult canned food until they reached 4 months of age. IAMS commercial dry food was available *ad libitum* until the dogs were 1 year of age. After 1 year of age, the animals were switched to Purina adult dry food and given recommended amounts for a healthy body weight. Water was provided *ad libitum*. We provided environmental enrichment to the animals, including daily socialization via interactions with staff. The dogs had access to toys at all times.

#### Nonhuman primates

We procured juvenile rhesus macaques (*Macaca mulatta*) from Primgen/Prelabs Primates. The macaques were 15–19 months of age at study initiation (*n* = 2 vehicle-treated, *n* = 18 vector-treated). We housed the animals in the AAALAC International-accredited Nonhuman Primate Research Program facility at the University of Pennsylvania in stainless steel squeeze-back cages as groups. Animals received varied enrichments such as food treats, visual and auditory stimuli, manipulatives, and social interactions.

### Vectors

The Penn Vector Core produced and titrated AAV vectors for the mouse and dog studies as previously described.^53^ In brief, HEK293 cells were triple-transfected, and the culture supernatant was harvested, concentrated, and purified with an iodixanol gradient. For the GLP-compliant toxicology study, the test article was manufactured under conditions as similar as possible to good manufacturing practice guidelines.

The vector was produced by triple transfection of adherent HEK293 cells and purified from supernatant by affinity chromatography using a POROS^™^ CaptureSelect^™^ AAV9 resin (Thermo Fisher Scientific, Waltham, MA), followed by anion exchange chromatography. Limulus amebocyte lysate and quantitative polymerase chain reaction (qPCR) tests for endotoxin and mycoplasma, respectively, were negative. Vector titer by TaqMan PCR was 5.67 × 10^13^ genome copies (GC)/mL.

The purity of capsid proteins was 95.4%, as determined by sodium dodecyl sulfate-polyacrylamide gel electrophoresis analysis. Analytical ultracentrifugation indicated that the preparation contained 68% full-vector particles. We confirmed the *in vitro* potency of the vector, assessed by GALC enzyme activity, as similar to that of reference vector lots. We titrated the purified vectors with droplet digital PCR using primers targeting the rabbit beta-globin polyA sequence as previously described.^54^

### *In vivo* studies

#### Test article and vehicle administration

Mice underwent an ICV administration procedure in accordance with standard operating procedures by a trained laboratory animal veterinarian who is board-certified by the American College of Laboratory Animal Medicine or a veterinarian who was trained and achieved a success rate of at least 95%.

In brief, neonatal mice were cryoanesthetized, and juvenile mice were anesthetized with isoflurane. We cleaned the scalp with 70% ethanol and connected a 10 μL syringe to a 30-gauge needle with an attached plastic stopper that left only 3 mm of the needle tip exposed. We inserted the needle into the lateral ventricle and administered a total volume of 2 μL (newborns) or 4 μL (juveniles) of either the test article diluted to the appropriate concentration or vehicle (1 × Dulbecco's phosphate-buffered saline in newborns and artificial CSF in juvenile mice).

Dogs received vector or vehicle (artificial CSF) via suboccipital puncture into the cisterna magna. An experienced veterinarian performed this procedure at Penn Vet. In brief, the dogs were sedated with IV propofol and placed on the procedure table in the lateral decubitus position with the head flexed forward for CSF collection and dosing into the cisterna magna. The site of injection was aseptically prepared.

Based on an aseptic technique, a 22-gauge, 1.5″ spinal needle (Becton Dickinson) was advanced into the cerebellomedullary cistern until the flow of CSF was observed. We collected up to 1.0 mL of CSF for baseline analysis before dosing. After CSF collection, the syringe containing 1 mL of either test article or vehicle was connected to the spinal needle and slowly injected. The needle was removed, and direct pressure was applied to the puncture site.

Nonhuman primates (NHPs) received vectors or vehicle (artificial CSF) in a volume of 1 mL injected into the cisterna magna under fluoroscopic guidance, as previously described.^55^ We performed periodic blood collection and CSF taps for safety readouts. The contract facility Antech GLP and Antech Diagnostics performed serum chemistry, hematology, coagulation, and CSF analyses.

### *In vivo* monitoring of the phenotype

#### Mice

Personnel blinded to the treatment group weighed and monitored the mice at least three times a week via compound scoring to assess hind limb clasping ability, gait, tremors, spinal curvature (kyphosis), and fur quality (Supplementary Table S1). We chose these measures to assess clinical status based on the symptoms typically exhibited by Twitcher mice. Scores above 0 indicate clinical deterioration. We measured coordination and balance using the rotarod test (Ugo Basile, Gemonio, Italy).

In brief, we first habituated mice to the rotarod by placing up to five mice per trial in a lane of the rotarod device facing the wall. Mice were allowed to stabilize themselves on the fixed (nonrotating) rod for 2 min. We then performed two habituation trials with the rod rotating for 1 min at a constant speed of 5 revolutions per minute (RPM). Immediately following habituation, we performed testing trials to measure how long each mouse could remain on the rotating rod as it accelerated from 5 to 40 RPM over 120 s.

#### Dogs

Animals underwent standardized physical and neurological assessments that included evaluation of gait, ataxia, posture, tremors, reflexes, menace response, nystagmus, mental status, and muscle atrophy based on the scoring system presented in Supplementary Table S2 (for proprioception, ataxia, head tremor/truncal sway, muscle atrophy 0 = no deficit, 1 = mild deficit, 2 = moderate deficit, 3 = severe deficit; for spinal reflexes, 0 = no deficit, 1 = mild hyporeflexia; for lack of menace response, 0 = yes, 1 = no). We performed the tests for each assessment in the same order each time. A board-certified veterinary neurologist blinded to the animal's genotype and treatment performed the assessments. The onset and progression, as well as the severity of signs of neurologic dysfunction, were documented.

For electrophysiology, the animals were anesthetized with propofol, endotracheally intubated, and maintained on isoflurane-inhaled anesthesia. We conducted nerve conduction studies using an electrodiagnostics machine (Nicolet Viking Quest). We determined motor nerve conductions in the tibial, sciatic, and ulnar nerves of the left limb using a 12 mm 29-gauge subdermal needle recording electrode placed in the interosseous muscle. The tibial nerve received electrical stimulation at the tarsus. The sciatic nerve received electrical stimulation at the tibial nerve branch and at the level of the stifle. The ulnar nerve received stimulation at the carpus and elbow.

We stimulated nerves with monopolar stimulating electrodes placed subcutaneously for a duration of 200 μs. The stimulus intensity increased until an M wave of maximal amplitude was obtained. We determined sensory nerve conduction velocities for the radial nerve. Subcutaneous recording electrodes were placed lateral to the radial nerve at the level of the elbow and the skin over the dorsum of the paw and stimulated with subcutaneous monopolar stimulating electrodes for 200 μs.

We recorded brainstem auditory evoked response data on a Nicolet Viking Quest machine (Nicolet Biomedical, Madison, WI) using 12 mm 29-gauge subdermal needle recording electrodes. We placed the active electrode in the skin over the osseous bulla of the stimulated ear, the reference electrode in the skin over the vertex of the skull, and the ground electrode in the skin over the contralateral osseous bulla. We delivered alternating rarefaction and condensation clicks (0.1 ms in duration) to the stimulated ear at 11.1 Hz using a 25 cm plastic tube connected to a plastic earpiece placed within the external ear canal.

The filter settings for the amplifier were 20 Hz and 3 kHz. We averaged 1,000 evoked responses for each tracing obtained. An amplifier sensitivity of 1 μV/cm was used to record the responses, and the analysis time was 10 ms. We defined the central conduction time as the time between the first and fifth peaks and defined the hearing threshold as the sound intensity at which an evoked waveform was first visible.

For brain MRI, dogs were anesthetized with propofol, endotracheally intubated, and maintained on isoflurane-inhaled anesthesia. We performed imaging at 1.5 T using a dedicated research MRI scanner (Signa; GE Corporation, Milwaukee, WI). Imaging sessions consisted of T2-weighted sagittal spin-echo imaging, T1-weighted axial spin-echo imaging, axial T2-weighted fast spin-echo imaging; and T1-weighted dorsal plane axial conventional spin-echo imaging. Gadolinium was then administered, and short T1-weighted images were acquired.

#### Nonhuman primates

The animal care and/or veterinary staff visually monitored the NHPs daily for any conditions requiring possible intervention. This included monitoring the general appearance of the animal for signs of toxicity, distress, and/or changes in behavior. At select study time points, the animals were also monitored for additional parameters, including but not limited to, vital signs, and had blood collected for clinical pathology. All animals enrolled in the described study had a neurological assessment up to once per month. The neurological examination involved cage-side evaluation of mentation, posture, proprioception, and gait, as well as a restrained evaluation of cranial nerves, motor strength, and reflexes.

The care staff observed the animals daily for any signs of pain or discomfort, such as changes in behavior or significant changes in appetite. Any clinical abnormalities were reported to study veterinarians and the study director; none of the reported abnormalities were suspected to be related to test article administration.

For nerve conduction velocity testing, NHPs were sedated with a combination of ketamine/dexmedetomidine and placed in lateral or dorsal recumbency on a procedure table, with heat packs to maintain body temperature. We positioned the stimulator probe over the median nerve with the cathode closest to the recording site. We subcutaneously inserted two needle electrodes on digit II at the level of the distal phalanx (reference electrode) and proximal phalanx (recording electrode). Meanwhile, we placed the ground electrode proximal to the stimulating probe (cathode).

A pediatric stimulator delivered the stimulus, which we increased in a stepwise manner until the peak amplitude response was reached. Up to 10 supramaximal stimuli were averaged and reported for the median nerve. We measured the distance (cm) from the recording electrode to the stimulation cathode and used this distance to calculate the conduction velocity. We measured both the conduction velocity and the average of the sensory nerve action potential (SNAP) amplitude.

### Necropsy and histology

Mice were deeply anesthetized with isoflurane, and euthanized by exsanguination. Large animals were euthanized by IV pentobarbital overdose under deep sedation. We then harvested the tissues for comprehensive histopathologic examination. Collected tissues were immediately flash frozen for enzyme activity and biodistribution analysis or fixed in formalin for paraffin embedding.

For histopathology, we stained tissue sections with hematoxylin and eosin following standard protocols. We stained CNS and PNS tissues using Luxol fast blue (LFB)/periodic acid-Schiff (PAS). In brief, we incubated slides with LFB solution (Cat. No. STLFBPT; SLMP, LLC) overnight at 65°C. Sections were differentiated in a 0.05% lithium carbonate and 70% ethanol solution and monitored under the microscope until differentiation was completed. We then placed the slides in 0.5% periodic acid for 5 min (Cat. No. 395B-1Kit; Sigma).

After a washing step with running tap water, we transferred the slides to Schiff's reagent (Cat. No. 395B-1Kit; Sigma) for 15 min. Slides were washed with running tap water for 5 min and briefly counterstained with hematoxylin for nucleus identification, and coverslips were applied. A veterinary pathologist analyzed the slides in a blinded manner and established semiquantitative severity grades for demyelination (Luxol blue), globoid cell infiltrates (PAS), and other histopathological findings as applicable (hematoxylin and eosin).

We performed immunohistochemical staining for IBA1 on tissue sections. In brief, we conducted antigen retrieval in a pressure cooker at 100°C for 20 min using a citric acid-based antigen unmasking solution (Cat. No. H-3300; Vector Laboratories). Slides were incubated with 3% hydrogen peroxide for 10 min, blocked using avidin/biotin reagents for 15 min each (Cat. No. SP-2001; Vector Laboratories), and incubated with 1% donkey serum with 0.2% Triton-X for 15 min at room temperature. We then incubated the slides with a rabbit anti-IBA1 primary antibody (Cat. No. ab178846; Abcam) diluted 1:2,000 at 4°C overnight.

Next, we incubated the slides with a biotinylated donkey anti-rabbit IgG secondary antibody (Cat. No. 711-065-152; Jackson Laboratory) at a dilution of 1:1,000 for 30 min at room temperature. Slides were washed and then incubated with VECTASTAIN ABC reagent (Cat. No. PK-6100; Vector Laboratories). We performed colorimetric development using a diaminobenzidine kit (Cat. No. SK-4100; Vector Laboratories), followed by counterstaining with hematoxylin and coverslip application.

Following IBA1 immunohistochemistry (IHC) staining, we measured the size of globoid cells using image analysis software. In brief, well-stained and intact regions of sections of the brain, spinal cord, and sciatic nerve were manually outlined using VIS version 2019.07.0.6328 (Visiopharm, Hoersholm, Denmark). We quantitated the IBA1-positive area via thresholding using the IHS-S (Intensity, Hue, Saturation model) classification feature, and we quantified the IBA1-negative area via thresholding using the “HDAB-Hematoxylin” classification feature. The IBA1-positive and IBA1-negative area classifications were used to generate the percentage of the outlined section that was positive for IBA1, the number of IBA1-positive objects, and the average size of all IBA1 objects identified in the section.

For the NHP toxicology study, a board-certified veterinary pathologist who was blinded to the vector group established severity grades as previously published, with 0 defined as an absence of lesions, 1 as minimal (<10%), 2 as mild (10–25%), 3 as moderate (25–50%), 4 as marked (50–95%), and 5 as severe (>95%).^48^ We established dorsal axonopathy scores in each animal from at least three cervical, three thoracic, and three lumbar sections and established the DRG severity grades from at least three cervical, three thoracic, and three lumbar segments.

### GALC enzyme activity

We measured GALC enzyme activity in CSF, serum, and tissue lysates using a commercial kit from Marker Gene Technologies, Inc., (Cat. No. M2774; now available through abcam ab253371). For this assay, we mixed 10 μL of serum or CSF or 50 μg of total protein from tissue lysates with the reaction buffer included in the kit for a final volume of 100 μL. A tube containing 100 μL of reaction buffer served as a blank. We incubated the samples at 37°C for 2 h and stopped the reaction by adding 1 mL of the stop solution supplied with the kit. Finally, we transferred 300 μL of each reaction to a black 96-well plate and measured the fluorescence using a plate reader (emission wavelength: 454 nm, excitation wavelength: 365 nm).

We tested the sensitivity and specificity of the kit using recombinant human GALC (7310-GH; R&D Systems) and recombinant human beta-galactosidase (6464-GH; R&D Systems). Both enzymes were able to cleave the fluorescent substrate under the manufacturer's assay conditions. The assay has an 8.7-fold higher sensitivity toward recombinant GALC than recombinant beta-galactosidase; the linear range for hGALC was from 9.76 pg/μL to 2.5 ng/μL.

### Psychosine quantification in CSF

The Washington University Metabolomics Facility analyzed CSF samples from dogs for glycosphingolipids, including GalCer and galactosylsphingosine (GalS; also referred to as psychosine), as previously described.^56^

### Immunology

We measured NAb titers to AAVhu68 and peripheral blood T cell responses against hGALC and AAVhu68 in NHPs by a neutralizing cell-based assay and by interferon gamma enzyme-linked immunosorbent spot assay (ELISPOT), respectively, according to previously published methods.^57^ Positive response criteria were >55 spot-forming units per 10^6^ lymphocytes and a level equal to three times the medium negative control level in the absence of stimulation, respectively. In addition, we assayed T cell responses in lymphocytes extracted from spleen, liver, bone marrow, and deep cervical lymph nodes after necropsy on study days 90 and 180. We measured antibodies to hGALC in serum (1:1,000 sample dilution), as previously described.^58^

### Viral genome biodistribution

DNA was extracted from tissues using QIAamp columns (Cat. No. 51306; Qiagen). Biodistribution analysis was performed by TaqMan qPCR targeting the vector rabbit beta-globin polyadenylation signal sequence. Assay results were reported as GC per diploid genome (GC/DG).

## RESULTS

### CSF delivery of AAV gene therapy is more efficacious than IV delivery in newborn Twitcher mice

We administered AAVhu68 expressing codon-optimized human *GALC* under control of the ubiquitous promoter CB7 in the neonatal Twitcher mouse model via the IV or ICV route. We administered a single IV high dose of 1 × 10^11^ GC in 50 μL and a dose range (2 × 10^10^ GC in 2 μL, 5 × 10^10^ GC in 2 μL, or 1 × 10^11^ GC in 2 × 2 μL) via uni- or bilateral ICV (high dose only) to presymptomatic postnatal day 0 (PND0) Twitcher mice. Animals were euthanized upon reaching a humane endpoint defined by weight loss >20% of maximal body weight and/or hind leg paralysis.

**Figure 1.**
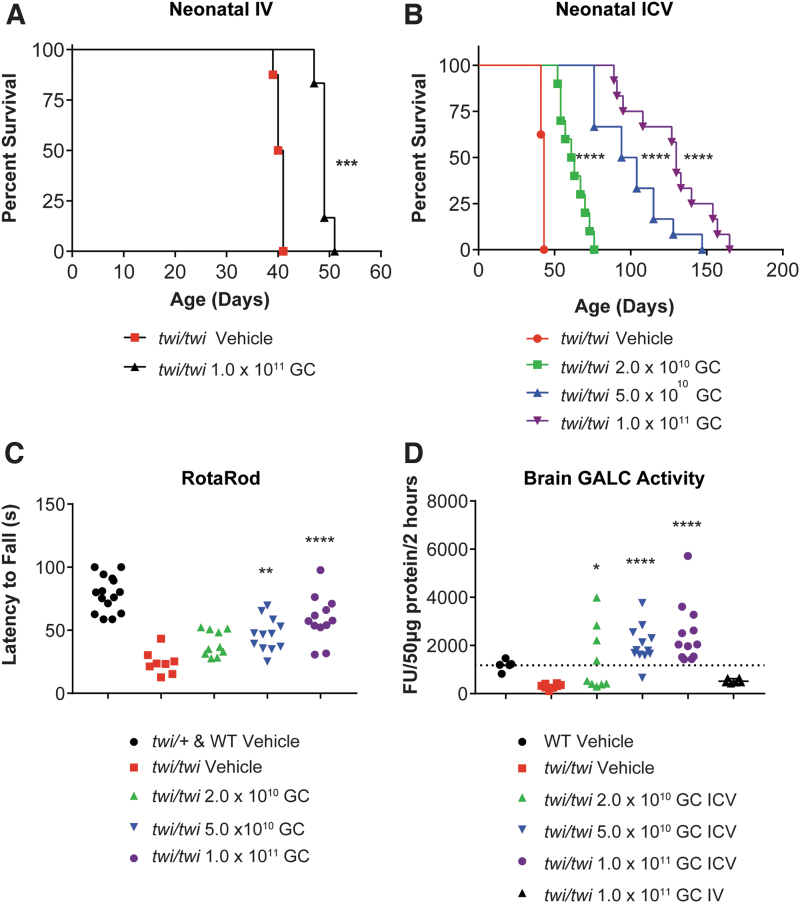
Proof-of-concept efficacy in neonatal Twitcher mice. **(A)** Survival curve of Twitcher mice injected intravenously on PND0 with 50 μL of PBS (vehicle, *n* = 8; median survival 40.5 days) or with 1 × 10^11^ GC of AAVhu68.CB7.hGALCco.rBG diluted in 50 μL PBS (*n* = 6; median survival 49 days). ***p < 0.001 Log-rank (Mantel-Cox) test. **(B)** Survival curve of Twitcher mice injected in the lateral cerebral ventricle (ICV) on PND0 with 2 μL of PBS (vehicle, *n* = 8; median survival 43 days), with 2 × 10^10^ GC of AAVhu68.CB7.hGALCco.rBG in 2 μL PBS (low dose, *n* = 10; median survival 62 days), with 5 × 10^10^ GC of AAVhu68.CB7.hGALCco.rBG in 2 μL PBS (mid dose, *n* = 12; median survival 99 days), or with 1 × 10^11^ GC of AAVhu68.CB7.hGALCco.rBG in 2 × 2 μL PBS (bilateral ICV, high dose, *n* = 12; median survival 130 days). ****p < 0.0001 Log-rank (Mantel-Cox) test. **(C)** Neuromotor function assessed by the accelerated rotarod test on PND35 in mice treated via neonatal ICV administration [same animals as in **(B)**]. **p < 0.01, ***p < 0.001, ****p < 0.0001, Kruskal-Wallis test followed by *post hoc* Dunn's multiple comparison test, alpha = 0.05, comparison to vehicle Twitcher mice. **(D)** GALC enzyme activity in brain lysate from tissue obtained at humane endpoint in animals administered IV or ICV [same animals as in **(A, B)**]. *p < 0.05, ****p < 0.0001, Kruskal-Wallis test followed by *post hoc* Dunn's multiple comparison test, alpha = 0.05, comparison to vehicle Twitcher mice. GALC, galactosylceramidase; GC, genome copies; ICV, intracerebroventricular; IV, intravenous; PBS, phosphate-buffered saline; PND, postnatal day.

High-dose IV administration (1 × 10^11^ GC, equivalent to 1 × 10^14^ GC/kg) increased median survival from 40.5 days in vehicle-treated mice to 49 days in AAV-treated mice (Fig. 1A). A five-fold lower dose, 2 × 10^10^ GC (1.3 × 10^11^ GC/g brain), administered ICV produced a greater survival benefit (62 days median survival); the high dose of 1 × 10^11^ GC (6.7 × 10^11^ GC/g brain) administered ICV achieved the longest median survival of 130 days (minimum 89 days, maximum 165 days; Fig. 1B).

In addition, ICV-treated mice administered mid- and high-dose vector performed significantly better on the rotarod assay at PND35 than vehicle controls, showing improved neuromotor function (Fig. 1C). This improvement correlated with a dose-dependent increase in brain GALC activity after ICV treatment with average values from 117% of WT activity at the low dose to 210% of WT activity at the high dose; IV-treated mice had 44% of WT brain GALC activity (Fig. 1D).

### Pharmacology study of CSF AAV gene therapy in juvenile Twitcher mice

We next investigated whether our therapeutic strategy would benefit mice treated shortly after disease onset at PND12–14. Consistent with published reports, at that disease onset age, Twitcher mice start to exhibit globoid cell infiltration in the peripheral nerves and spinal cord, as evidenced by IBA1 quantification from immunostained sections, whereas cortex, cerebellum, and brainstem are not yet significantly affected^59^ (Supplementary Fig. S1). We selected this age for our minimal efficacy dose-finding, IND-enabling study to model a translationally feasible and physiologically relevant therapeutic window. The brain myelin maturation of a PND12 mouse corresponds to a 2-month-old infant (postconception day 337), whereas the brain maturation of newborn mice is equivalent to that of a second trimester fetus (http://translatingtime.org/translate).^60^

We evaluated four doses of AAVhu68.hGALCco administered via unilateral ICV injection in a fixed volume of 4 μL in 16–17 animals per group. We sacrificed one cohort of each dose at PND40 and kept the other cohort until humane endpoint. The three highest doses resulted in a significant dose-dependent increase in survival compared with vehicle treatment in the Twitcher mice. The median survival age was 40.5 days for the vehicle-treated group, 44.5 days for the 6.8 × 10^9^ GC group (1.7 × 10^10^ GC/g brain; not significantly different), 48 days for the 2.0 × 10^10^ GC group (5.0 × 10^10^ GC/g brain; log rank test, *p* < 0.05), 56.5 days for the 6.8 × 10^10^ GC group (1.7 × 10^11^ GC/g brain; log rank test, *p* < 0.001), and 70 days for the 2.0 × 10^11^ GC group (5.0 × 10^11^ GC/g brain; log rank test, *p* < 0.01).

**Figure 2.**
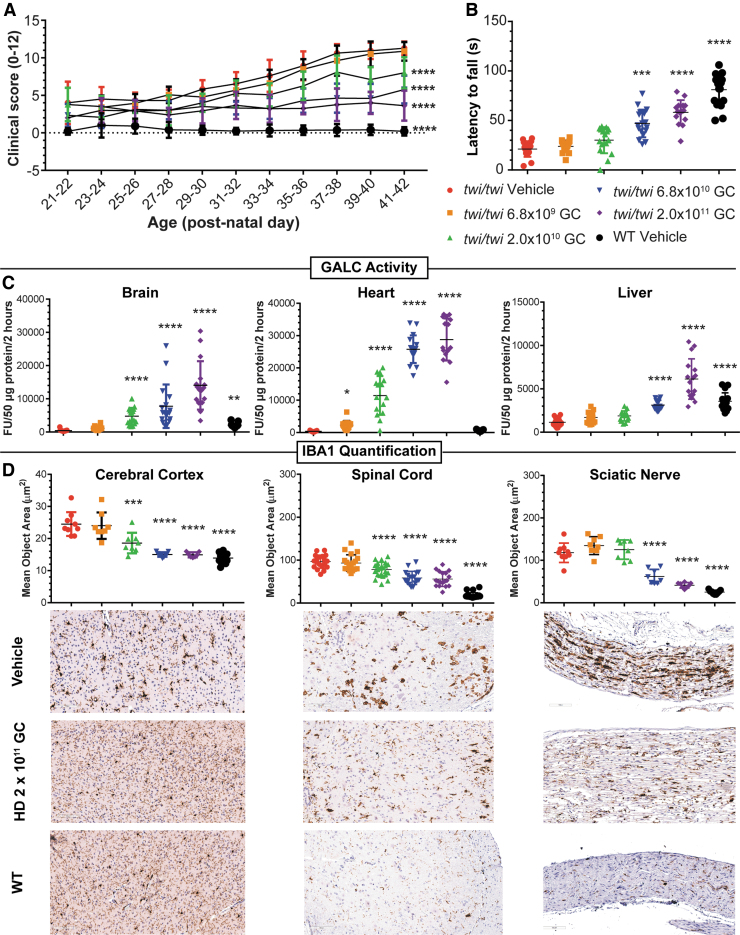
MED study in juvenile postdisease onset Twitcher mice. **(A)** Compound clinical severity score in Twitcher mice treated on PND12–14 ICV with either 4 μL of artificial CSF (vehicle, *n* = 17), or 4 μL of AAVhu68.CB7.hGALCco.rBG at the following doses: 6.8 × 10^9^ GC (*n* = 16), 2 × 10^10^ GC (*n* = 17), 6.8 × 10^10^ (*n* = 17), or 2 × 10^11^ GC (*n* = 16). WT littermates were treated ICV with 4 μL of artificial CSF (*n* = 17). The operator was blinded to the mice genotype and treatment. ****p < 0.0001 linear mixed-effect modeling comparing the clinical score change over time compared to the vehicle Twitcher group, alpha = 0.05. **(B)** Neuromotor function assessed by the accelerated rotarod test on PND35 in same mice as in **(A)** **p < 0.01, ***p < 0.001, ****p < 0.0001, Kruskal-Wallis test followed by *post hoc* Dunn's multiple comparison test, alpha = 0.05, comparison to vehicle Twitcher mice. **(C)** GALC enzyme activity in brain (sagittal half), heart (sagittal half), and liver (half of the left lobe) lysate from tissue obtained either at scheduled necropsy (PND40, half of the animals) or humane endpoint (half of the animals) in the same animals as in **(A)** *p < 0.05, ****p < 0.0001, Kruskal-Wallis test followed by *post hoc* Dunn's multiple comparison test, alpha = 0.05, comparison to vehicle Twitcher mice. **(D)** Neuroinflammation quantification in the cerebral cortex, spinal cord, and sciatic nerve measured by mean object area of IBA1-positive cells stained by immunohistochemistry in tissues collected at the fixed necropsy time PND40. ***p < 0.001, ****p < 0.0001, Kruskal-Wallis test followed by *post hoc* Dunn's multiple comparison test, alpha = 0.05, comparison to vehicle-treated Twitcher mice. Representative images of IBA1 IHC from the vehicle groups (Twitcher and WT mice) and the HD group (2 × 10^11^ GC, ICV PND12–14). CSF, cerebrospinal fluid; MED, minimum effective dose; WT, wild type.

Consistent with the survival data, the minimal efficacy dose of 2.0 × 10^10^ GC (5.0 × 10^10^ GC/g brain) achieved significant prevention of neurological decline (Fig. 2A), a significant increase in brain and heart GALC to 244% and 1,833% of WT activity, respectively (Fig. 2C), and a significant decrease in neuroinflammation in the cerebral cortex and spinal cord when compared to vehicle-treated mice (Fig. 2D). A 3.4-fold higher dose was necessary to significantly preserve neuromotor function on the rotarod assay (Fig. 2B) and improve neuroinflammation in the sciatic nerve (Fig. 2D), probably due to more limited distribution of the vector in the lumbar spinal cord after ICV administration to mice.

Treatment normalized somatic pathology in Twitcher mice, preventing wasting, thymic atrophy, and lymphopenia; it also normalized hepatocellular microvacuolation (Supplementary Fig. S2).

### CSF AAV gene therapy as a monotherapy is efficacious in a canine model of Krabbe disease

Due to the limitations of murine models, particularly the Twitcher mouse, we tested the scalability of our approach in the canine model of Krabbe disease using ICM administration (*i.e*., the intended clinical route). We enrolled seven dogs from three litters in the study: one vehicle-treated WT; two vehicle-treated Krabbe; and four AAV-treated Krabbe dogs. The test article was similar to that used in the mouse studies (*i.e*., capsid, promoter, codon optimization), except that it expressed canine *GALC*.

We treated dogs at 2–3 weeks of age with a single ICM administration of either vehicle or AAV at a dose of 3.0 × 10^13^ GC (6.0 × 10^11^ GC/g brain based on an estimated brain weight of 50 g^61^) in a fixed volume of 1 mL, after 0.5 mL of CSF was removed. This dose was equivalent to the highest dose evaluated in our mouse studies and close to the maximal feasible dose based on the safest volume for CSF administration. The dogs did not receive any immune suppression or anti-inflammatory regimen.

We monitored the two vehicle-treated Krabbe dogs and two of the four AAV-treated dogs until humane endpoint, while the remaining two AAV-treated dogs were euthanized at a scheduled time point of 6 months posttreatment. There were no test-article-related abnormalities in serum chemistry, cell blood counts, or CSF chemistry; minimal CSF mononuclear pleocytosis with 6–10 cells/μL was transiently observed 1 month postdosing in two AAV-treated Krabbe dogs (K933, K938—see Supplementary Data S1).

Consistent with the natural history of the model,^62^ the two vehicle-treated dogs reached the protocol-defined endpoint of extreme hindleg weakness at the age of 8 weeks (K930) and 12 weeks (K948), respectively. K930 had to be euthanized before its first neurological examination. K948 demonstrated severe ataxia (grade 3), proprioceptive deficits, mild tremors (grade 1), decreased spinal reflexes, absent menace reflex (suggesting blindness), and muscle atrophy at the first neurological scoring done at 11 weeks of age (Supplementary Table S2).

Conversely, none of the four AAV-treated dogs presented the classical signs of Krabbe disease and they remained ambulatory with no or minimal ataxia (grade 1—also seen intermittently in WT control), normal proprioception, no tremor, normal spinal and menace reflexes, and no muscle atrophy for the entire study duration (Supplementary Table S2).

**Figure 3.**
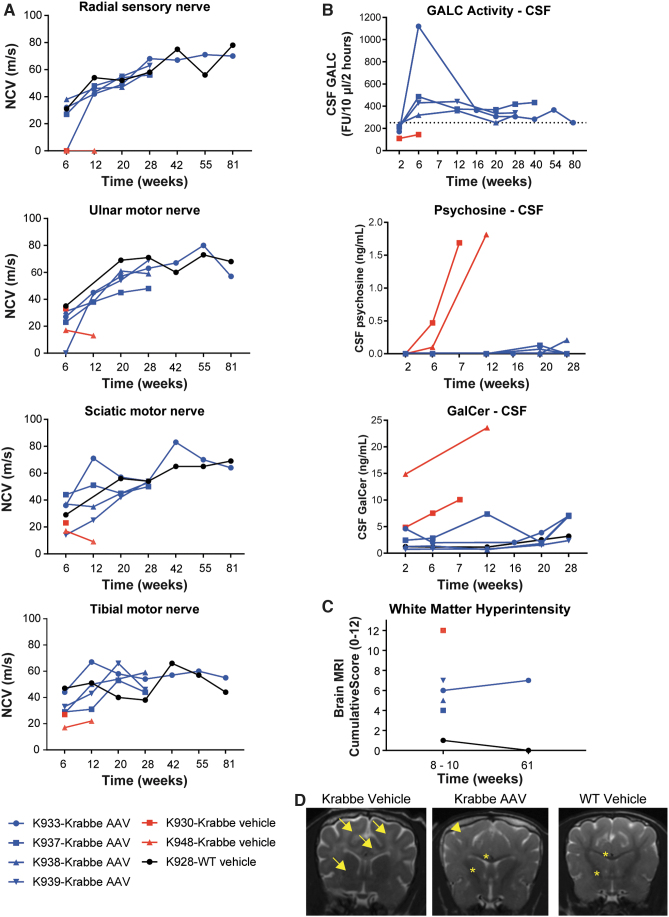
ICM efficacy study in Krabbe dogs, nerve-conduction studies, CSF biomarkers, and MRI. **(A)** Nerve-conduction velocities in the radial (sensory), sciatic (motor), ulnar (motor), and tibial (motor) nerves in 2- to 3-week-old Krabbe dogs treated ICM with either (1) 1 mL of artificial CSF (vehicle, *n* = 2); or (2) 3 × 10^13^ GC of AAVhu68.CB7.cGAMCco.rBG in 1 mL (*n* = 4). A WT littermate that received 1 mL of artificial CSF ICM was used as a control. Two treated dogs were sacrificed at the scheduled timepoint of 6 months postinjection for tissue collection. Two treated dogs and the vehicle-treated Krabbe dogs were followed until humane endpoint. **(B)** Quantification of CSF psychosine and GalCer levels and CSF GALC enzyme activity. The *dotted line* represent the average GALC activity value from the serial CSF timepoints of the WT control (K928). **(C)** Brain MRI WM intensity semiquantitative scoring. **(D)** Brain MRI examples from one Krabbe vehicle dog (8 weeks), one Krabbe dog treated with AAV (10 weeks), and one WT control littermate (10 weeks). A dark hypointense WM represents normal myelination (*stars*); a grey isointense signal represents mild demyelination (*arrowhead*); a white hyperintense signal represents marked demyelination (*arrows*). AAV, adeno-associated virus; GalCer, galactosylceramide; ICM, intracisterna magna; MRI, magnetic resonance imaging; WM, white matter.

The four AAV-treated dogs showed similar results on biweekly neurobehavioral assessment (Supplementary Videos S1–S3) and on neurological scoring (Supplementary Table S2) as the WT littermates. Myelination was preserved in the PNS and in the medulla, as seen by normal velocities on nerve conduction studies (Fig. 3A) and normal interpeak latency on brainstem auditory evoked responses (Supplementary Fig. S3), respectively. AAV treatment prevented the accumulation of psychosine and GalCer in the CSF and increased GALC to levels equivalent to or higher than WT (Fig. 3B).

Brain MRI revealed the expected T2-weighted hyperintensity of white matter indicative of extensive demyelination in the two vehicle-treated Krabbe dogs (Fig. 3D). All AAV-treated Krabbe dogs exhibited lower cumulative white matter hyperintensity scores than vehicle-treated Krabbe dogs at 10 (*n* = 4/4) and 61 weeks of age (*n* = 1/1; Fig. 3C). Some regions, such as the corpus callosum, internal capsule (Fig. 3D), and cerebellar white matter, showed a normal hypointense signal on T2-weighted images, whereas the subcortical white matter showed an isointense signal indicative of mild demyelination (Fig. 3D, arrowhead).

At 61 weeks of age, the single AAV-treated Krabbe dog that underwent a second brain MRI (K933) exhibited a similar cumulative white matter hyperintensity score as the score it received at 8 weeks of age, thereby indicating no further progression of demyelination.

**Figure 4.**
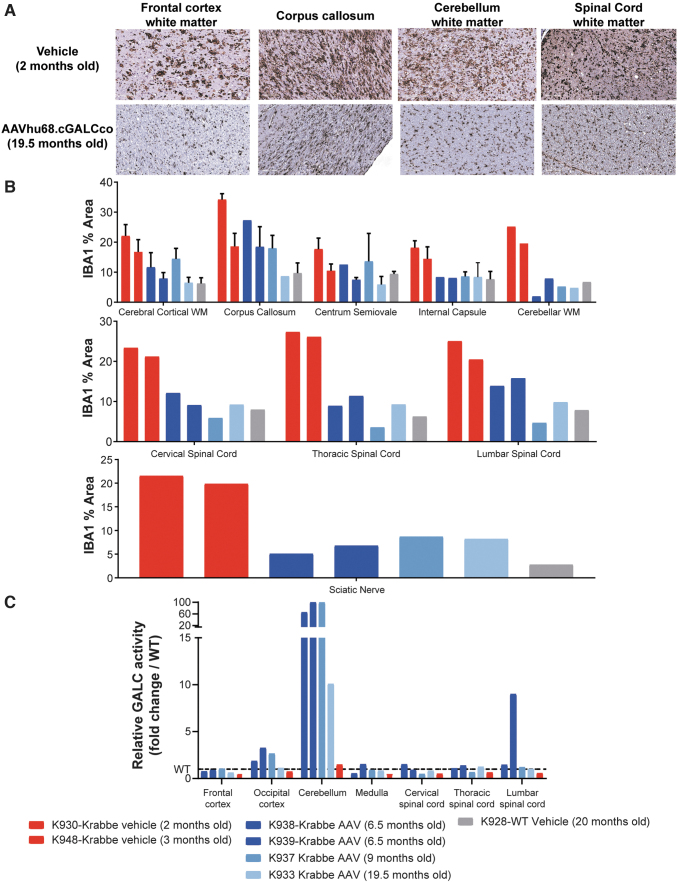
ICM efficacy study in Krabbe dogs, neuroinflammation and GALC levels. **(A)** Representative pictures of IBA1 staining by IHC illustrating the macrophage/microglial neuroinflammation in Krabbe dogs treated with vehicle control or with AAV ICM. **(B)** Quantification of the mean area of the IBA1-positive signal in different neuroanatomical regions of the brain, spinal cord, and sciatic nerve of Krabbe dogs treated with AAV or vehicle. Each bar represents an animal. Error bars when present (cerebral cortical WM, corpus callosum, centrum semiovale, and internal capsule), represent the standard deviation from quantification of multiple slides when the neuroanatomical region was present on more than one brain section. **(C)** GALC activity in tissue lysate (50 μg protein per reaction, 2 h incubation) relative to the WT control (*dotted line*). Each bar represents one animal. WM, white matter.

At 6 months posttreatment, we euthanized two animals for tissue collection and analysis. The two dogs enrolled in the long-term follow-up study were euthanized for animal welfare compliance. One dog presented with an episode of marked hyperthermia and suspected seizure-like activity at the age of 9.5 months (K937). His bloodwork (Supplementary Data S1) showed a neutrophilic leukocytosis (neutrophils = 16,647 cells/μL), lymphopenia (lymphocytes = 537 cells/μL), and increased levels of aspartate aminotransferase (184 IU/L), fibrinogen (455 mg/dL), and D-dimers (>54,000 ng/mL). Blood culture showed the presence of methicillin-resistant *Staphylococcus epidermidis*. Brain histology was similar to the other AAV-treated Krabbe dogs (Fig. 4 and Supplementary Fig. S6).

The longest-surviving dog (K933) was euthanized at 19.5 months due to marked body weight loss caused by recurrent regurgitation and feeding difficulties that were attributed to esophageal dysfunction and bilateral sialoadenitis. Although not part of the typical Krabbe disease presentation in the well-characterized colony, dysphagia could be part of a protracted manifestation of Krabbe disease in animals that live longer due to gene therapy.

Viral vector biodistribution showed the highest transduction in the CNS (0.1–1.8 GC/DG in cortex, 0.05–0.5 GC/DG in medulla, 0.01–0.1 GC/DG in cerebellum, and 0.1–1 GC/DG in spinal cord), followed by DRG (0.01–0.4 GC/DG), and then the peripheral organs (Supplementary Fig. S4). No test article-related histopathological adverse findings were observed, including in DRG collected from cervical, thoracic, and lumbar regions or in corresponding sensory spinal cord ascending white matter tracts (Supplementary Fig. S5).

All four AAV-treated dogs displayed improved myelination on histology by Luxol blue staining. Some regions (*e.g*., the peripheral nerves, spinal cord white matter, cerebellar white matter) were similar to the WT control, whereas other brain regions such as the subcortical white matter and corpus callosum had moderate demyelination and globoid cell infiltration compared to more severe lesions in vehicle-treated Krabbe dogs (Supplementary Fig. S6).

We quantified neuroinflammation using IBA1 IHC followed by whole-slide scanning and unbiased automated signal quantification. Consistent with brain MRI and myelin staining, neuroinflammation was reduced in AAV-treated Krabbe dogs compared to the younger vehicle controls (Fig. 4A).

The sciatic nerve, spinal cord, cerebellar white matter, and internal capsule were similar in vehicle-treated WT and AAV-treated Krabbe animals; the centrum semiovale, corpus callosum, and cerebral subcortical white matter were not normalized and displayed intermediate neuroinflammation (Fig. 4B). GALC activity was the highest in the cerebellum (10 to 100-fold increase over WT), lumbar spinal cord (1 to 9-fold increase over WT), and the occipital cortex (1 to 3.3-fold over WT), while the frontal cortex, medulla, cervical, and thoracic spinal cord had levels equivalent to WT (Fig. 4C).

### ICM AAVhu68.hGALCco is well tolerated in NHPs

**Figure 5.**
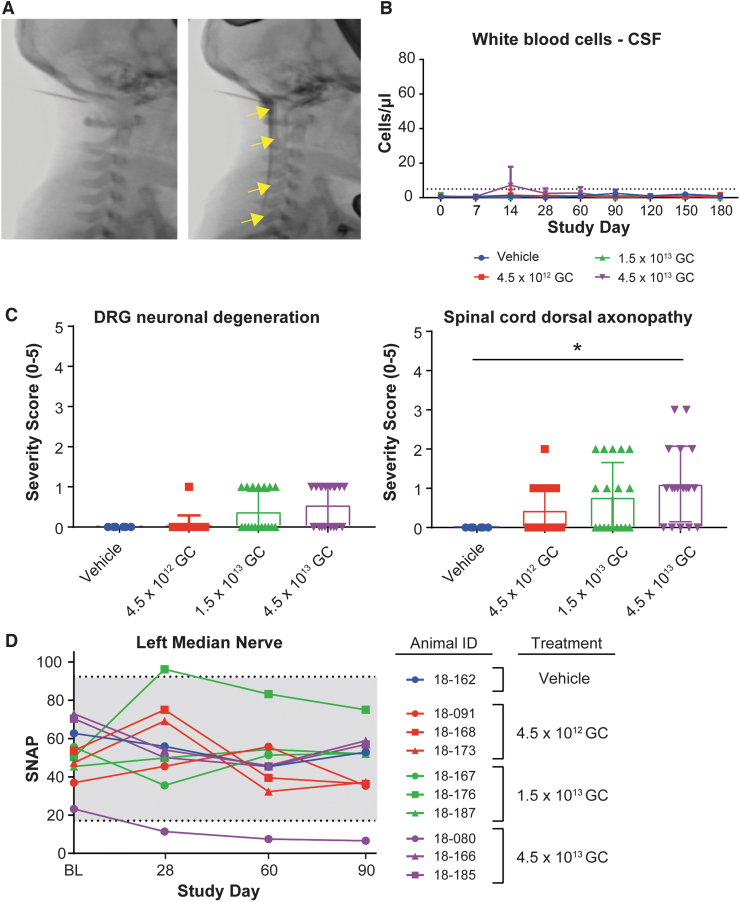
GLP toxicology study in rhesus macaques, safety. **(A)** Fluoroscopic images of the neck region, before and after contrast ICM administration, in juvenile rhesus macaques (animal 18–167, mid-dose group). Contrast injection was used to confirm needle placement before AAV administration. *Arrows* show the diffusion of the contrast in the cisterna magna and spinal canal. **(B)** WBC counts in the CSF of juvenile rhesus macaques treated ICM with artificial CSF (vehicle, *n* = 2) or AAVhu68.CB7.hGALCco.rBG at the dose of 4.5 × 10^12^ GC (low dose, *n* = 6), 1.5 × 10^13^ GC (mid dose, *n* = 6), or 4.5 × 10^13^ (high dose, *n* = 6). Half of the animals were euthanized for tissue collection 3 months postdosing; the other half were euthanized 6 months postdosing. *Dotted line* represents the upper limit of normal **(C)** Histopathological analysis, DRG neuronal degeneration, and spinal cord dorsal column axonopathy severity grades (0 = normal, 1 = minimal, 2 = mild, 3 = moderate, 4 = marked, and 5 = severe). Three and six months cohorts are included, each data point represents 1 segment per animal (cervical, thoracic, and lumbar). *p < 0.05 Mann-Whitney rank test, alpha = 0.05. **(D)** Median nerve SNAP amplitudes at BL (before dosing), 28-, 60-, and 90-day postdosing in the 3 months cohorts. BL, baseline; DRG, dorsal root ganglia; GLP, Good Laboratory Practice; SNAP, sensory nerve action potential; WBC, white blood cell.

We conducted a GLP toxicology study to evaluate the toxicology of AAVhu68.hGALCco. Juvenile male and female rhesus macaques received a single ICM administration of vehicle (artificial CSF; *n* = 2) or AAV at a dose of 4.5 × 10^12^ GC (low dose [LD]; 5.0 × 10^10^ GC/g brain; *n* = 6), 1.5 × 10^13^ GC (mid-dose [MD]; 1.7 × 10^11^ GC/g brain; *n* = 6), or 4.5 × 10^13^ GC (high dose [HD]; 5.0 × 10^11^ GC/g brain; *n* = 6) without immune-suppression treatment. We euthanized half of the animals at 3 months postdosing and the other half at 6 months postdosing. We confirmed spinal needle placement before vector dosing via ICM-administered contrast under fluoroscopy (Fig. 5A).

In-life evaluations included clinical observations, physical examinations, standardized neurological monitoring, sensory nerve conduction studies, body weight measurement, clinical pathology of the blood and CSF (Supplementary Data S2), evaluation of serum-circulating NAbs to AAVhu68, and evaluation of transgene product expression (*i.e*., GALC enzyme activity) and presence of antibodies against the transgene product (*i.e*., anti-human GALC antibodies) in CSF and serum. At day 90 or 180, we harvested tissues for a comprehensive histopathological examination and measurement of T cell responses to the vector capsid and transgene product.

ICM AAVhu68.hGALCco did not cause adverse effects based on clinical and behavioral signs, body weight (Supplementary Fig. S7), or neurologic or physical examinations at any dose. The only treatment-related clinical pathology finding was a mild transient increase in CSF leukocytes on day 14 in some HD group animals that were asymptomatic (Fig. 5B and Supplementary Data S2).

Test article administration resulted in minimal sporadic degeneration of primarily DRG sensory neurons, which led to secondary degeneration of associated central and peripheral axons (*i.e*., axonopathy). The DRG lesions were absent in some test article-treated animals and were minimal in severity when present. Secondary axonopathy associated with DRG lesions was minimal to mild when present, with many sections showing no findings (Fig. 5C). The secondary dorsal axonopathy was dose dependent, with the HD group showing the most histological findings (4.5 × 10^13^ GC [5.0 × 10^11^ GC/g brain]; Fig. 5C).

The incidence and severity were similar at days 90 and 180, showing a lack of pathology progression consistent with prior reports of AAV-mediated DRG pathology.^48^ Findings were asymptomatic (*i.e*., no abnormalities observed clinically or in physical and neurological examinations).

We used electrophysiology to longitudinally record nerve conduction and found decreased SNAPs from the left median nerve (amplitude down from 23.2 μV baseline to 6.6 μV) in one HD animal (Fig. 5D). This correlated with a grade 3 dorsal column axonopathy and a grade 2 perineurial fibrosis in the left median nerve of this individual. The other animals with normal histopathology or grade 1 to grade 2 findings had SNAP amplitudes within the normal range. Although more studies are needed to draw conclusions, these findings suggest that SNAP recording may be able to detect asymptomatic DRG pathology when it affects the segment innervating the tested nerve(s).

Preexisting NAbs to the vector capsid were detectable in baseline sera from 11/18 AAV-treated animals (titers 1:5–1:160). Dose-independent NAb responses to the AAVhu68 capsid were observed in all AAV-treated animals by day 28 until the terminal time point. Titers at necropsy (D90 or D180) ranged from 1:160 to 1:2,560 (Supplementary Table S3) and did not correspond to abnormal clinical or histopathological findings.

Transgene expression (*i.e*., GALC enzyme activity) was detectable in the sera of animals from all dose groups by the first time point evaluated; average GALC activity was 2.1-fold higher than vehicle controls at the LD, 5.7-fold at the MD, and 6.6-fold at the HD (Supplementary Fig. S8A). This is consistent with previous reports of hepatic transduction following ICM administration.^50,51^ However, we observed rapid loss of measurable transgene product activity, which was likely partly attributable to an NHP antibody response to the foreign human transgene product (Supplementary Fig. S8B).

This humoral response to the foreign human transgene product was not associated with abnormal clinical or histopathological findings. Enzyme activity and antibody responses to hGALC were also seen in the CSF (Supplementary Fig. S8).

We detected low-level T cell responses to the vector capsid in some AAV-treated animals (6/18) in peripheral blood mononuclear cells (PBMCs) and/or liver lymphocytes (Supplementary Table S4); these were not affected by the presence of preexisting NAbs to the vector capsid. T cell responses to the human transgene product were more frequent and stronger than to the vector capsid, with responses in 14/18 NHPs in PBMCs and/or lymphocytes isolated from deep cervical lymph nodes, spleen, bone marrow, and liver (Table 1). T cell responses to the vector capsid or transgene were generally not associated with abnormal clinical or histopathological findings.

**Table 1.** ELISPOT responses to the transgene product, nonhuman primate toxicology study

hGALC Peptide Pools	PBMC							Terminal			
	D0	D28	D60	D90	D120	D150	D180	Lymph Node	Spleen	Bone Marrow	Liver
Low dose											
18-091	—	—	—	—	N/A	N/A	N/A	—	—	—	—
18-168	—	93	—	—	N/A	N/A	N/A	—	—	—	123
18-173	—	—	—	—	N/A	N/A	N/A	—	—	—	—
18-042	—	—	—	83	—	—	—	—	—	—	—
18-121	—	—	—	—	—	—	—	—	—	—	—
18-171	—	203	—	—	—	—	—	—	—	—	—
Middle dose											
18-167	—	—	—	—	N/A	N/A	N/A	—	—	—	—
18-176	—	135	—	—	N/A	N/A	N/A	—	83	83	—
18-187	—	123	—	—	N/A	N/A	N/A	80	—	—	705
18-055	—	—	—	—	—	—	—	—	—	—	130
18-181	—	78	—	—	—	—	—	95	100	—	83
18-183	—	138	225	180	95	58	—	—	163	—	843
High dose											
18-080	—	108	—	—	N/A	N/A	N/A	—	—	—	180
18-166	—	—	—	—	N/A	N/A	N/A	70	—	—	—
18-185	—	—	—	—	N/A	N/A	N/A	—	—	—	150
18-038	—	170	118	—	58	—	—	—	—	—	413
18-158	—	—	103	—	—	—	—	—	—	—	403
18-170	—	85	155	65	—	—	—	—	—	—	535
Ctrl											
18-162	—	—	—	—	N/A	N/A	N/A	—	—	—	—
18-159	—	—	—	—	—	—	—	—	—	—	188

For positive responses, the number indicates the average SFU per million cells from duplicate analysis. We used three peptide pools to stimulate the cells. In cases when multiple pools generated a positive response, we reported only the highest value.

PBMC, peripheral blood mononuclear cell; SFU, spot-forming units.

## DISCUSSION

Our aim was to develop an AAV-based drug product for the treatment of infantile Krabbe disease. We selected AAVhu68 capsid, a natural clade F variant, close to AAV9, that presents widespread diffusion and neurotropism after CSF administration.^39,46,63^ Dose-finding and route of administration studies in newborn Twitcher mice demonstrated that AAVhu68.hGALC administration leads to dose-dependent survival up to 165 days after ICV injection of 1 × 10^11^ GC, outperforming IV administration at the same dose with our study conditions.

We elected for CSF administration and used the juvenile Twitcher mice as our model for formal pharmacology IND-enabling study, demonstrating a minimum effective dose (MED) of 2.0 × 10^10^ GC. A GLP toxicology study using the intended clinical route in juvenile rhesus macaques tested a dose range starting at the MED (scaled by brain weight) up to 10-fold the MED with no dose-limiting toxicity.

Almost two decades of work using AAV in Twitcher mice demonstrated improved lifespan with a variety of approaches. With CSF administration, Rafi *et al.* reported a maximal lifespan of 66 days in newborn Twitcher mice treated ICV with 6 × 10^10^ GC of AAV1.mGALC.^25^ Karumuthil-Melethil *et al.* obtained a median lifespan of 55.5 days when dosing 2 × 10^11^ GC of AAV9.mGALC in PND10–11 mice via the lumbar route.^30^ When comparing similar doses (with the caveat of titering method variability between different laboratories) and injection ages, 5 × 10^10^ GC of AAVhu68.hGALC ICV in newborns achieved up to 147 days survival, while 2 × 10^11^ GC ICV to juvenile PND12 mice achieved a median survival of 70 days.

Notably, using systemic administration of AAVrh10 at a very high dose (4 × 10^14^ GC/kg IV), Rafi *et al.* recently obtained an unprecedented survival of up to 430 days.^29^ Such a result was previously only achieved by the same team when combining lower doses of AAVrh10-GALC with BMT,^29^ or by another group that combined AAV9 ICV administration with BMT and substrate reduction.^33^ This suggests that increasing the dose and using a neurotropic capsid may be sufficient for achieving maximal *GALC* expression in the CNS and full disease prevention in the mouse model, although safety needs to be carefully explored in nonrodent species when using very high doses or combination therapies.

The observation that high-dose AAV may be sufficient without BMT is consistent with recent reports of successful gene therapy in the dog model of Krabbe disease. Using AAVrh10 administered IV at postnatal day 3 followed by ICV at 6 weeks, Bradbury *et al.* obtained a modest dose-dependent increase in survival from 18–22 weeks at 1.2 × 10^12^ GC to 30–43 weeks at 3.8 × 10^13^ GC.^31^ However, these dogs eventually developed the classical Krabbe phenotype, indicating that the optimal dose was likely not reached and that treatment only delayed disease progression.

In agreement with the hypothesis that a higher dose could fully prevent disease in the absence of any other intervention, Bradbury *et al.* reported disease prevention in dogs for at least 2.5 years after ICM administration of AAV9.cGALC at 1 × 10^14^ GC using a dose 3.3-fold higher than the present study.^32^

A fivefold lower dose of 2 × 10^13^ GC, close to our dose of 3 × 10^13^ GC, attenuated the disease with survival up to 35 weeks, similar to the previous work using AAVrh10 at 3.8 × 10^13^ GC (IV+ICV). Although our dog study is limited by a low number of treated dogs (*n* = 4), our results of survival and lack of classical disease signs for up to 85 weeks with AAVhu68.cGALC at 3 × 10^13^ GC ICM suggests the potential for transformative therapeutic benefits at a dose that was safe in two nonrodent species and also highlighted its translationally feasibility. In addition, ICM administration has not been associated with any serious adverse events thus far (NCT03580083, NCT04408625, and NCT04127578).

Furthermore, the potential risk of sensory neuronal degeneration in DRG seems low based on the minimal and sporadic findings from our toxicology study, and the absence of DRG pathology from the dog study using an equivalent construct expressing canine *GALC*. The lack of AAV-related pathology in the DRG in dog models of genetic diseases following intrathecal gene therapy encoding canine transgenes aligns with published work and suggests that dog is less sensitive than NHP.^32,64^

The normalization of peripheral nerve function and histology in our dog study is noteworthy as this result was never achieved with hematopoietic stem cell transplant.^15,16,18,19,65^ We believe that peripheral nerve correction after ICM AAV administration results from robust transduction of DRG sensory neurons and spinal cord lower motor neurons, the axons of which collectively make up peripheral nerves. Full normalization of peripheral nerve function and myelination suggests that neuronal transduction is therapeutic. One possible explanation is that the resultant presence of supraphysiological enzyme levels in axons can cross-correct Schwann cells and endoneurial macrophages to restore normal nerve morphology and function.

Alternatively, GALC enzyme secreted into the CSF could gain access to the endoneurial fluid due to the continuity between the subarachnoid space and the endoneurium of the nerve roots.^66^ Finally, continual resorption of CSF into the systemic circulation may lead to peripheral organ transduction after CSF AAV administration (mainly liver and heart) to provide a peripheral source of secreted enzyme (which manifested as increased serum enzyme levels in the NHP study).

Recent reports questioned the possibility of cross-correction in the PNS of Twitcher mice^8^ or in the CNS of metachromatic leukodystrophy.^67^ In both articles, the authors investigated whether WT macrophages expressing natural levels of enzyme could support cross-correction; they failed to detect either GALC or arylsulfatase A in Schwann cells or oligodendrocytes when normal WT macrophages were present. The method of detection in both cases may not have been sensitive enough to detect low levels of enzyme uptake, which is expected when the source does not overexpress the protein.

Gene therapy likely leads to overexpression by a subset of transduced cells, which then secrete supraphysiological levels of transgene that can support tissue cross-correction but is undetectable in nontransduced cells. This hypothesis is consistent with the remarkable therapeutic benefit of *ex vivo* gene therapy observed in presymptomatic metachromatic leukodystrophy patients.^68^

If macrophages/microglia only acted as a cell-autonomous anti-inflammatory population, the trial's outcome should not be better than traditional HSCT. Similarly, Rafi *et al.* elegantly demonstrated the role of *GALC* expression in donor cells by demonstrating increased survival after BMT when the donors are WT compared to heterozygous mice, and by showing that high-dose AAV-treated mice overexpressed *GALC* in their bone marrow, suggesting transduction of hematopoietic cells *in vivo*.^69^ Finally, macrophages appear to be the most readily cross-corrected cell type via both M6PR and M6PR-independent pathways.^70^ This suggests that macrophages, which migrate to the CNS and PNS, could be cross corrected locally in a context of robust neuronal or astrocytic enzyme expression after ICM gene therapy.

Notably, we achieved less disease prevention and lifespan extension in the ICV-treated newborn or juvenile mice than the ICM-treated dogs. Treated mice developed sciatic nerve demyelination and hindleg weakness, whereas the dogs remained ambulatory until scheduled or unscheduled euthanasia. Twitcher mice exhibit a disproportionate vulnerability to PNS degeneration compared to CNS,^20^ whereas Krabbe dogs recapitulate the combined CNS and PNS demyelination pattern seen in patients.^62,71^

The small size and early phenotype in Twitcher mice limit route and dose options. ICV treatment in newborn and juvenile mice does not achieve as much spinal cord and DRG transduction by AAV compared to ICM or intrathecal administration in large-animal models.^39^ Peripheral nerve correction after ICV in mice is therefore suboptimal, thus enabling disease progression in Twitcher mice. When delivering a sufficient AAV dose with an optimal ICM ROA in the translationally relevant dog model, we and others have shown the remarkable therapeutic benefit of gene therapy as a monotherapy, without the need for HSCT.

We report that a translationally feasible single administration of AAVhu68 expressing GALC into the CSF—without BMT or immune suppression—can mitigate most signs of Krabbe disease in the mouse and canine models. A GLP toxicology study in NHPs confirmed the safety of our approach and paved the way for a first-in-human trial of AAVhu68.hGALC-administered ICM to infantile Krabbe disease patients. In agreement with previously published work, we observed that early intervention, as well as achieving high CNS and PNS transduction, is key to achieving maximal therapeutic benefit. ICM seems to be the most practical route for safely achieving these effects in patients.

## Supplementary Material

Supplemental data

Supplemental data

Supplemental data

Supplemental data

Supplemental data

Supplemental data

Supplemental data

Supplemental data

Supplemental data

Supplemental data

Supplemental data

Supplemental data

Supplemental data

Supplemental data

Supplemental data
