# Supplementary material for: Efficacy and Safety of a Krabbe Disease Gene Therapy
Source: Hum Gene Ther. 2022 May 16;33(9-10):499–517. doi: 10.1089/hum.2021.245 (PMC9142772; doi:10.1089/hum.2021.245)
Supplement: Supplemental data [file Supp_DataS1.pdf]

CLINICAL PATHOLOGY – ABBREVIATIONS

CBC & COAGULATION PANELS		SERUM CHEMISTRY PANEL		OTHER	
**Abbreviation**	**Definition**	**Abbreviation**	**Definition**	**Abbreviation**	**Definition**
APTT	Activated PTT	A/G	Albumin/Globulin Ratio	AAV	AAVhu68.CB7.CI.cGALCco.rBG
HCT	Hematocrit	ALT	Alanine Aminotransferase	CSF	Cerebrospinal Fluid
HGB	Hemoglobin	AST	Aspartate Aminotransferase	F	Female
MCH	Mean Corpuscular Hemoglobin	BUN	Blood Urea Nitrogen	GC	Genome Copies
MCHC	MCH Concentration	CPK	Creatine Phosphokinase	*GALC*	Galactosylceramidase (gene, canine)
MCV	Mean Corpuscular Volume	K	Potassium	ID	Identification Number
PT	Prothrombin Time	NA	Sodium	ITFFB	Intrathecal Final Formulation Buffer
RBC	Red Blood Cell Count			M	Male
WBC	White Blood Cell Count			N/A	Not Applicable

Clinical Pathology - Blood: Hematology Page 2
Clinical Pathology - Blood: Serum Chemistry Page 9
Clinical Pathology - Blood: Coagulation Panel Page 20
Clinical Pathology - Cerebrospinal Fluid page 23

CLINICAL PATHOLOGY – BLOOD: HEMATOLOGY

Group		1		2a/b				3
Treatment		ITFFB		AAV				ITFFB
Dose		N/A		3.0 x 10^{13} GC<br>5.0 x 10^{11} GC/g Brain				N/A
GALC Mutation Status[a]		Homozygous (Krabbe Dog)		Homozygous (Krabbe Dog)				Wild Type
	Study Day[b]	K930 (F)	K948 (M)	K933 (M)	K937 (M)	K938 (M)	K939 (F)	K928 (F)
WBC (10^3/µL)	0	7.4	8.7	6.7	6.8	7.0	5.4	11.2
	14 (+/-1)	9.7	11.6	12.2	15.3	13.0	11.4	12.2
	28 (+/-2)	5.0	5.9	7.4	5.5	6.5	5.5	5.6
	70-98 (+/-3)	N/A	6.0	5.7	10.9	11.0	13.4	5.3
	120 (+/-3)	N/A	N/A	7.0	6.1	7.6	8.6	8.7
	180 (+/-3)	N/A	N/A	6.5	6.7	6.6	9.9	7.2
	9 months	N/A	N/A	6.3	17.9[c]	N/A	N/A	8.9
	12 months	N/A	N/A	7.9	N/A	N/A	N/A	5.7
	18 months	N/A	N/A	8.3	N/A	N/A	N/A	4.9
	19 months	N/A	N/A	7.9[c]	N/A	N/A	N/A	8.0
RBC (10^6/µL)	0	3.4	3.3	3.6	4.0	3.9	3.6	4.0
	14 (+/-1)	5.0	4.7	4.9	4.1	4.4	3.8	5.0
	28 (+/-2)	4.7	4.5	4.4	3.8	3.5	3.5	4.5
	70-98 (+/-3)	N/A	5.7	5.1	4.6	4.6	4.4	5.0
	120 (+/-3)	N/A	N/A	5.2	5.1	5.4	4.9	5.4
	180 (+/-3)	N/A	N/A	5.7	5.6	5.3	5.4	5.9
	9 months	N/A	N/A	6.3	8.3[c]	N/A	N/A	6.0
	12 months	N/A	N/A	10.7	N/A	N/A	N/A	6.2
	18 months	N/A	N/A	7.0	N/A	N/A	N/A	6.9
	19 months	N/A	N/A	9.1[c]	N/A	N/A	N/A	6.6
HGB (g/dL)	0	7.8	8.5	8.0	10.1	10.4	9.7	8.9
	14 (+/-1)	11.0	10.7	9.8	9.2	10.3	9.1	9.9

Group		1		2a/b				3
Treatment		ITFFB		AAV				ITFFB
Dose		N/A		3.0×10^{13} GC<br>5.0×10^{11} GC/g Brain				N/A
GALC Mutation Status[a]		Homozygous<br>(Krabbe Dog)		Homozygous<br>(Krabbe Dog)				Wild Type
	Study Day[b]	K930 (F)	K948 (M)	K933 (M)	K937 (M)	K938 (M)	K939 (F)	K928 (F)
	28 (+/-2)	10.5	9.9	9.3	8.5	8.0	8.3	9.5
	70-98 (+/-3)	N/A	12.5	10.6	10.3	10.5	10.2	10.6
	120 (+/-3)	N/A	N/A	10.8	11.2	12.2	11.4	11.7
	180 (+/-3)	N/A	N/A	12.0	12.4	12.0	13.2	12.4
	9 months	N/A	N/A	14.1	19[c]	N/A	N/A	13.5
	12 months	N/A	N/A	24.2	N/A	N/A	N/A	13.5
	18 months	N/A	N/A	15.2	N/A	N/A	N/A	15.0
	19 months	N/A	N/A	19.6[c]	N/A	N/A	N/A	14.8
HCT (%)	0	26	29	28	34	34	32	30
	14 (+/-1)	36	37	35	32	35	31	35
	28 (+/-2)	33	33	30	29	27	28	31
	70-98 (+/-3)	N/A	40	35	33	32	32	35
	120 (+/-3)	N/A	N/A	35	37	39	36	37
	180 (+/-3)	N/A	N/A	39	41	38	40	41
	9 months	N/A	N/A	43	64[c]	N/A	N/A	43
	12 months	N/A	N/A	75	N/A	N/A	N/A	42
	18 months	N/A	N/A	50	N/A	N/A	N/A	49
	19 months	N/A	N/A	59[c]	N/A	N/A	N/A	44
MCV (fL)	0	78	86	78	86	87	87	75
	14 (+/-1)	71	78	71	78	80	81	70
	28 (+/-2)	70	73	68	77	77	79	69

Group		1		2a/b				3
Treatment		ITFFB		AAV				ITFFB
Dose		N/A		3.0 x 10^{13} GC<br>5.0 x 10^{11} GC/g Brain				N/A
GALC Mutation Status[a]		Homozygous<br>(Krabbe Dog)		Homozygous<br>(Krabbe Dog)				Wild Type
	Study Day[b]	K930 (F)	K948 (M)	K933 (M)	K937 (M)	K938 (M)	K939 (F)	K928 (F)
	70-98 (+/-3)	N/A	70	68	70	70	72	71
	120 (+/-3)	N/A	N/A	67	73	73	73	69
	180 (+/-3)	N/A	N/A	68	73	72	76	69
	9 months	N/A	N/A	68	77[c]	N/A	N/A	72
	12 months	N/A	N/A	70	N/A	N/A	N/A	68
	18 months	N/A	N/A	71	N/A	N/A	N/A	71
	19 months	N/A	N/A	64[c]	N/A	N/A	N/A	68
MCH (pg)	0	23.3	25.4	22.5	25.4	26.6	26.8	22.5
	14 (+/-1)	20.7	22.7	20.2	22.6	23.5	24.0	19.7
	28 (+/-2)	22.2	21.8	21.3	22.4	22.9	23.4	21.0
	70-98 (+/-3)	N/A	22.0	20.7	22.2	22.9	23.1	21.3
	120 (+/-3)	N/A	N/A	20.9	22.3	22.8	23.2	21.7
	180 (+/-3)	N/A	N/A	20.8	22.2	22.7	24.7	21.2
	9 months	N/A	N/A	22.3	22.9[c]	N/A	N/A	22.4
	12 months	N/A	N/A	22.6	N/A	N/A	N/A	22.0
	18 months	N/A	N/A	21.6	N/A	N/A	N/A	21.7
	19 months	N/A	N/A	21.5[c]	N/A	N/A	N/A	22.5
MCHC (g/dL)	0	30	30	29	29	30	31	30
	14 (+/-1)	31	29	28	29	29	29	28
	28 (+/-2)	32	30	31	29	30	30	31
	70-98 (+/-3)	N/A	31	30	32	33	32	30

Group		1		2a/b				3
Treatment		ITFFB		AAV				ITFFB
Dose		N/A		3.0 x 10^{13} GC<br>5.0 x 10^{11} GC/g Brain				N/A
GALC Mutation Status[a]		Homozygous (Krabbe Dog)		Homozygous (Krabbe Dog)				Wild Type
	Study Day[b]	K930 (F)	K948 (M)	K933 (M)	K937 (M)	K938 (M)	K939 (F)	K928 (F)
	120 (+/-3)	N/A	N/A	31	31	31	32	32
	180 (+/-3)	N/A	N/A	31	30	32	33	31
	9 months	N/A	N/A	33	30[c]	N/A	N/A	31
	12 months	N/A	N/A	32	N/A	N/A	N/A	32
	18 months	N/A	N/A	31	N/A	N/A	N/A	31
	19 months	N/A	N/A	33[c]	N/A	N/A	N/A	33
Platelets (10^3/µL)	0	243	331	247	412	423	505	324
	14 (+/-1)	572	462	644	443	521	418	509
	28 (+/-2)	323	249	362	261	285	425	226
	70-98 (+/-3)	N/A	379	400	331	386	542	333
	120 (+/-3)	N/A	N/A	356	369	352	404	210
	180 (+/-3)	N/A	N/A	240	222	261	305	106
	9 months	N/A	N/A	232	180[c]	N/A	N/A	255
	12 months	N/A	N/A	415	N/A	N/A	N/A	199
	18 months	N/A	N/A	189	N/A	N/A	N/A	227
	19 months	N/A	N/A	300[c]	N/A	N/A	N/A	132
Neutrophils (/µL)	0	5328	6177	3752	3876	4130	3078	6048
	14 (+/-1)	5732	7076	7076	9027	6760	5814	7930
	28 (+/-2)	3250	3422	4514	2970	2990	3410	3080
	70-98 (+/-3)	N/A	3660	4047	7739	6710	8844	2597
	120 (+/-3)	N/A	N/A	4620	3416	4028	4730	5394

Group		1		2a/b				3
Treatment		ITFFB		AAV				ITFFB
Dose		N/A		3.0×10^{13} GC<br>5.0×10^{11} GC/g Brain				N/A
GALC Mutation Status[a]		Homozygous (Krabbe Dog)		Homozygous (Krabbe Dog)				Wild Type
	Study Day[b]	K930 (F)	K948 (M)	K933 (M)	K937 (M)	K938 (M)	K939 (F)	K928 (F)
	180 (+/-3)	N/A	N/A	3900	4355	3300	6336	3888
	9 months	N/A	N/A	4284	16647[c]	N/A	N/A	6675
	12 months	N/A	N/A	4977	N/A	N/A	N/A	3477
	18 months	N/A	N/A	6640	N/A	N/A	N/A	3626
	19 months	N/A	N/A	6004[c]	N/A	N/A	N/A	5120
Lymphocytes (/µL)	0	1776	2088	2613	2244	2310	1782	4368
	14 (+/-1)	3783	3712	3660	5355	5590	4674	3782
	28 (+/-2)	1350	1888	2220	1980	2665	1650	2016
	70-98 (+/-3)	N/A	1860	1083	2398	3410	3618	2067
	120 (+/-3)	N/A	N/A	1680	2196	3116	2838	2523
	180 (+/-3)	N/A	N/A	1950	1809	2970	2673	1728
	9 months	N/A	N/A	1323	537[c]	N/A	N/A	1246
	12 months	N/A	N/A	2449	N/A	N/A	N/A	1254
	18 months	N/A	N/A	913	N/A	N/A	N/A	784
	19 months	N/A	N/A	1264[c]	N/A	N/A	N/A	1440
Monocytes (/µL)	0	148	348	134	476	280	324	672
	14 (+/-1)	388	696	732	765	650	684	244
	28 (+/-2)	350	354	518	385	585	220	392
	70-98 (+/-3)	N/A	420	399	654	770	804	318
	120 (+/-3)	N/A	N/A	630	366	380	430	522
	180 (+/-3)	N/A	N/A	390	335	264	495	792
	9 months	N/A	N/A	441	716[c]	N/A	N/A	445

Group		1		2a/b				3
Treatment		ITFFB		AAV				ITFFB
Dose		N/A		3.0 x 10^{13} GC<br>5.0 x 10^{11} GC/g Brain				N/A
GALC Mutation Status[a]		Homozygous (Krabbe Dog)		Homozygous (Krabbe Dog)				Wild Type
	Study Day[b]	K930 (F)	K948 (M)	K933 (M)	K937 (M)	K938 (M)	K939 (F)	K928 (F)
	12 months	N/A	N/A	316	N/A	N/A	N/A	171
	18 months	N/A	N/A	581	N/A	N/A	N/A	196
	19 months	N/A	N/A	553[c]	N/A	N/A	N/A	480
Eosinophils (/µL)	0	148	87	134	204	280	216	112
	14 (+/-1)	97	116	732	153	0	228	244
	28 (+/-2)	50	236	148	165	260	110	112
	70-98 (+/-3)	N/A	60	171	109	110	134	318
	120 (+/-3)	N/A	N/A	70	122	76	602	261
	180 (+/-3)	N/A	N/A	260	201	66	396	792
	9 months	N/A	N/A	252	0[c]	N/A	N/A	534
	12 months	N/A	N/A	158	N/A	N/A	N/A	798
	18 months	N/A	N/A	166	N/A	N/A	N/A	294
	19 months	N/A	N/A	79[c]	N/A	N/A	N/A	960
Basophils (/µL)	0	0	0	67	0	0	0	0
	14 (+/-1)	0	0	0	0	0	0	0
	28 (+/-2)	0	0	0	0	0	110	0
	70-98 (+/-3)	N/A	0	0	0	0	0	0
	120 (+/-3)	N/A	N/A	0	0	0	0	0
	180 (+/-3)	N/A	N/A	0	0	0	0	0
	9 months	N/A	N/A	0	0[c]	N/A	N/A	0
	12 months	N/A	N/A	0	N/A	N/A	N/A	0

Group		1		2a/b				3
Treatment		ITFFB		AAV				ITFFB
Dose		N/A		3.0 x 10^{13} GC<br>5.0 x 10^{11} GC/g Brain				N/A
GALC Mutation Status[a]		Homozygous (Krabbe Dog)		Homozygous (Krabbe Dog)				Wild Type
	Study Day[b]	K930 (F)	K948 (M)	K933 (M)	K937 (M)	K938 (M)	K939 (F)	K928 (F)
	18 months	N/A	N/A	0	N/A	N/A	N/A	0
	19 months	N/A	N/A	0[c]	N/A	N/A	N/A	0

K933, K928 D70 collected late on D98 instead of D70
K948 D70 collected on D66, unscheduled necropsy
[a]Mutation status refers to the canine *GALC* A.C 473 (Y158S) loss-of-function mutation. Animals homozygous for the *GALC* loss-of-function mutation exhibit phenotypes similar to that of human Krabbe disease and are referred to as Krabbe dogs.
[b] Study day is defined as days/weeks post treatment.
[c] Unscheduled terminal bleed.

CLINICAL PATHOLOGY – BLOOD: SERUM CHEMISTRY

Group		1		2a/b				3
Treatment		ITFFB		AAV				ITFFB
Dose		N/A		3.0×10^{13} GC<br>5.0×10^{11} GC/g Brain				N/A
GALC Mutation Status[a]		Homozygous (Krabbe Dog)		Homozygous (Krabbe Dog)				Wild Type
	Study Day[b]	K930 (F)	K948 (M)	K933 (M)	K937 (M)	K938 (M)	K939 (F)	K928 (F)
Total Protein (g/dL)	0	4.0	4.2	4.0	3.5	3.7	3.6	4.3
	14 (+/-1)	4.9	5.0	4.5	4.0	3.9	4.7	4.3
	28 (+/-2)	4.0	4.3	4.3	4.0	4.3	4.3	3.4
	70-98 (+/-3)	N/A	4.8	4.8	4.5	4.5	4.3	4.2
	120 (+/-3)	N/A	N/A	4.8	4.9	4.8	4.4	4.5
	180 (+/-3)	N/A	N/A	4.7	5.1	5.0	4.8	4.6
	9 months	N/A	N/A	5.2	7.1[c]	N/A	N/A	4.9
	12 months	N/A	N/A	5.7	N/A	N/A	N/A	5.2
	18 months	N/A	N/A	6.4	N/A	N/A	N/A	5.9
	19 months	N/A	N/A	7[c]	N/A	N/A	N/A	5.5
Albumin (g/dL)	0	2.6	2.7	2.5	2.5	2.4	2.3	2.9
	14 (+/-1)	3.2	3.8	3.1	2.3	2.7	3.0	2.9
	28 (+/-2)	2.8	3.0	2.9	2.4	2.9	2.8	2.6
	70-98 (+/-3)	N/A	3.3	2.9	3.0	3.0	2.9	2.8
	120 (+/-3)	N/A	N/A	3.0	3.0	3.0	2.9	2.9
	180 (+/-3)	N/A	N/A	2.9	3.0	3.0	2.8	2.9
	9 months	N/A	N/A	3.0	3.9[c]	N/A	N/A	2.8
	12 months	N/A	N/A	3.2	N/A	N/A	N/A	3.3
	18 months	N/A	N/A	3.4	N/A	N/A	N/A	3.5
	19 months	N/A	N/A	3.8[c]	N/A	N/A	N/A	3.2
Globulin (g/dL)	0	1.4	1.5	1.5	1.0	1.3	1.3	1.4
	14 (+/-1)	1.7	1.2	1.4	1.7	1.2	1.7	1.4

Group		1		2a/b				3
Treatment		ITFFB		AAV				ITFFB
Dose		N/A		3.0×10^{13} GC<br>5.0×10^{11} GC/g Brain				N/A
GALC Mutation Status[a]		Homozygous<br>(Krabbe Dog)		Homozygous<br>(Krabbe Dog)				Wild Type
	Study Day[b]	K930 (F)	K948 (M)	K933 (M)	K937 (M)	K938 (M)	K939 (F)	K928 (F)
	28 (+/-2)	1.2	1.3	1.4	1.6	1.4	1.5	0.8
	70-98 (+/-3)	N/A	1.5	1.9	1.5	1.5	1.4	1.4
	120 (+/-3)	N/A	N/A	1.8	1.9	1.8	1.5	1.6
	180 (+/-3)	N/A	N/A	1.8	2.1	2.0	2.0	1.7
	9 months	N/A	N/A	2.2	3.2[c]	N/A	N/A	2.1
	12 months	N/A	N/A	2.5	N/A	N/A	N/A	1.9
	18 months	N/A	N/A	3	N/A	N/A	N/A	2.4
	19 months	N/A	N/A	3.2[c]	N/A	N/A	N/A	2.3
A/G Ratio	0	1.9	1.8	1.7	2.5	1.8	1.8	2.1
	14 (+/-1)	1.9	3.2	2.2	1.4	2.3	1.8	2.1
	28 (+/-2)	2.3	2.3	2.1	1.5	2.1	1.9	3.3
	70-98 (+/-3)	N/A	2.2	1.5	2.0	2.0	2.1	2.0
	120 (+/-3)	N/A	N/A	1.7	1.6	1.7	1.9	1.8
	180 (+/-3)	N/A	N/A	1.6	1.4	1.5	1.4	1.7
	9 months	N/A	N/A	1.4	1.22[c]	N/A	N/A	1.3
	12 months	N/A	N/A	1.3	N/A	N/A	N/A	1.7
	18 months	N/A	N/A	1.1	N/A	N/A	N/A	1.5
	19 months	N/A	N/A	1.2[c]	N/A	N/A	N/A	1.4
AST (IU/L)	0	17	16	15	16	13	12	24
	14 (+/-1)	16	20	20	13	17	15	24
	28 (+/-2)	21	27	25	17	17	15	23

Group		1		2a/b				3
Treatment		ITFFB		AAV				ITFFB
Dose		N/A		3.0 x 10^{13} GC<br>5.0 x 10^{11} GC/g Brain				N/A
GALC Mutation Status[a]		Homozygous (Krabbe Dog)		Homozygous (Krabbe Dog)				Wild Type
	Study Day[b]	K930 (F)	K948 (M)	K933 (M)	K937 (M)	K938 (M)	K939 (F)	K928 (F)
	70-98 (+/-3)	N/A	18	29	19	22	20	27
	120 (+/-3)	N/A	N/A	25	25	26	22	26
	180 (+/-3)	N/A	N/A	26	17	18	19	28
	9 months	N/A	N/A	31	184[c]	N/A	N/A	44
	12 months	N/A	N/A	27	N/A	N/A	N/A	31
	18 months	N/A	N/A	24	N/A	N/A	N/A	33
	19 months	N/A	N/A	55[c]	N/A	N/A	N/A	32
ALT (IU/L)	0	8	6	5	6	7	8	7
	14 (+/-1)	20	15	13	10	12	8	19
	28 (+/-2)	20	36	30	19	26	21	21
	70-98 (+/-3)	N/A	18	20	20	28	25	16
	120 (+/-3)	N/A	N/A	18	23	28	25	16
	180 (+/-3)	N/A	N/A	16	18	30	23	16
	9 months	N/A	N/A	25	48[c]	N/A	N/A	24
	12 months	N/A	N/A	23	N/A	N/A	N/A	23
	18 months	N/A	N/A	17	N/A	N/A	N/A	26
	19 months	N/A	N/A	30[c]	N/A	N/A	N/A	22
Alk P (IU/L)	0	134	172	131	242	216	302	170
	14 (+/-1)	135	263	143	175	143	202	160
	28 (+/-2)	119	245	128	103	88	216	132
	70-98 (+/-3)	N/A	162	167	121	101	157	137

Group		1		2a/b				3
Treatment		ITFFB		AAV				ITFFB
Dose		N/A		3.0×10^{13} GC<br>5.0×10^{11} GC/g Brain				N/A
GALC Mutation Status[a]		Homozygous (Krabbe Dog)		Homozygous (Krabbe Dog)				Wild Type
	Study Day[b]	K930 (F)	K948 (M)	K933 (M)	K937 (M)	K938 (M)	K939 (F)	K928 (F)
	120 (+/-3)	N/A	N/A	118	126	108	130	97
	180 (+/-3)	N/A	N/A	103	81	82	84	76
	9 months	N/A	N/A	78	140[c]	N/A	N/A	37
	12 months	N/A	N/A	55	N/A	N/A	N/A	26
	18 months	N/A	N/A	49	N/A	N/A	N/A	25
	19 months	N/A	N/A	26[c]	N/A	N/A	N/A	25
Total Bilirubin (mg/dL)	0	0.1	0.1	0.1	0.1	0.1	0.1	0.1
	14 (+/-1)	0.1	0.1	0.1	0.1	0.1	0.1	0.2
	28 (+/-2)	0.1	0.1	0.1	0.1	0.1	0.2	0.1
	70-98 (+/-3)	N/A	0.1	0.1	0.5	0.4	0.3	0.2
	120 (+/-3)	N/A	N/A	0.1	0.3	0.2	0.1	0.1
	180 (+/-3)	N/A	N/A	0.1	0.2	0.4	0.2	0.2
	9 months	N/A	N/A	0.1	0.4[c]	N/A	N/A	0.2
	12 months	N/A	N/A	0.1	N/A	N/A	N/A	0.2
	18 months	N/A	N/A	0.2	N/A	N/A	N/A	0.2
	19 months	N/A	N/A	0.1[c]	N/A	N/A	N/A	0.2
BUN (mg/dL)	0	10	21	11	19	16	10	22
	14 (+/-1)	19	11	13	8	9	10	13
	28 (+/-2)	15	11	16	13	16	9	16
	70-98 (+/-3)	N/A	9	18	14	18	15	22
	120 (+/-3)	N/A	N/A	15	13	13	10	15

Group		1		2a/b				3
Treatment		ITFFB		AAV				ITFFB
Dose		N/A		3.0×10^{13} GC<br>5.0×10^{11} GC/g Brain				N/A
GALC Mutation Status[a]		Homozygous (Krabbe Dog)		Homozygous (Krabbe Dog)				Wild Type
	Study Day[b]	K930 (F)	K948 (M)	K933 (M)	K937 (M)	K938 (M)	K939 (F)	K928 (F)
	180 (+/-3)	N/A	N/A	23	14	19	14	17
	9 months	N/A	N/A	18	37[c]	N/A	N/A	19
	12 months	N/A	N/A	36	N/A	N/A	N/A	20
	18 months	N/A	N/A	20	N/A	N/A	N/A	23
	19 months	N/A	N/A	31[c]	N/A	N/A	N/A	27
Creatinine (mg/dL)	0	0.2	0.2	0.2	0.2	0.3	0.2	0.2
	14 (+/-1)	0.8	0.2	0.9	0.5	0.4	0.2	0.8
	28 (+/-2)	0.8	0.4	0.7	0.4	0.6	0.2	0.6
	70-98 (+/-3)	N/A	0.3	0.6	0.5	0.7	0.5	0.7
	120 (+/-3)	N/A	N/A	0.7	0.5	0.6	0.4	0.7
	180 (+/-3)	N/A	N/A	0.8	0.7	0.8	0.5	0.9
	9 months	N/A	N/A	0.8	1.6[c]	N/A	N/A	1.0
	12 months	N/A	N/A	1.1	N/A	N/A	N/A	0.9
	18 months	N/A	N/A	1	N/A	N/A	N/A	1.1
	19 months	N/A	N/A	0.9[c]	N/A	N/A	N/A	1.1
BUN/Creatinine Ratio	0	50	105	55	95	53	50	110
	14 (+/-1)	24	55	14	16	23	50	16
	28 (+/-2)	19	28	23	33	27	45	27
	70-98 (+/-3)	N/A	30	30	28	26	30	31
	120 (+/-3)	N/A	N/A	21	26	22	25	21
	180 (+/-3)	N/A	N/A	29	20	24	28	19
	9 months	N/A	N/A	23	23.1[c]	N/A	N/A	19

Group		1		2a/b				3
Treatment		ITFFB		AAV				ITFFB
Dose		N/A		3.0×10^{13} GC<br>5.0×10^{11} GC/g Brain				N/A
GALC Mutation Status[a]		Homozygous (Krabbe Dog)		Homozygous (Krabbe Dog)				Wild Type
	Study Day[b]	K930 (F)	K948 (M)	K933 (M)	K937 (M)	K938 (M)	K939 (F)	K928 (F)
	12 months	N/A	N/A	33	N/A	N/A	N/A	22
	18 months	N/A	N/A	20	N/A	N/A	N/A	21
	19 months	N/A	N/A	34[c]	N/A	N/A	N/A	25
Phosphorus (mg/dL)	0	8.6	10.8	10.3	10.9	9.5	8.6	9.6
	14 (+/-1)	8.4	8.8	9.4	9.8	8.5	8.6	9.3
	28 (+/-2)	10.3	11.3	10.7	11.5	13.1	10.2	10.2
	70-98 (+/-3)	N/A	12.0	10.3	10.1	10.3	9.3	9.6
	120 (+/-3)	N/A	N/A	9.2	10.2	8.9	8.1	9.1
	180 (+/-3)	N/A	N/A	8.0	9.2	7.9	7.7	6.6
	9 months	N/A	N/A	4.9	3.1c	N/A	N/A	4.9
	12 months	N/A	N/A	8.3	N/A	N/A	N/A	5.8
	18 months	N/A	N/A	4.2	N/A	N/A	N/A	4.1
	19 months	N/A	N/A	5.2[c]	N/A	N/A	N/A	3.3
Glucose (mg/dL)	0	126	166	150	155	155	148	139
	14 (+/-1)	131	154	139	133	150	143	132
	28 (+/-2)	126	128	170	144	135	209	122
	70-98 (+/-3)	N/A	165	148	139	136	119	140
	120 (+/-3)	N/A	N/A	146	117	141	143	128
	180 (+/-3)	N/A	N/A	140	131	131	129	123
	9 months	N/A	N/A	113	159[c]	N/A	N/A	120
	12 months	N/A	N/A	110	N/A	N/A	N/A	118

Group		1		2a/b				3
Treatment		ITFFB		AAV				ITFFB
Dose		N/A		3.0×10^{13} GC<br>5.0×10^{11} GC/g Brain				N/A
GALC Mutation Status[a]		Homozygous (Krabbe Dog)		Homozygous (Krabbe Dog)				Wild Type
	Study Day[b]	K930 (F)	K948 (M)	K933 (M)	K937 (M)	K938 (M)	K939 (F)	K928 (F)
	18 months	N/A	N/A	104	N/A	N/A	N/A	115
	19 months	N/A	N/A	112[c]	N/A	N/A	N/A	116
Calcium (mg/dL)	0	10.6	12.1	10.8	10.9	11.0	11.0	11.2
	14 (+/-1)	11.5	11.2	11.5	11.3	10.8	10.8	11.9
	28 (+/-2)	11.2	10.8	11.1	11.4	11.7	11.1	10.6
	70-98 (+/-3)	N/A	12.3	11.0	11.8	11.2	10.4	10.8
	120 (+/-3)	N/A	N/A	11.1	11.0	10.3	9.9	10.7
	180 (+/-3)	N/A	N/A	10.2	9.5	10.2	10.0	10.0
	9 months	N/A	N/A	9.9	11.2[c]	N/A	N/A	10.1
	12 months	N/A	N/A	10.1	N/A	N/A	N/A	10.2
	18 months	N/A	N/A	10.2	N/A	N/A	N/A	10.2
	19 months	N/A	N/A	11.2[c]	N/A	N/A	N/A	9.1
Corrected Calcium	0	11.5	12.9	11.8	11.9	12.1	12.2	11.8
	14 (+/-1)	11.8	N/A	11.9	12.5	11.6	11.3	12.5
	28 (+/-2)	11.9	11.3	11.7	12.5	12.3	11.8	11.5
	70-98 (+/-3)	N/A	12.5	11.6	12.3	11.7	11.0	11.5
	120 (+/-3)	N/A	N/A	11.6	11.5	10.8	10.5	11.3
	180 (+/-3)	N/A	N/A	10.8	10.0	10.7	10.7	10.6
	9 months	N/A	N/A	10.4	N/A	N/A	N/A	10.8
	12 months	N/A	N/A	10.4	N/A	N/A	N/A	10.4
	18 months	N/A	N/A	10.3	N/A	N/A	N/A	N/A

Group		1		2a/b				3
Treatment		ITFFB		AAV				ITFFB
Dose		N/A		3.0 x 10^{13} GC<br>5.0 x 10^{11} GC/g Brain				N/A
GALC Mutation Status[a]		Homozygous (Krabbe Dog)		Homozygous (Krabbe Dog)				Wild Type
	Study Day[b]	K930 (F)	K948 (M)	K933 (M)	K937 (M)	K938 (M)	K939 (F)	K928 (F)
	19 months	N/A	N/A	N/A	N/A	N/A	N/A	9.4
Sodium (mEq/L)	0	142	145	138	140	145	141	142
	14 (+/-1)	147	144	144	148	144	146	152
	28 (+/-2)	144	146	145	146	145	142	143
	70-98 (+/-3)	N/A	156	147	146	149	147	145
	120 (+/-3)	N/A	N/A	144	151	151	146	145
	180 (+/-3)	N/A	N/A	147	149	148	147	147
	9 months	N/A	N/A	150	150[a]	N/A	N/A	154
	12 months	N/A	N/A	151	N/A	N/A	N/A	153
	18 months	N/A	N/A	155	N/A	N/A	N/A	150
	19 months	N/A	N/A	143[a]	N/A	N/A	N/A	149
Potassium (mEq/L)	0	4.5	4.9	5.0	5.0	5.3	4.2	5.1
	14 (+/-1)	5.6	5.6	6.6	5.8	5.0	4.8	6.0
	28 (+/-2)	5.2	4.9	4.5	4.3	4.8	4.2	4.9
	70-98 (+/-3)	N/A	4.8	4.3	5.1	5.3	4.9	4.6
	120 (+/-3)	N/A	N/A	4.3	4.8	4.6	4.1	4.3
	180 (+/-3)	N/A	N/A	4.3	4.2	4.4	4.3	4.3
	9 months	N/A	N/A	4.2	4.2[c]	N/A	N/A	4.2
	12 months	N/A	N/A	4.8	N/A	N/A	N/A	4.5
	18 months	N/A	N/A	4.1	N/A	N/A	N/A	4
	19 months	N/A	N/A	4.1[c]	N/A	N/A	N/A	3.7

Group		1		2a/b				3
Treatment		ITFFB		AAV				ITFFB
Dose		N/A		3.0×10^{13} GC<br>5.0×10^{11} GC/g Brain				N/A
GALC Mutation Status[a]		Homozygous (Krabbe Dog)		Homozygous (Krabbe Dog)				Wild Type
	Study Day[b]	K930 (F)	K948 (M)	K933 (M)	K937 (M)	K938 (M)	K939 (F)	K928 (F)
Na/K Ratio	0	32	30	28	28	27	34	28
	14 (+/-1)	26	26	22	26	29	30	25
	28 (+/-2)	28	30	32	34	30	34	29
	70-98 (+/-3)	N/A	33	34	29	28	30	32
	120 (+/-3)	N/A	N/A	33	31	33	36	34
	180 (+/-3)	N/A	N/A	34	35	34	34	34
	9 months	N/A	N/A	36	36[c]	N/A	N/A	37
	12 months	N/A	N/A	31	N/A	N/A	N/A	34
	18 months	N/A	N/A	38	N/A	N/A	N/A	38
	19 months	N/A	N/A	35[c]	N/A	N/A	N/A	40
Chloride (mEq/L)	0	100	104	101	101	103	104	101
	14 (+/-1)	108	106	108	108	105	105	111
	28 (+/-2)	107	106	110	109	107	108	108
	70-98 (+/-3)	N/A	114	110	110	109	111	111
	120 (+/-3)	N/A	N/A	108	110	110	110	108
	180 (+/-3)	N/A	N/A	111	114	111	110	109
	9 months	N/A	N/A	114	127[c]	N/A	N/A	118
	12 months	N/A	N/A	116	N/A	N/A	N/A	117
	18 months	N/A	N/A	118	N/A	N/A	N/A	112
	19 months	N/A	N/A	97[c]	N/A	N/A	N/A	114

Group		1		2a/b				3
Treatment		ITFFB		AAV				ITFFB
Dose		N/A		3.0×10^{13} GC<br>5.0×10^{11} GC/g Brain				N/A
GALC Mutation Status[a]		Homozygous<br>(Krabbe Dog)		Homozygous<br>(Krabbe Dog)				Wild Type
	Study Day[b]	K930 (F)	K948 (M)	K933 (M)	K937 (M)	K938 (M)	K939 (F)	K928 (F)
Cholesterol (mg/dL)	0	215	264	237	218	216	187	241
	14 (+/-1)	261	318	256	233	252	197	229
	28 (+/-2)	160	194	188	143	154	176	126
	70-98 (+/-3)	N/A	201	216	183	181	163	182
	120 (+/-3)	N/A	N/A	200	213	222	161	206
	180 (+/-3)	N/A	N/A	191	209	210	172	182
	9 months	N/A	N/A	173	242[c]	N/A	N/A	162
	12 months	N/A	N/A	189	N/A	N/A	N/A	173
	18 months	N/A	N/A	161	N/A	N/A	N/A	191
	19 months	N/A	N/A	217[c]	N/A	N/A	N/A	184
CPK (IU/L)	0	256	219	282	223	341	180	353
	14 (+/-1)	210	263	266	98	629	223	222
	28 (+/-2)	164	163	253	195	255	146	175
	70-98 (+/-3)	N/A	171	300	144	194	149	235
	120 (+/-3)	N/A	N/A	216	145	145	104	166
	180 (+/-3)	N/A	N/A	112	73	84	78	103
	9 months	N/A	N/A	83	N/A	N/A	N/A	162
	12 months	N/A	N/A	76	N/A	N/A	N/A	74
	18 months	N/A	N/A	58	N/A	N/A	N/A	96
	19 months	N/A	N/A	506[c]	N/A	N/A	N/A	71

K933, K928 D70 collected late on D98 instead of D70
K948 D70 collected on D66, unscheduled necropsy

Group		1		2a/b				3
Treatment		ITFFB		AAV				ITFFB
Dose		N/A		3.0×10^{13} GC<br>5.0×10^{11} GC/g Brain				N/A
GALC Mutation Status[a]		Homozygous (Krabbe Dog)		Homozygous (Krabbe Dog)				Wild Type
	Study Day[b]	K930 (F)	K948 (M)	K933 (M)	K937 (M)	K938 (M)	K939 (F)	K928 (F)

[a]Mutation status refers to the canine *GALC* A.C 473 (Y158S) loss-of-function mutation. Animals homozygous for the *GALC* loss-of-function mutation exhibit phenotypes similar to that of human Krabbe disease and are referred to as Krabbe dogs.
[b] Study day is defined as days/weeks post treatment.
[c] Unscheduled terminal bleed.

CLINICAL PATHOLOGY – BLOOD: COAGULATION PANEL

Group		1		2a/b				3
Treatment		ITFFB		AAV				ITFFB
Dose		N/A		3.0×10^{13} GC<br>5.0×10^{11} GC/g Brain				N/A
GALC Mutation Status[a]		Homozygous (Krabbe Dog)		Homozygous (Krabbe Dog)				Wild Type
	Study Day[b]	K930 (F)	K948 (M)	K933 (M)	K937 (M)	K938 (M)	K939 (F)	K928 (F)
PT (sec)	0	7.0	6.1	7.2	6.8	6.6	5.2	7.3
	14 (+/-1)	6.1	6.1	6.0	6.8	6.1	6.0	6.1
	28 (+/-2)	6.7	6.6	6.2	N/A	N/A	6.2	7.0
	70-98 (+/-3)	N/A	6.0	7.4	6.4	6.7	6.0	7.6
	120 (+/-3)	N/A	N/A	7.8	6.6	6.3	5.5	7.3
	180 (+/-3)	N/A	N/A	7.5	6.3	6.3	6.5	7.4
	9 months	N/A	N/A	6.7	6.1*	N/A	N/A	5.5
	12 months	N/A	N/A	7.4	N/A	N/A	N/A	7.6
	18 months	N/A	N/A	7.9	N/A	N/A	N/A	7.3
	19 months	N/A	N/A	N/A	N/A	N/A	N/A	9.2
APTT (sec)	0	10.0	12.2	11.4	13.2	13.1	13.4	10.5
	14 (+/-1)	13.4	10.8	10.0	11.7	11.0	12.3	11.2
	28 (+/-2)	10.5	11.1	10.6	N/A	N/A	11.1	11.0
	70-98 (+/-3)	N/A	10.3	8.9	10.0	10.3	12.1	10.2
	120 (+/-3)	N/A	N/A	10.0	8.0	8.2	9.2	10.0
	180 (+/-3)	N/A	N/A	10.0	10.7	10.0	10.0	10.0
	9 months	N/A	N/A	9.6	10.8*	N/A	N/A	9.5
	12 months	N/A	N/A	14.1	N/A	N/A	N/A	11.6
	18 months	N/A	N/A	8.9	N/A	N/A	N/A	9.1
	19 months	N/A	N/A	N/A	N/A	N/A	N/A	9.9
Fibrinogen (mg/dL)	0	171	182	117	164	181	161	158
	14 (+/-1)	193	173	170	217	162	217	163

Group		1		2a/b				3
Treatment		ITFFB		AAV				ITFFB
Dose		N/A		3.0×10^{13} GC<br>5.0×10^{11} GC/g Brain				N/A
GALC Mutation Status[a]		Homozygous<br>(Krabbe Dog)		Homozygous<br>(Krabbe Dog)				Wild Type
	Study Day[b]	K930 (F)	K948 (M)	K933 (M)	K937 (M)	K938 (M)	K939 (F)	K928 (F)
	28 (+/-2)	194	198	183	N/A	N/A	170.2	170
	70-98 (+/-3)	N/A	310	191	379	350	322	162
	120 (+/-3)	N/A	N/A	271	247	316	193	294
	180 (+/-3)	N/A	N/A	154	316	167	172	158
	9 months	N/A	N/A	178	455*	N/A	N/A	190
	12 months	N/A	N/A	188	N/A	N/A	N/A	166
	18 months	N/A	N/A	222	N/A	N/A	N/A	<45
	19 months	N/A	N/A	N/A	N/A	N/A	N/A	103
D-Dimer (ng/mL)	0	40	29	801	100	72	123	840
	14 (+/-1)	133	97	N/A	40	77	88	N/A
	28 (+/-2)	16	54	43	N/A	N/A	37	42
	70-98 (+/-3)	N/A	314	88	71	49	62	117
	120 (+/-3)	N/A	N/A	150	97	16	120	138
	180 (+/-3)	N/A	N/A	118	16	87	48	135
	9 months	N/A	N/A	158	>5400*	N/A	N/A	45
	12 months	N/A	N/A	185	N/A	N/A	N/A	58
	18 months	N/A	N/A	62	N/A	N/A	N/A	1021
	19 months	N/A	N/A	N/A	N/A	N/A	N/A	11556

K933, K928 D70 collected late on D98 instead of D70
K948 D70 collected on D66, unscheduled necropsy
[a]Mutation status refers to the canine *GALC* A.C 473 (Y158S) loss-of-function mutation. Animals homozygous for the *GALC* loss-of-function mutation exhibit phenotypes similar to that of human Krabbe disease and are referred to as Krabbe dogs.
[b] Study day is defined as days/weeks post treatment.

Group		1		2a/b				3
**Treatment**		**ITFFB**		**AAV**				**ITFFB**
**Dose**		**N/A**		**3.0 x 10^{13} GC<br>5.0 x 10^{11} GC/g Brain**				**N/A**
***GALC* Mutation Status[a]**		**Homozygous<br>(Krabbe Dog)**		**Homozygous<br>(Krabbe Dog)**				**Wild Type**
	**Study Day[b]**	**K930 (F)**	**K948 (M)**	**K933 (M)**	**K937 (M)**	**K938 (M)**	**K939 (F)**	**K928 (F)**

[c] Unscheduled terminal bleed.

CLINICAL PATHOLOGY – CEREBROSPINAL FLUID

Group		1		2a/b				3
**Treatment**		**ITFFB**		**AAV**				**ITFFB**
**Dose**		**N/A**		**3.0 x 10^{13} GC**<br>**5.0 x 10^{11} GC/g Brain**				**N/A**
***GALC* Mutation Status**[a]		**Homozygous (Krabbe Dog)**		**Homozygous (Krabbe Dog)**				**Wild Type**
	**Study Day**[b]	**K930 (F)**	**K948 (M)**	**K933 (M)**	**K937 (M)**	**K938 (M)**	**K939 (F)**	**K928 (F)**
**Color**	0	N/A	N/A	N/A	N/A	N/A	N/A	N/A
	14 (+/-1)	N/A	N/A	N/A	N/A	N/A	N/A	N/A
	28 (+/-2)	Colorless	Colorless	Colorless	Colorless	Colorless	Colorless	Colorless
	70-98 (+/-3)	N/A	Colorless	Colorless	Colorless	Colorless	Colorless	Colorless
	120 (+/-3)	N/A	N/A	Colorless	Colorless	Colorless	Colorless	Colorless
	180 (+/-3)	N/A	N/A	Colorless	Colorless	Colorless	Colorless	Colorless
	9 months	N/A	N/A	Colorless	Colorless[c]	N/A	N/A	Colorless
	12 months	N/A	N/A	Colorless	N/A	N/A	N/A	Colorless
	18 months	N/A	N/A	Colorless	N/A	N/A	N/A	Colorless
	19 months	N/A	N/A	Colorless[c]	N/A	N/A	N/A	Colorless
**Clarity**	0	N/A	N/A	N/A	N/A	N/A	N/A	N/A
	14 (+/-1)	N/A	N/A	N/A	N/A	N/A	N/A	N/A
	28 (+/-2)	Clear	Clear	Clear	Clear	Clear	Clear	Clear
	70-98 (+/-3)	N/A	Clear	Clear	Clear	Clear	Clear	Clear
	120 (+/-3)	N/A	N/A	Clear	Clear	Clear	Clear	Clear
	180 (+/-3)	N/A	N/A	Clear	Clear	Clear	Clear	Clear
	9 months	N/A	N/A	Clear	Clear[c]	N/A	N/A	Clear
	12 months	N/A	N/A	Clear	N/A	N/A	N/A	Clear
	18 months	N/A	N/A	Clear	N/A	N/A	N/A	Clear
	19 months	N/A	N/A	Clear[c]	N/A	N/A	N/A	Clear
**WBC (/µL)**	0	N/A	N/A	N/A	N/A	N/A	N/A	N/A
	14 (+/-1)	N/A	N/A	N/A	N/A	N/A	N/A	N/A

Group		1		2a/b				3
Treatment		ITFFB		AAV				ITFFB
Dose		N/A		3.0×10^{13} GC<br>5.0×10^{11} GC/g Brain				N/A
GALC Mutation Status[a]		Homozygous (Krabbe Dog)		Homozygous (Krabbe Dog)				Wild Type
	Study Day[b]	K930 (F)	K948 (M)	K933 (M)	K937 (M)	K938 (M)	K939 (F)	K928 (F)
	28 (+/-2)	0	2	10	0	6	1	0
	70-98 (+/-3)	N/A	1	2	0	0	0	0
	120 (+/-3)	N/A	N/A	1	1	3	3	1
	180 (+/-3)	N/A	N/A	2	1	1	2	0
	9 months	N/A	N/A	1	2[c]	N/A	N/A	0
	12 months	N/A	N/A	2	N/A	N/A	N/A	1
	18 months	N/A	N/A	1	N/A	N/A	N/A	0
	19 months	N/A	N/A	1[c]	N/A	N/A	N/A	0
RBC (/µL)	0	N/A	N/A	N/A	N/A	N/A	N/A	N/A
	14 (+/-1)	N/A	N/A	N/A	N/A	N/A	N/A	N/A
	28 (+/-2)	0	0	0	0	14	0	0
	70-98 (+/-3)	N/A	0	144	0	0	0	2
	120 (+/-3)	N/A	N/A	320	0	0	0	10
	180 (+/-3)	N/A	N/A	20	1	1	270	10
	9 months	N/A	N/A	1	1[c]	N/A	N/A	0
	12 months	N/A	N/A	1	N/A	N/A	N/A	10
	18 months			10	N/A	N/A	N/A	0
	19 months	N/A	N/A	10[c]	N/A	N/A	N/A	0
Total Protein (mg/dL)	0	N/A	N/A	N/A	N/A	N/A	N/A	N/A
	14 (+/-1)	N/A	N/A	N/A	N/A	N/A	N/A	N/A
	28 (+/-2)	20	29	17	N/A	N/A	N/A	10

Group		1		2a/b				3
Treatment		ITFFB		AAV				ITFFB
Dose		N/A		3.0×10^{13} GC<br>5.0×10^{11} GC/g Brain				N/A
GALC Mutation Status[a]		Homozygous (Krabbe Dog)		Homozygous (Krabbe Dog)				Wild Type
	Study Day[b]	K930 (F)	K948 (M)	K933 (M)	K937 (M)	K938 (M)	K939 (F)	K928 (F)
	70-98 (+/-3)	N/A	45	16	13	14	10	11
	120 (+/-3)	N/A	N/A	15	15	13	11	11
	180 (+/-3)	N/A	N/A	17	18	15	15	13
	9 months	N/A	N/A	17	69[c]	N/A	N/A	12
	12 months	N/A	N/A	20	N/A	N/A	N/A	11
	18 months	N/A	N/A	19	N/A	N/A	N/A	14
	19 months	N/A	N/A	19[c]	N/A	N/A	N/A	11
**Glucose (mg/dL)**	0	N/A	N/A	N/A	N/A	N/A	N/A	N/A
	14 (+/-1)	N/A	N/A	N/A	N/A	N/A	N/A	N/A
	28 (+/-2)	81	87	100	N/A	N/A	N/A	75
	70-98 (+/-3)	N/A	112	101	95	92	84	78
	120 (+/-3)	N/A	N/A	91	88	90	97	79
	180 (+/-3)	N/A	N/A	91	89	83	92	80
	9 months	N/A	N/A	83	N/A	N/A	N/A	77
	12 months	N/A	N/A	79	N/A	N/A	N/A	79
	18 months	N/A	N/A	85	N/A	N/A	N/A	87
	19 months	N/A	N/A	88[c]	N/A	N/A	N/A	56

K933, K928 D70 collected late on D98 instead of D70
K948 D70 collected on D66, unscheduled necropsy
[a]Mutation status refers to the canine *GALC* A.C 473 (Y158S) loss-of-function mutation. Animals homozygous for the *GALC* loss-of-function mutation exhibit phenotypes similar to that of human Krabbe disease and are referred to as Krabbe dogs.
[b]Study day is defined as days/weeks post treatment.
[c]Unscheduled terminal bleed.
