# Supplementary material for: Efficacy and Safety of a Krabbe Disease Gene Therapy
Source: Hum Gene Ther. 2022 May 16;33(9-10):499–517. doi: 10.1089/hum.2021.245 (PMC9142772; doi:10.1089/hum.2021.245)
Supplement: Supplemental data [file Supp_DataS2.pdf]

BLOOD

CBC & COAGULATION PANELS		SERUM CHEMISTRY PANEL		OTHER	
**Abbreviation**	**Definition**	**Abbreviation**	**Definition**	**Abbreviation**	**Definition**
ANIS	Anisocytosis	A/G	Albumin/Globulin Ratio	BL	Baseline (Day –28 to –1)
APTT	Activated PTT	ALB	Albumin	GC	Genome Copies
BAS	Absolute Basophils	ALD	Aldolase	HEM	Hemolyzed
BAS%	% Basophils	ALP	Alkaline Phosphatase	ID	Identification Number
CBC	Complete Blood Count	ALT	Alanine Aminotransferase	ITFFB	Intrathecal Final Formulation Buffer
CPLT	Clumped Platelets	AST	Aspartate Aminotransferase	N/A	Not Applicable
DIME	D-DIMER-FEU	BUN	Blood Urea Nitrogen		
EOS	Absolute Eosinophils	CA	Calcium		
EOS%	% Eosinophils	CKBB	CPK, Brain Isoenzyme		
FDP	Fibrin Degradation Products	CKB%	% CPK, Brain Isoenzyme		
FIB	Fibrinogen	CKMB	CPK, Heart Isoenzyme		
HB	Hemoglobin	CMB%	% CPK, Heart Isoenzyme		
HCT	Hematocrit	CKMM	CPK, Skeletal Muscle Isoenzyme		
LUC	Absolute Large Unstained Cells	CKM%	% CPK, Skeletal Muscle Isoenzyme		
LUC%	% Large Unstained Cells	CL	Chloride		
LYMPH	Absolute Lymphocytes	CPK	Creatine Phosphokinase		
LYMPH%	% Lymphocytes	CREA	Creatinine		
MAC	Macrocytosis	GGT	Gamma-Glutamyl Transferase		
MCH	Mean Corpuscular Hemoglobin	GLOB	Globulin (calculated as TPRO minus ALB)		
MCHC	MCH Concentration	GLU	Glucose		
MCV	Mean Corpuscular Volume	K	Potassium		
MONO	Absolute Monocytes	LDH	Lactate Dehydrogenase		
MONO%	% Monocytes	NA	Sodium		
NEU	Absolute Neutrophils	PHOS	Inorganic Phosphorus		
NEU%	% Neutrophils	QNS	Quantity Not Sufficient		
NOSA	No Sample Submitted	TBIL	Total Bilirubin		
PLT	Platelet Count	TPRO	Total Protein		
PT	Prothrombin Time				
RBC	Red Blood Cell Count				
TRB	Turbid				
WBC	White Blood Cell Count				

Clinical Pathology - Blood - Page 2
Clinical Pathology - Cerebrospinal Fluid - Page 42

	Group (Necropsy Day)	Group 1 (Necropsy Day 90±4)									
	Animal ID (Sex)	18-162 (Female)									
	Treatment (Dose)	ITFFB (N/A)									
	Study Day	BL	0	7±1	14±2	28±3	60±3	90±4	120±4	150±4	180±5
Complete Blood Counts (Hematology) & Plasma Coagulation	WBC (10^3/µL)	3.93	5.80	2.91	7.22	6.00	11.98	5.09	N/A	N/A	N/A
	RBC (10^6/µL)	5.77	5.48	5.51	5.75	5.82	6.20	5.78	N/A	N/A	N/A
	HB (g/dL)	13.6	13.0	13.0	13.2	13.3	14.5	13.4	N/A	N/A	N/A
	HCT (%)	45.2	43.0	42.9	44.8	44.9	47.1	43.5	N/A	N/A	N/A
	MCV (fL)	78.3	78.5	77.7	77.9	77.0	76.0	75.3	N/A	N/A	N/A
	MCH (pg)	23.6	23.8	23.5	22.9	22.9	23.3	23.2	N/A	N/A	N/A
	MCHC (g/dL)	30.1	30.3	30.3	29.4	29.7	30.7	30.8	N/A	N/A	N/A
	PLT (10^3/µL)	101	423	231	301	392	417	351	N/A	N/A	N/A
	NEU% (%)	35.3	35.8	33.4	33.1	43.4	73.3	41.2	N/A	N/A	N/A
	NEU (10^3/µL)	1.39	2.07	0.97	2.39	2.61	8.77	2.10	N/A	N/A	N/A
	LYMPH% (%)	56.9	57.2	57.1	59.3	49.4	20.8	49.4	N/A	N/A	N/A
	LYMPH (10^3/µL)	2.23	3.31	1.66	4.28	2.96	2.50	2.52	N/A	N/A	N/A
	MONO% (%)	3.2	2.8	5.6	3.4	3.2	3.7	4.0	N/A	N/A	N/A
	MONO (10^3/µL)	0.12	0.16	0.16	0.25	0.19	0.44	0.20	N/A	N/A	N/A
	EOS% (%)	3.0	2.6	1.1	1.9	2.1	0.6	3.0	N/A	N/A	N/A
	EOS (10^3/µL)	0.12	0.15	0.03	0.14	0.12	0.08	0.15	N/A	N/A	N/A
	BAS% (%)	0.4	0.4	0.6	1.0	0.4	0.8	0.5	N/A	N/A	N/A
	BAS (10^3/µL)	0.02	0.02	0.02	0.07	0.02	0.10	0.03	N/A	N/A	N/A
	LUC% (%)	1.2	1.2	2.1	1.3	1.6	0.8	1.9	N/A	N/A	N/A
	LUC (10^3/µL)	0.05	0.07	0.06	0.10	0.09	0.09	0.10	N/A	N/A	N/A
	MAC	+	+	+	+	+	+	+	N/A	N/A	N/A
	LPLT	N/A	N/A	N/A	N/A	N/A	N/A	N/A	N/A	N/A	N/A
	CPLT	N/A	N/A	N/A	N/A	N/A	N/A	+	N/A	N/A	N/A
	ANIS	N/A	N/A	N/A	N/A	N/A	N/A	N/A	N/A	N/A	N/A
	ATYP	N/A	N/A	N/A	N/A	N/A	N/A	N/A	N/A	N/A	N/A
	COMMENT	N/A	N/A	N/A	N/A	HEM	N/A	N/A	N/A	N/A	N/A
	PT (sec)	10.4	10.5	10.5	10.4	10.5	11.1	11.0	N/A	N/A	N/A
	APTT (sec)	21.2	21.9	21.3	21.5	22.0	23.6	22.7	N/A	N/A	N/A
	FIB (mg/dL)	179	195	183	176	199	191	181	N/A	N/A	N/A
	DIME (ng/mL)	449.4	706.0	470.8	380.7	282.7	830.1	458.0	N/A	N/A	N/A
	FDP (FEU)	<2.5	5.0	<2.5	2.5	<2.5	2.5	2.5	N/A	N/A	N/A

	Group (Necropsy Day)	Group 1 (Necropsy Day 90±4)									
	Animal ID (Sex)	18-162 (Female)									
	Treatment (Dose)	ITFFB (N/A)									
	Study Day	BL	0	7±1	14±2	28±3	60±3	90±4	120±4	150±4	180±5
Serum Chemistry	BUN (mg/dL)	19	25	29	17	30	37	28	N/A	N/A	N/A
	CREA (mg/dL)	0.4	0.5	0.4	0.6	0.5	0.4	0.4	N/A	N/A	N/A
	GLU (mg/dL)	126	95	69	100	62	48	93	N/A	N/A	N/A
	NA (mmol/L)	147	148	150	148	147	147	147	N/A	N/A	N/A
	K (mmol/L)	4.5	3.7	3.8	3.3	3.7	3.7	3.5	N/A	N/A	N/A
	CL (mmol/L)	113	113	114	109	110	110	109	N/A	N/A	N/A
	ALP (U/L)	796	746	640	757	639	859	734	N/A	N/A	N/A
	ALT (U/L)	28	24	25	27	28	30	25	N/A	N/A	N/A
	AST (U/L)	46	21	21	24	33	32	20	N/A	N/A	N/A
	TBIL (mg/dL)	0.2	0.1	0.1	0.1	0.1	0.2	0.1	N/A	N/A	N/A
	LDH (U/L)	614	202	234	240	511	423	213	N/A	N/A	N/A
	CPK (U/L)	1299	136	207	177	192	379	184	N/A	N/A	N/A
	GGT (U/L)	65	62	61	58	65	71	61	N/A	N/A	N/A
	TPRO (g/dL)	5.9	5.6	5.7	5.4	5.7	5.7	5.2	N/A	N/A	N/A
	ALB (g/dL)	4.0	3.8	3.9	3.8	3.8	4.0	3.6	N/A	N/A	N/A
	GLOB (g/dL)	1.9	1.8	1.8	1.6	1.9	1.7	1.6	N/A	N/A	N/A
	A/G	2.11	2.11	2.17	2.38	2.00	2.35	2.25	N/A	N/A	N/A
	CA (mg/dL)	9.7	9.4	9.4	9.3	9.2	9.1	9.2	N/A	N/A	N/A
	PHOS (mg/dL)	6.9	6.3	7.6	6.2	6.8	7.6	6.8	N/A	N/A	N/A
	ALD (U/L)	46.6	17.7	15.5	19.0	31.5	26.0	15.4	N/A	N/A	N/A
	CKMM (U/L)	1135	54	122	82	104	282	85	N/A	N/A	N/A
	CKM% (%)	87.4	39.4	58.9	46.6	54.2	74.3	46.0	N/A	N/A	N/A
	CKMB (U/L)	99	39	45	31	26	37	47	N/A	N/A	N/A
	CMB% (%)	7.6	28.7	21.8	17.3	13.7	9.7	25.3	N/A	N/A	N/A
	CKBB (U/L)	64	43	40	64	62	61	53	N/A	N/A	N/A
	CKB% (%)	4.9	31.9	19.2	36.1	32.2	16.1	28.8	N/A	N/A	N/A
	COMMENT	HEM	N/A	N/A	N/A	N/A	N/A	N/A	N/A	N/A	N/A

	Group (Necropsy Day)	Group 5 (Necropsy Day 180±5)									
	Animal ID (Sex)	18-159 (Male)									
	Treatment (Dose)	ITFFB (N/A)									
	Study Day	BL	0	7±1	14±2	28±3	60±3	90±4	120±4	150±4	180±5
Complete Blood Counts (Hematology) & Plasma Coagulation	WBC (10^3/µL)	10.05	9.68	6.72	11.32	10.09	10.00	9.66	9.65	9.02	7.01
	RBC (10^6/µL)	5.99	5.42	5.55	5.63	5.56	5.61	5.91	5.85	5.85	5.96
	HB (g/dL)	13.9	12.8	12.7	13.0	13.1	12.9	13.6	13.3	13.3	13.7
	HCT (%)	46.4	41.9	42.9	44.1	43.1	43.2	44.5	44.1	45.4	45.8
	MCV (fL)	77.4	77.2	77.3	78.4	77.6	77.0	75.3	75.4	77.6	76.7
	MCH (pg)	23.3	23.7	22.9	23.1	23.5	22.9	23.0	22.8	22.8	23.0
	MCHC (g/dL)	30.1	30.7	29.6	29.5	30.3	29.8	30.5	30.2	29.4	30.0
	PLT (10^3/µL)	347	178	379	408	385	338	373	385	384	343
	NEU% (%)	67.5	24.8	37.2	39.7	27.3	34.9	25.8	31.5	30.0	31.9
	NEU (10^3/µL)	6.78	2.40	2.50	4.49	2.76	3.49	2.49	3.04	2.70	2.24
	LYMPH% (%)	29.2	63.8	57.2	53.7	64.7	58.3	69.0	63.5	63.0	62.7
	LYMPH (10^3/µL)	2.93	6.18	3.84	6.08	6.53	5.83	6.66	6.13	5.68	4.40
	MONO% (%)	2.1	4.5	2.6	4.2	3.4	2.4	2.0	1.9	2.8	2.2
	MONO (10^3/µL)	0.21	0.44	0.17	0.47	0.34	0.24	0.19	0.18	0.25	0.15
	EOS% (%)	0.4	2.4	1.0	0.6	1.8	2.9	1.0	1.3	2.8	1.2
	EOS (10^3/µL)	0.04	0.23	0.07	0.07	0.19	0.29	0.10	0.12	0.25	0.08
	BAS% (%)	0.2	1.0	0.6	0.4	0.7	0.5	0.6	0.5	0.5	0.6
	BAS (10^3/µL)	0.02	0.10	0.04	0.04	0.07	0.05	0.05	0.04	0.04	0.04
	LUC% (%)	0.6	3.4	1.5	1.4	2.0	1.0	1.7	1.4	1.0	1.4
	LUC (10^3/µL)	0.06	0.33	0.10	0.16	0.20	0.10	0.17	0.14	0.09	0.10
	MAC	+	+	+	+	+	+	+	+	+	+
	LPLT	N/A	N/A	N/A	N/A	N/A	N/A	N/A	N/A	N/A	N/A
	CPLT	+	N/A	+	+	+	+	N/A	N/A	N/A	N/A
	ANIS	N/A	N/A	N/A	N/A	N/A	N/A	N/A	N/A	N/A	N/A
	ATYP	N/A	N/A	N/A	N/A	N/A	N/A	N/A	N/A	N/A	N/A
	COMMENT	N/A	N/A	N/A	HEM	N/A	N/A	N/A	N/A	N/A	N/A
	PT (sec)	11.0	11.1	10.7	11.0	10.8	11.0	11.6	11.6	11.1	11.0
	APTT (sec)	21.1	21.8	20.6	21.9	21.9	22.6	23.3	23.2	22.3	21.7
	FIB (mg/dL)	179	226	206	163	189	185	177	191	216	181
	DIME (ng/mL)	271.2	289.3	221.9	1649.	303.3	321.2	356.5	343.8	302.8	259.0
	FDP (FEU)	<2.5	<2.5	<2.5	5.0	<2.5	<2.5	<2.5	<2.5	<2.5	<2.5

	Group (Necropsy Day)	Group 5 (Necropsy Day 180±5)									
	Animal ID (Sex)	18-159 (Male)									
	Treatment (Dose)	ITFFB (N/A)									
	Study Day	BL	0	7±1	14±2	28±3	60±3	90±4	120±4	150±4	180±5
Serum Chemistry	BUN (mg/dL)	27	28	21	27	22	23	20	22	25	19
	CREA (mg/dL)	0.5	0.4	0.4	0.5	0.3	0.4	0.4	0.4	0.5	0.4
	GLU (mg/dL)	84	119	106	80	134	67	85	80	93	114
	NA (mmol/L)	149	146	148	148	144	145	146	149	147	147
	K (mmol/L)	3.8	3.8	3.3	3.3	3.1	3.3	3.1	3.2	3.6	3.4
	CL (mmol/L)	113	105	110	105	106	105	107	107	105	109
	ALP (U/L)	682	651	560	756	703	912	755	703	656	643
	ALT (U/L)	31	22	21	23	22	22	22	26	23	25
	AST (U/L)	34	38	25	30	20	24	18	24	23	21
	TBIL (mg/dL)	0.2	0.2	0.2	0.2	0.1	0.2	0.2	0.2	0.2	0.2
	LDH (U/L)	325	429	268	319	239	251	222	268	299	251
	CPK (U/L)	193	1099	280	381	185	128	154	138	241	155
	GGT (U/L)	71	66	64	69	74	85	79	87	86	74
	TPRO (g/dL)	6.4	5.6	6.2	6.1	5.8	6.0	5.8	6.2	6.4	5.9
	ALB (g/dL)	4.0	3.8	3.9	3.9	3.6	3.8	3.8	4.1	4.0	3.9
	GLOB (g/dL)	2.4	1.8	2.3	2.2	2.2	2.2	2.0	2.1	2.4	2.0
	A/G	1.67	2.11	1.70	1.77	1.64	1.73	1.90	1.95	1.67	1.95
	CA (mg/dL)	9.7	10.1	10.0	10.1	9.5	9.5	9.5	9.9	10.3	9.7
	PHOS (mg/dL)	6.7	6.5	6.2	6.7	5.7	6.6	5.9	5.8	5.4	5.1
	ALD (U/L)	25.3	32.8	17.5	26.0	18.8	14.0	13.0	17.9	17.1	13.6
	CKMM (U/L)	133	928	212	279	102	57	63	83	162	97
	CKM% (%)	69.0	84.4	75.7	73.1	55.2	44.5	41.2	60.5	67.2	62.4
	CKMB (U/L)	34	75	38	36	40	27	49	23	34	29
	CMB% (%)	17.8	6.8	13.7	9.5	21.6	21.4	31.5	16.9	14.3	18.7
	CKBB (U/L)	25	97	30	66	43	44	42	31	45	29
	CKB% (%)	13.2	8.8	10.6	17.4	23.2	34.1	27.3	22.6	18.5	18.9
	COMMENT	N/A	HEM	N/A	N/A	N/A	N/A	N/A	N/A	N/A	N/A

	Group (Necropsy Day)	Group 2 (Necropsy Day 90±4)									
	Animal ID (Sex)	18-091 (Male)									
	Treatment (Dose)	GTP-206 (4.5 x 10^{12} GC/Animal – Low Dose)									
	Study Day	BL	0	7±1	14±2	28±3	60±3	90±4	120±4	150±4	180±5
Complete Blood Counts (Hematology) & Plasma Coagulation	WBC (10^3/µL)	6.60	6.93	7.84	9.01	7.08	6.98	8.16	N/A	N/A	N/A
	RBC (10^6/µL)	6.04	5.89	5.91	6.15	6.04	6.35	6.37	N/A	N/A	N/A
	HB (g/dL)	12.7	13.0	12.6	12.9	12.9	13.5	13.0	N/A	N/A	N/A
	HCT (%)	43.0	42.2	42.4	44.0	43.2	45.5	44.7	N/A	N/A	N/A
	MCV (fL)	71.3	71.6	71.7	71.6	71.5	71.7	70.2	N/A	N/A	N/A
	MCH (pg)	21.0	22.1	21.2	21.0	21.3	21.2	20.5	N/A	N/A	N/A
	MCHC (g/dL)	29.5	30.8	29.6	29.3	29.8	29.6	29.2	N/A	N/A	N/A
	PLT (10^3/µL)	407	436	364	473	306	429	375	N/A	N/A	N/A
	NEU% (%)	30.4	26.4	48.7	26.2	21.8	20.7	51.2	N/A	N/A	N/A
	NEU (10^3/µL)	2.01	1.83	3.82	2.37	1.54	1.44	4.18	N/A	N/A	N/A
	LYMPH% (%)	62.4	64.1	46.7	67.8	70.0	72.5	45.1	N/A	N/A	N/A
	LYMPH (10^3/µL)	4.12	4.44	3.66	6.11	4.95	5.06	3.69	N/A	N/A	N/A
	MONO% (%)	2.1	3.6	2.0	2.1	3.1	2.2	1.5	N/A	N/A	N/A
	MONO (10^3/µL)	0.14	0.25	0.16	0.19	0.22	0.15	0.12	N/A	N/A	N/A
	EOS% (%)	2.9	3.7	0.4	1.8	2.8	2.7	0.5	N/A	N/A	N/A
	EOS (10^3/µL)	0.19	0.26	0.03	0.16	0.20	0.19	0.04	N/A	N/A	N/A
	BAS% (%)	0.8	0.6	0.6	1.0	0.7	0.7	0.4	N/A	N/A	N/A
	BAS (10^3/µL)	0.05	0.04	0.04	0.09	0.05	0.05	0.04	N/A	N/A	N/A
	LUC% (%)	1.4	1.6	1.5	1.1	1.6	1.3	1.3	N/A	N/A	N/A
	LUC (10^3/µL)	0.09	0.11	0.12	0.10	0.11	0.09	0.10	N/A	N/A	N/A
	MAC	N/A	N/A	N/A	N/A	N/A	N/A	N/A	N/A	N/A	N/A
	LPLT	N/A	N/A	N/A	N/A	N/A	N/A	N/A	N/A	N/A	N/A
	CPLT	N/A	N/A	N/A	N/A	+	N/A	N/A	N/A	N/A	N/A
	ANIS	N/A	N/A	N/A	N/A	N/A	N/A	N/A	N/A	N/A	N/A
	ATYP	N/A	N/A	N/A	N/A	N/A	N/A	N/A	N/A	N/A	N/A
	COMMENT	N/A	N/A	HEM	N/A	N/A	N/A	N/A	N/A	N/A	N/A
	PT (sec)	11.9	11.2	10.8	10.8	10.7	11.3	11.4	N/A	N/A	N/A
	APTT (sec)	22.0	23.5	21.3	22.4	22.3	22.5	23.1	N/A	N/A	N/A
	FIB (mg/dL)	89	174	208	172	187	166	161	N/A	N/A	N/A
	DIME (ng/mL)	226.9	215.8	175.0	131.5	281.4	173.4	165.3	N/A	N/A	N/A
	FDP (FEU)	<2.5	<2.5	<2.5	<2.5	2.5	2.5	2.5	N/A	N/A	N/A

	Group (Necropsy Day)	Group 2 (Necropsy Day 90±4)									
	Animal ID (Sex)	18-091 (Male)									
	Treatment (Dose)	GTP-206 (4.5 x 10^{12} GC/Animal – Low Dose)									
	Study Day	BL	0	7±1	14±2	28±3	60±3	90±4	120±4	150±4	180±5
Serum Chemistry	BUN (mg/dL)	24	18	25	21	18	19	18	N/A	N/A	N/A
	CREA (mg/dL)	0.4	0.4	0.5	0.4	0.4	0.3	0.3	N/A	N/A	N/A
	GLU (mg/dL)	123	85	96	111	92	109	96	N/A	N/A	N/A
	NA (mmol/L)	146	144	148	147	145	145	149	N/A	N/A	N/A
	K (mmol/L)	3.4	3.4	3.6	3.1	3.3	3.3	3.4	N/A	N/A	N/A
	CL (mmol/L)	110	107	110	107	109	108	111	N/A	N/A	N/A
	ALP (U/L)	733	610	641	557	609	759	690	N/A	N/A	N/A
	ALT (U/L)	28	26	21	23	26	21	23	N/A	N/A	N/A
	AST (U/L)	26	31	24	23	25	27	26	N/A	N/A	N/A
	TBIL (mg/dL)	0.1	0.2	0.2	0.1	0.1	0.1	0.1	N/A	N/A	N/A
	LDH (U/L)	248	313	232	198	201	268	194	N/A	N/A	N/A
	CPK (U/L)	141	234	182	120	164	212	384	N/A	N/A	N/A
	GGT (U/L)	96	92	94	85	89	98	87	N/A	N/A	N/A
	TPRO (g/dL)	6.0	5.8	6.3	6.1	6.0	6.1	5.7	N/A	N/A	N/A
	ALB (g/dL)	3.9	4.0	4.0	4.0	3.8	3.9	3.7	N/A	N/A	N/A
	GLOB (g/dL)	2.1	1.8	2.3	2.1	2.2	2.2	2.0	N/A	N/A	N/A
	A/G	1.86	2.22	1.74	1.90	1.73	1.77	1.85	N/A	N/A	N/A
	CA (mg/dL)	9.5	9.7	10.0	9.8	9.2	9.4	9.3	N/A	N/A	N/A
	PHOS (mg/dL)	5.2	6.1	5.8	6.0	5.9	5.9	6.4	N/A	N/A	N/A
	ALD (U/L)	15.3	20.3	10.3	12.6	12.3	14.8	12.2	N/A	N/A	N/A
	CKMM (U/L)	95	152	116	54	92	126	297	N/A	N/A	N/A
	CKM% (%)	67.2	64.8	63.9	45.4	56.3	59.3	77.3	N/A	N/A	N/A
	CKMB (U/L)	26	33	33	32	31	40	45	N/A	N/A	N/A
	CMB% (%)	18.6	14.2	18.4	26.4	19.1	18.8	11.6	N/A	N/A	N/A
	CKBB (U/L)	20	49	32	34	41	46	43	N/A	N/A	N/A
	CKB% (%)	14.2	21.0	17.8	28.2	24.7	21.8	11.2	N/A	N/A	N/A
	COMMENT	N/A	N/A	N/A	N/A	N/A	N/A	N/A	N/A	N/A	N/A

	Group (Necropsy Day)	Group 2 (Necropsy Day 90±4)									
	Animal ID (Sex)	18-168 (Female)									
	Treatment (Dose)	GTP-206 (4.5 x 10^{12} GC/Animal – Low Dose)									
	Study Day	BL	0	7±1	14±2	28±3	60±3	90±4	120±4	150±4	180±5
Complete Blood Counts (Hematology) & Plasma Coagulation	WBC (10^3/µL)	14.36	8.47	12.23	13.30	13.33	11.64	9.96	N/A	N/A	N/A
	RBC (10^6/µL)	5.65	5.35	5.36	5.07	5.62	5.63	5.23	N/A	N/A	N/A
	HB (g/dL)	13.0	12.4	12.2	11.7	13.0	12.8	12.0	N/A	N/A	N/A
	HCT (%)	44.6	41.3	41.4	39.5	43.7	43.5	40.0	N/A	N/A	N/A
	MCV (fL)	78.9	77.1	77.2	77.8	77.7	77.3	76.4	N/A	N/A	N/A
	MCH (pg)	23.0	23.2	22.8	23.1	23.2	22.8	22.9	N/A	N/A	N/A
	MCHC (g/dL)	29.2	30.1	29.5	29.6	29.8	29.5	30.0	N/A	N/A	N/A
	PLT (10^3/µL)	402	420	360	380	360	357	313	N/A	N/A	N/A
	NEU% (%)	54.2	29.0	37.5	59.8	56.2	57.3	56.5	N/A	N/A	N/A
	NEU (10^3/µL)	7.79	2.46	4.58	7.96	7.50	6.67	5.62	N/A	N/A	N/A
	LYMPH% (%)	41.0	61.5	52.9	34.9	36.7	37.1	38.5	N/A	N/A	N/A
	LYMPH (10^3/µL)	5.89	5.21	6.47	4.65	4.90	4.32	3.83	N/A	N/A	N/A
	MONO% (%)	1.5	2.1	4.8	3.2	3.5	1.9	1.4	N/A	N/A	N/A
	MONO (10^3/µL)	0.21	0.18	0.59	0.42	0.47	0.23	0.14	N/A	N/A	N/A
	EOS% (%)	1.9	5.0	3.0	1.1	2.1	2.4	2.3	N/A	N/A	N/A
	EOS (10^3/µL)	0.28	0.42	0.37	0.15	0.28	0.28	0.22	N/A	N/A	N/A
	BAS% (%)	0.6	0.8	0.7	0.4	0.7	0.4	0.6	N/A	N/A	N/A
	BAS (10^3/µL)	0.08	0.06	0.08	0.06	0.09	0.04	0.06	N/A	N/A	N/A
	LUC% (%)	0.8	1.6	1.1	0.5	0.8	0.9	0.8	N/A	N/A	N/A
	LUC (10^3/µL)	0.12	0.14	0.14	0.07	0.10	0.10	0.08	N/A	N/A	N/A
	MAC	+	+	+	+	+	+	+	N/A	N/A	N/A
	LPLT	N/A	N/A	N/A	N/A	N/A	N/A	N/A	N/A	N/A	N/A
	CPLT	N/A	N/A	+	+	+	N/A	+	N/A	N/A	N/A
	ANIS	N/A	N/A	N/A	N/A	N/A	N/A	N/A	N/A	N/A	N/A
	ATYP	N/A	N/A	N/A	N/A	N/A	N/A	N/A	N/A	N/A	N/A
	COMMENT	N/A	N/A	HEM	N/A	N/A	N/A	N/A	N/A	N/A	N/A
	PT (sec)	10.8	11.1	10.9	10.9	10.5	11.1	11.4	N/A	N/A	N/A
	APTT (sec)	20.9	21.5	21.7	21.2	20.9	21.5	21.4	N/A	N/A	N/A
	FIB (mg/dL)	172	179	140	157	189	172	158	N/A	N/A	N/A
	DIME (ng/mL)	259.8	188.4	279.7	168.8	243.3	189.7	156.4	N/A	N/A	N/A
	FDP (FEU)	<2.5	<2.5	<2.5	<2.5	<2.5	<2.5	<2.5	N/A	N/A	N/A

	Group (Necropsy Day)	Group 2 (Necropsy Day 90±4)									
	Animal ID (Sex)	18-168 (Female)									
	Treatment (Dose)	GTP-206 (4.5 x 10^{12} GC/Animal – Low Dose)									
	Study Day	BL	0	7±1	14±2	28±3	60±3	90±4	120±4	150±4	180±5
Serum Chemistry	BUN (mg/dL)	26	22	19	20	23	23	20	N/A	N/A	N/A
	CREA (mg/dL)	0.5	0.4	0.4	0.5	0.4	0.4	0.4	N/A	N/A	N/A
	GLU (mg/dL)	91	81	66	65	65	57	97	N/A	N/A	N/A
	NA (mmol/L)	149	148	147	150	147	147	149	N/A	N/A	N/A
	K (mmol/L)	3.4	3.7	3.6	3.3	3.7	3.4	3.8	N/A	N/A	N/A
	CL (mmol/L)	107	109	110	109	109	109	111	N/A	N/A	N/A
	ALP (U/L)	584	604	537	661	579	602	522	N/A	N/A	N/A
	ALT (U/L)	34	29	23	31	35	30	28	N/A	N/A	N/A
	AST (U/L)	31	33	37	33	31	29	27	N/A	N/A	N/A
	TBIL (mg/dL)	0.1	0.1	0.2	0.2	0.2	0.2	0.1	N/A	N/A	N/A
	LDH (U/L)	216	238	328	214	222	185	217	N/A	N/A	N/A
	CPK (U/L)	110	833	1924	142	124	146	236	N/A	N/A	N/A
	GGT (U/L)	54	53	51	48	52	51	44	N/A	N/A	N/A
	TPRO (g/dL)	6.4	5.7	6.5	6.3	6.5	6.4	5.5	N/A	N/A	N/A
	ALB (g/dL)	4.1	4.1	4.1	4.3	4.1	4.1	3.6	N/A	N/A	N/A
	GLOB (g/dL)	2.3	1.6	2.4	2.0	2.4	2.3	1.9	N/A	N/A	N/A
	A/G	1.78	2.56	1.71	2.15	1.71	1.78	1.89	N/A	N/A	N/A
	CA (mg/dL)	10.1	9.9	9.6	9.8	9.8	9.4	9.1	N/A	N/A	N/A
	PHOS (mg/dL)	6.0	6.9	7.2	6.9	5.8	5.1	6.4	N/A	N/A	N/A
	ALD (U/L)	13.7	20.2	35.1	17.0	15.2	13.3	13.0	N/A	N/A	N/A
	CKMM (U/L)	63	694	1716	78	68	85	141	N/A	N/A	N/A
	CKM% (%)	57.2	83.3	89.2	55.2	55.0	58.3	59.6	N/A	N/A	N/A
	CKMB (U/L)	24	67	100	35	28	32	47	N/A	N/A	N/A
	CMB% (%)	22.1	8.1	5.2	24.7	22.6	22.1	19.8	N/A	N/A	N/A
	CKBB (U/L)	23	72	108	29	28	29	49	N/A	N/A	N/A
	CKB% (%)	20.8	8.6	5.6	20.1	22.3	19.6	20.6	N/A	N/A	N/A
	COMMENT	N/A	N/A	N/A	N/A	N/A	N/A	N/A	N/A	N/A	N/A

	Group (Necropsy Day)	Group 2 (Necropsy Day 90±4)									
	Animal ID (Sex)	18-173 (Female)									
	Treatment (Dose)	GTP-206 (4.5 x 10^{12} GC/Animal – Low Dose)									
	Study Day	BL	0	7±1	14±2	28±3	60±3	90±4	120±4	150±4	180±5
Complete Blood Counts (Hematology) & Plasma Coagulation	WBC (10^3/µL)	19.06	12.28	8.66	14.99	13.72	8.44	CLOT	N/A	N/A	N/A
	RBC (10^6/µL)	5.42	5.07	5.37	5.08	5.30	5.45	CLOT	N/A	N/A	N/A
	HB (g/dL)	12.5	11.9	12.2	11.6	12.1	12.1	CLOT	N/A	N/A	N/A
	HCT (%)	41.5	38.5	41.3	39.1	39.9	41.1	CLOT	N/A	N/A	N/A
	MCV (fL)	76.6	76.0	76.9	76.9	75.3	75.4	CLOT	N/A	N/A	N/A
	MCH (pg)	23.1	23.5	22.6	22.8	22.9	22.2	CLOT	N/A	N/A	N/A
	MCHC (g/dL)	30.2	30.9	29.4	29.6	30.4	29.4	CLOT	N/A	N/A	N/A
	PLT (10^3/µL)	371	415	479	386	241	345	CLOT	N/A	N/A	N/A
	NEU% (%)	78.6	66.9	39.4	80.3	73.3	46.3	CLOT	N/A	N/A	N/A
	NEU (10^3/µL)	14.99	8.22	3.41	12.03	10.06	3.91	CLOT	N/A	N/A	N/A
	LYMPH% (%)	16.9	27.4	54.5	16.1	22.2	48.0	CLOT	N/A	N/A	N/A
	LYMPH (10^3/µL)	3.23	3.37	4.72	2.41	3.04	4.05	CLOT	N/A	N/A	N/A
	MONO% (%)	2.6	1.8	3.0	2.4	2.0	2.1	CLOT	N/A	N/A	N/A
	MONO (10^3/µL)	0.49	0.22	0.26	0.37	0.27	0.18	CLOT	N/A	N/A	N/A
	EOS% (%)	0.9	2.6	1.1	0.3	0.8	2.2	CLOT	N/A	N/A	N/A
	EOS (10^3/µL)	0.16	0.32	0.10	0.04	0.11	0.19	CLOT	N/A	N/A	N/A
	BAS% (%)	0.4	0.5	0.7	0.3	0.9	0.3	CLOT	N/A	N/A	N/A
	BAS (10^3/µL)	0.08	0.06	0.06	0.05	0.12	0.03	CLOT	N/A	N/A	N/A
	LUC% (%)	0.5	0.8	1.3	0.6	0.8	1.0	CLOT	N/A	N/A	N/A
	LUC (10^3/µL)	0.10	0.09	0.11	0.10	0.11	0.09	CLOT	N/A	N/A	N/A
	MAC	+	+	+	+	+	+	N/A	N/A	N/A	N/A
	LPLT	N/A	N/A	N/A	N/A	N/A	N/A	N/A	N/A	N/A	N/A
	CPLT	+	N/A	N/A	+	+	+	N/A	N/A	N/A	N/A
	ANIS	N/A	N/A	N/A	N/A	N/A	N/A	N/A	N/A	N/A	N/A
	ATYP	N/A	N/A	N/A	N/A	N/A	N/A	N/A	N/A	N/A	N/A
	COMMENT	N/A	N/A	N/A	N/A	HEM	N/A	HEM	N/A	N/A	N/A
	PT (sec)	10.3	10.7	10.2	10.3	10.6	10.7	10.7	N/A	N/A	N/A
	APTT (sec)	19.3	19.6	19.7	19.7	19.2	20.5	20.6	N/A	N/A	N/A
	FIB (mg/dL)	169	189	199	174	130	167	163	N/A	N/A	N/A
	DIME (ng/mL)	356.3	334.2	390.5	449.5	3177.2	459.8	467.3	N/A	N/A	N/A
	FDP (FEU)	<2.5	<2.5	<2.5	<2.5	10.0	<2.5	<2.5	N/A	N/A	N/A

	Group (Necropsy Day)	Group 2 (Necropsy Day 90±4)									
	Animal ID (Sex)	18-173 (Female)									
	Treatment (Dose)	GTP-206 (4.5 x 10^{12} GC/Animal – Low Dose)									
	Study Day	BL	0	7±1	14±2	28±3	60±3	90±4	120±4	150±4	180±5
Serum Chemistry	BUN (mg/dL)	24	24	22	20	21	19	15	N/A	N/A	N/A
	CREA (mg/dL)	0.4	0.5	0.4	0.5	0.4	0.4	0.3	N/A	N/A	N/A
	GLU (mg/dL)	116	105	104	94	89	100	97	N/A	N/A	N/A
	NA (mmol/L)	147	147	147	150	146	146	149	N/A	N/A	N/A
	K (mmol/L)	3.3	2.9	3.3	2.9	3.1	3.2	3.3	N/A	N/A	N/A
	CL (mmol/L)	108	106	107	109	107	109	110	N/A	N/A	N/A
	ALP (U/L)	632	681	636	697	580	737	758	N/A	N/A	N/A
	ALT (U/L)	26	25	27	27	23	22	23	N/A	N/A	N/A
	AST (U/L)	28	28	27	28	26	27	29	N/A	N/A	N/A
	TBIL (mg/dL)	0.1	0.2	0.2	0.2	0.2	0.2	0.2	N/A	N/A	N/A
	LDH (U/L)	312	248	251	241	259	258	285	N/A	N/A	N/A
	CPK (U/L)	195	197	285	135	74	307	623	N/A	N/A	N/A
	GGT (U/L)	93	97	96	90	89	106	101	N/A	N/A	N/A
	TPRO (g/dL)	6.3	5.7	6.5	6.4	6.2	6.1	5.9	N/A	N/A	N/A
	ALB (g/dL)	3.8	3.8	3.9	4.0	3.7	3.8	3.7	N/A	N/A	N/A
	GLOB (g/dL)	2.5	1.9	2.6	2.4	2.5	2.3	2.2	N/A	N/A	N/A
	A/G	1.52	2.00	1.50	1.67	1.48	1.65	1.68	N/A	N/A	N/A
	CA (mg/dL)	9.9	9.4	9.6	9.5	9.4	9.2	9.1	N/A	N/A	N/A
	PHOS (mg/dL)	4.8	6.3	7.3	6.0	5.3	5.1	7.5	N/A	N/A	N/A
	ALD (U/L)	24.2	23.1	18.9	20.9	22.8	19.3	21.0	N/A	N/A	N/A
	CKMM (U/L)	149	133	199	76	36	227	483	N/A	N/A	N/A
	CKM% (%)	76.5	67.7	69.7	56.6	48.8	73.9	77.6	N/A	N/A	N/A
	CKMB (U/L)	29	35	44	33	22	42	72	N/A	N/A	N/A
	CMB% (%)	14.7	17.8	15.3	24.5	29.3	13.6	11.5	N/A	N/A	N/A
	CKBB (U/L)	17	29	42	26	16	38	67	N/A	N/A	N/A
	CKB% (%)	8.8	14.5	14.9	19.0	21.9	12.5	10.8	N/A	N/A	N/A
	COMMENT	N/A	N/A	N/A	N/A	N/A	N/A	N/A	N/A	N/A	N/A

	Group (Necropsy Day)	Group 6 (Necropsy Day 180±5)									
	Animal ID (Sex)	18-042 (Female)									
	Treatment (Dose)	GTP-206 (4.5 x 10^{12} GC/Animal – Low Dose)									
	Study Day	BL	0	7±1	14±2	28±3	60±3	90±4	120±4	150±4	180±5
Complete Blood Counts (Hematology) & Plasma Coagulation	WBC (10^3/µL)	7.34	6.21	10.64	8.52	8.16	5.73	6.47	8.03	8.82	6.54
	RBC (10^6/µL)	6.22	5.96	5.74	5.60	5.98	6.18	6.01	6.10	5.94	6.19
	HB (g/dL)	13.5	12.9	12.2	12.0	12.6	13.1	12.4	13.2	12.7	13.2
	HCT (%)	44.3	43.7	41.1	40.4	43.3	43.5	42.2	43.5	42.0	43.3
	MCV (fL)	71.2	73.3	71.5	72.3	72.5	70.4	70.1	71.3	70.8	69.9
	MCH (pg)	21.7	21.7	21.2	21.4	21.0	21.2	20.7	21.6	21.4	21.4
	MCHC (g/dL)	30.5	29.6	29.6	29.7	29.0	30.0	29.5	30.3	30.2	30.6
	PLT (10^3/µL)	365	358	374	397	360	334	359	341	348	334
	NEU% (%)	50.3	37.0	83.6	82.6	69.1	43.9	67.5	59.0	54.5	47.2
	NEU (10^3/µL)	3.69	2.30	8.89	7.03	5.64	2.52	4.37	4.74	4.81	3.08
	LYMPH% (%)	43.2	54.9	13.4	14.2	26.5	49.4	27.4	35.8	39.8	44.4
	LYMPH (10^3/µL)	3.17	3.41	1.43	1.20	2.16	2.83	1.77	2.87	3.51	2.90
	MONO% (%)	2.2	4.0	1.1	1.1	1.8	2.3	2.5	2.2	2.4	3.9
	MONO (10^3/µL)	0.16	0.25	0.11	0.09	0.15	0.13	0.16	0.18	0.21	0.25
	EOS% (%)	2.9	2.7	1.2	1.3	1.4	2.1	1.3	1.9	1.9	2.1
	EOS (10^3/µL)	0.21	0.17	0.12	0.11	0.11	0.12	0.08	0.15	0.17	0.13
	BAS% (%)	0.6	0.4	0.4	0.4	0.4	0.7	0.4	0.4	0.5	0.8
	BAS (10^3/µL)	0.04	0.02	0.04	0.03	0.03	0.04	0.03	0.03	0.04	0.05
	LUC% (%)	0.9	1.1	0.4	0.5	0.7	1.5	1.0	0.8	0.8	1.7
	LUC (10^3/µL)	0.07	0.07	0.04	0.05	0.06	0.09	0.06	0.06	0.07	0.11
	MAC	N/A	N/A	N/A	N/A	N/A	N/A	N/A	N/A	N/A	N/A
	LPLT	N/A	N/A	N/A	N/A	N/A	N/A	N/A	N/A	N/A	N/A
	CPLT	N/A	N/A	N/A	N/A	N/A	N/A	N/A	N/A	N/A	N/A
	ANIS	N/A	N/A	N/A	N/A	N/A	N/A	N/A	N/A	N/A	N/A
	ATYP	N/A	N/A	N/A	N/A	N/A	N/A	N/A	N/A	N/A	N/A
	COMMENT	HEM	HEM	HEM	N/A	N/A	N/A	N/A	N/A	N/A	N/A
	PT (sec)	11.7	11.0	11.3	11.4	10.9	11.2	11.0	11.5	NOSA	11.3
	APTT (sec)	21.7	21.8	22.1	21.3	23.1	22.3	22.8	23.3	NOSA	24.1
	FIB (mg/dL)	157	153	172	163	149	138	197	141	NOSA	218
	DIME (ng/mL)	289.5	262.1	354.9	364.1	301.9	298.8	599.6	355.6	NOSA	404.9
	FDP (FEU)	<2.5	<2.5	<2.5	<2.5	<2.5	<2.5	<2.5	<2.5	NOSA	<2.5

	Group (Necropsy Day)	Group 6 (Necropsy Day 180±5)									
	Animal ID (Sex)	18-042 (Female)									
	Treatment (Dose)	GTP-206 (4.5 x 10^{12} GC/Animal – Low Dose)									
	Study Day	BL	0	7±1	14±2	28±3	60±3	90±4	120±4	150±4	180±5
Serum Chemistry	BUN (mg/dL)	30	21	23	24	21	22	14	15	17	18
	CREA (mg/dL)	0.5	0.6	0.5	0.5	0.5	0.5	0.5	0.5	0.6	0.5
	GLU (mg/dL)	103	103	86	77	88	99	81	81	85	104
	NA (mmol/L)	148	146	148	148	146	146	143	150	145	145
	K (mmol/L)	3.2	3.5	3.1	3.1	3.4	3.2	4.2	3.6	3.5	3.3
	CL (mmol/L)	107	108	110	110	108	109	105	110	106	107
	ALP (U/L)	603	720	648	592	641	734	646	668	654	592
	ALT (U/L)	22	20	20	21	28	21	26	22	25	17
	AST (U/L)	35	32	28	33	34	31	91	31	29	22
	TBIL (mg/dL)	0.2	0.1	0.2	0.2	0.2	0.1	0.2	0.2	0.1	0.1
	LDH (U/L)	241	252	212	245	267	228	800	196	263	190
	CPK (U/L)	354	730	260	837	421	673	8207	241	407	260
	GGT (U/L)	91	89	97	105	99	110	102	107	102	96
	TPRO (g/dL)	5.3	5.8	6.1	6.1	6.0	5.9	6.3	6.5	6.1	6.3
	ALB (g/dL)	3.9	3.9	4.0	3.9	3.9	3.9	4.1	4.0	4.0	4.0
	GLOB (g/dL)	1.4	1.9	2.1	2.2	2.1	2.0	2.2	2.5	2.1	2.3
	A/G	2.79	2.05	1.90	1.77	1.86	1.95	1.86	1.60	1.90	1.74
	CA (mg/dL)	9.7	10.1	9.5	9.5	9.9	9.3	9.5	9.9	9.9	9.8
	PHOS (mg/dL)	5.2	4.6	4.2	4.0	4.7	4.2	5.9	6.7	5.4	4.8
	ALD (U/L)	19.4	22.3	17.3	22.7	18.1	19.7	109.6	16.0	17.9	11.4
	CKMM (U/L)	QNS	604	193	716	320	551	7838	177	294	184
	CKM% (%)	QNS	82.7	74.4	85.5	75.9	81.9	95.5	73.4	72.2	70.7
	CKMB (U/L)	QNS	65	34	62	46	49	140	26	50	44
	CMB% (%)	QNS	8.9	12.9	7.4	10.9	7.3	1.7	10.9	12.4	16.9
	CKBB (U/L)	QNS	61	33	59	56	72	230	38	63	32
	CKB% (%)	QNS	8.4	12.6	7.1	13.2	10.7	2.8	15.6	15.5	12.4
	COMMENT	N/A	N/A	N/A	N/A	N/A	N/A	HEM	N/A	N/A	N/A

	Group (Necropsy Day)	Group 6 (Necropsy Day 180±5)									
	Animal ID (Sex)	18-121 (Female)									
	Treatment (Dose)	GTP-206 (4.5 x 10^{12} GC/Animal – Low Dose)									
	Study Day	BL	0	7±1	14±2	28±3	60±3	90±4	120±4	150±4	180±5
Complete Blood Counts (Hematology) & Plasma Coagulation	WBC (10^3/µL)	8.66	8.08	8.30	11.38	9.27	7.71	8.83	8.33	8.51	8.21
	RBC (10^6/µL)	6.24	5.80	5.75	5.88	5.89	5.80	5.87	5.98	5.84	5.91
	HB (g/dL)	14.0	12.7	12.6	13.0	12.9	12.8	12.8	13.1	12.9	12.7
	HCT (%)	45.0	42.0	41.3	42.5	42.5	41.9	42.3	42.8	41.6	42.1
	MCV (fL)	72.2	72.5	71.9	72.3	72.2	72.2	72.1	71.5	71.2	71.3
	MCH (pg)	22.4	21.9	21.9	22.0	21.9	22.1	21.8	22.0	22.1	21.4
	MCHC (g/dL)	31.0	30.2	30.4	30.5	30.4	30.6	30.2	30.7	31.0	30.1
	PLT (10^3/µL)	323	263	363	376	354	346	332	318	337	235
	NEU% (%)	41.9	37.3	64.0	73.7	69.9	45.1	61.6	60.0	49.6	63.0
	NEU (10^3/µL)	3.62	3.01	5.31	8.39	6.47	3.48	5.44	5.00	4.22	5.17
	LYMPH% (%)	49.7	55.3	30.6	22.9	24.7	50.0	30.0	34.1	43.9	30.3
	LYMPH (10^3/µL)	4.30	4.47	2.54	2.60	2.29	3.85	2.65	2.84	3.73	2.49
	MONO% (%)	2.7	3.5	2.2	1.3	2.7	1.9	5.1	3.3	2.8	2.9
	MONO (10^3/µL)	0.23	0.28	0.18	0.15	0.25	0.15	0.45	0.27	0.24	0.24
	EOS% (%)	3.0	2.2	1.7	0.9	0.9	1.0	1.5	1.3	2.0	1.7
	EOS (10^3/µL)	0.26	0.18	0.14	0.10	0.08	0.08	0.13	0.11	0.17	0.14
	BAS% (%)	1.0	0.5	0.7	0.4	0.8	0.6	1.0	0.4	0.8	1.1
	BAS (10^3/µL)	0.08	0.04	0.05	0.05	0.07	0.04	0.09	0.04	0.07	0.09
	LUC% (%)	1.8	1.3	0.8	0.8	1.1	1.5	0.9	0.8	1.0	1.0
	LUC (10^3/µL)	0.16	0.11	0.07	0.09	0.10	0.11	0.08	0.06	0.08	0.08
	MAC	N/A	N/A	N/A	N/A	N/A	N/A	N/A	N/A	N/A	N/A
	LPLT	N/A	N/A	N/A	N/A	N/A	N/A	N/A	N/A	N/A	N/A
	CPLT	N/A	+	N/A	N/A	+	N/A	+	+	+	+
	ANIS	N/A	N/A	N/A	N/A	N/A	N/A	N/A	N/A	N/A	N/A
	ATYP	N/A	N/A	N/A	N/A	N/A	N/A	N/A	N/A	N/A	N/A
	COMMENT	N/A	N/A	N/A	N/A	N/A	N/A	HEM	N/A	N/A	N/A
	PT (sec)	10.4	10.3	10.3	10.0	9.8	10.1	10.7	10.7	10.3	10.5
	APTT (sec)	19.9	20.7	20.7	19.9	20.1	20.7	20.3	21.3	20.7	21.2
	FIB (mg/dL)	218	208	224	238	216	201	119	208	201	199
	DIME (ng/mL)	538.4	497.3	458.3	687.8	717.6	605.1	923.9	715.7	520.6	495.1
	FDP (FEU)	<2.5	2.5	<2.5	2.5	2.5	<2.5	2.5	2.5	<2.5	<2.5

	Group (Necropsy Day)	Group 6 (Necropsy Day 180±5)									
	Animal ID (Sex)	18-121 (Female)									
	Treatment (Dose)	GTP-206 (4.5 x 10^{12} GC/Animal – Low Dose)									
	**Study Day**	**BL**	**0**	**7±1**	**14±2**	**28±3**	**60±3**	**90±4**	**120±4**	**150±4**	**180±5**
**Serum Chemistry**	**BUN (mg/dL)**	QNS	20	20	20	18	19	14	18	16	17
	**CREA (mg/dL)**	QNS	0.6	0.5	0.5	0.4	0.5	0.4	0.5	0.5	0.5
	**GLU (mg/dL)**	QNS	99	88	91	86	97	92	76	100	75
	**NA (mmol/L)**	148	147	148	147	146	147	147	151	147	148
	**K (mmol/L)**	3.5	3.2	3.4	3.3	3.4	3.2	3.6	3.7	3.3	3.5
	**CL (mmol/L)**	107	107	107	108	105	106	107	109	106	108
	**ALP (U/L)**	669	696	702	624	636	740	797	749	680	703
	**ALT (U/L)**	30	24	32	28	27	34	32	29	29	23
	**AST (U/L)**	QNS	28	35	28	32	26	37	31	33	31
	**TBIL (mg/dL)**	QNS	0.2	0.2	0.2	0.2	0.2	0.2	0.3	0.2	0.2
	**LDH (U/L)**	QNS	246	375	226	253	222	294	208	314	275
	**CPK (U/L)**	QNS	360	119	104	173	142	899	273	793	580
	**GGT (U/L)**	QNS	77	87	93	85	91	91	100	93	92
	**TPRO (g/dL)**	QNS	6.2	6.4	6.5	6.4	6.0	6.5	6.8	6.3	6.6
	**ALB (g/dL)**	4.2	4.0	4.2	4.2	4.0	3.8	4.2	4.3	4.2	4.3
	**GLOB (g/dL)**	QNS	2.2	2.2	2.3	2.4	2.2	2.3	2.5	2.1	2.3
	**A/G**	QNS	1.82	1.91	1.83	1.67	1.73	1.83	1.72	2.00	1.87
	**CA (mg/dL)**	QNS	10.1	9.9	9.7	9.9	9.2	9.8	10.0	10.0	10.1
	**PHOS (mg/dL)**	QNS	5.0	5.7	5.9	6.1	5.9	6.5	7.3	6.6	6.3
	**ALD (U/L)**	QNS	18.9	25.1	17.9	18.1	16.6	28.1	17.9	23.0	20.5
	**CKMM (U/L)**	QNS	278	62	56	108	76	763	214	703	503
	**CKM% (%)**	QNS	77.2	51.8	53.8	62.7	53.6	84.9	78.4	88.7	86.8
	**CKMB (U/L)**	QNS	45	26	30	44	36	56	26	46	39
	**CMB% (%)**	QNS	12.6	21.5	28.7	25.5	25.4	6.2	9.4	5.8	6.7
	**CKBB (U/L)**	QNS	37	32	18	20	30	80	33	44	38
	**CKB% (%)**	QNS	10.2	26.7	17.6	11.8	21.0	8.9	12.2	5.5	6.5
	**COMMENT**	N/A	N/A	N/A	N/A	N/A	N/A	N/A	N/A	N/A	N/A

	Group (Necropsy Day)	Group 6 (Necropsy Day 180±5)									
	Animal ID (Sex)	18-171 (Male)									
	Treatment (Dose)	GTP-206 (4.5 x 10^{12} GC/Animal – Low Dose)									
	Study Day	BL	0	7±1	14±2	28±3	60±3	90±4	120±4	150±4	180±5
Complete Blood Counts (Hematology) & Plasma Coagulation	WBC (10^3/µL)	13.66	10.55	8.86	9.70	20.47	22.57	12.60	11.33	15.81	12.79
	RBC (10^6/µL)	5.69	5.58	5.57	5.64	5.68	6.17	5.69	5.77	5.57	5.65
	HB (g/dL)	12.7	12.4	12.3	12.3	12.4	13.2	12.1	12.5	12.1	12.2
	HCT (%)	42.3	41.9	41.8	41.4	40.8	43.4	39.5	40.4	39.6	39.9
	MCV (fL)	74.3	75.2	75.1	73.5	71.8	70.3	69.4	70.1	71.1	70.6
	MCH (pg)	22.3	22.2	22.2	21.7	21.8	21.4	21.3	21.7	21.8	21.5
	MCHC (g/dL)	30.0	29.6	29.5	29.6	30.3	30.4	30.6	31.0	30.7	30.5
	PLT (10^3/µL)	402	386	359	414	385	386	298	365	375	400
	NEU% (%)	74.7	50.9	65.1	53.7	83.4	82.4	62.3	44.3	76.0	75.0
	NEU (10^3/µL)	10.21	5.37	5.77	5.22	17.07	18.60	7.85	5.02	12.01	9.60
	LYMPH% (%)	20.9	42.0	30.2	42.1	14.2	14.6	33.0	48.3	20.1	21.5
	LYMPH (10^3/µL)	2.86	4.43	2.68	4.08	2.90	3.29	4.16	5.48	3.17	2.75
	MONO% (%)	2.6	2.8	2.5	2.0	1.1	1.6	2.4	2.9	1.9	1.6
	MONO (10^3/µL)	0.36	0.30	0.22	0.20	0.22	0.37	0.30	0.33	0.30	0.20
	EOS% (%)	0.9	1.7	0.8	0.8	0.5	0.2	1.0	2.3	1.0	0.5
	EOS (10^3/µL)	0.13	0.18	0.07	0.07	0.10	0.05	0.12	0.26	0.15	0.06
	BAS% (%)	0.4	1.1	0.5	0.4	0.4	0.8	0.7	0.8	0.5	0.8
	BAS (10^3/µL)	0.05	0.12	0.04	0.04	0.09	0.18	0.09	0.09	0.07	0.10
	LUC% (%)	0.5	1.5	0.9	0.9	0.4	0.4	0.6	1.3	0.6	0.6
	LUC (10^3/µL)	0.06	0.16	0.08	0.09	0.08	0.09	0.07	0.15	0.10	0.08
	MAC	N/A	N/A	+	N/A	N/A	N/A	N/A	N/A	N/A	N/A
	LPLT	N/A	N/A	N/A	N/A	N/A	N/A	N/A	N/A	N/A	N/A
	CPLT	N/A	N/A	N/A	N/A	N/A	N/A	N/A	N/A	N/A	N/A
	ANIS	N/A	N/A	N/A	N/A	N/A	N/A	N/A	N/A	N/A	N/A
	ATYP	N/A	N/A	N/A	N/A	N/A	N/A	N/A	N/A	N/A	N/A
	COMMENT	N/A	N/A	N/A	N/A	N/A	HEM	N/A	N/A	N/A	N/A
	PT (sec)	10.7	10.8	10.2	10.7	10.6	11.1	10.9	11.1	11.2	10.7
	APTT (sec)	19.8	19.9	19.6	20.9	19.1	20.5	20.3	21.1	20.6	21.2
	FIB (mg/dL)	164	174	204	191	187	146	163	174	166	169
	DIME (ng/mL)	528.1	430.3	326.3	665.4	421.7	334.7	408.8	302.8	467.6	319.9
	FDP (FEU)	<2.5	<2.5	<2.5	2.5	<2.5	<2.5	<2.5	<2.5	<2.5	<2.5

	Group (Necropsy Day)	Group 6 (Necropsy Day 180±5)									
	Animal ID (Sex)	18-171 (Male)									
	Treatment (Dose)	GTP-206 (4.5 x 10^{12} GC/Animal – Low Dose)									
	Study Day	BL	0	7±1	14±2	28±3	60±3	90±4	120±4	150±4	180±5
Serum Chemistry	BUN (mg/dL)	22	23	22	15	23	25	17	17	22	21
	CREA (mg/dL)	0.6	0.6	0.6	0.7	0.6	0.6	0.4	0.5	0.5	0.6
	GLU (mg/dL)	128	116	73	91	80	69	107	86	61	73
	NA (mmol/L)	149	150	148	149	147	146	149	149	145	147
	K (mmol/L)	3.6	3.2	3.6	3.4	3.2	3.3	3.2	3.2	3.3	3.6
	CL (mmol/L)	107	108	106	107	107	107	108	107	106	109
	ALP (U/L)	517	571	424	468	399	485	428	425	445	494
	ALT (U/L)	39	34	32	28	34	33	29	37	36	43
	AST (U/L)	23	21	31	24	29	24	23	26	31	72
	TBIL (mg/dL)	0.1	0.1	0.2	0.1	0.2	0.2	0.2	0.2	0.2	0.2
	LDH (U/L)	350	234	430	306	281	261	221	306	376	603
	CPK (U/L)	555	98	790	141	460	218	229	189	454	4734
	GGT (U/L)	85	86	84	80	89	87	73	82	98	98
	TPRO (g/dL)	6.3	6.2	6.7	6.2	6.4	6.3	6.0	6.0	6.3	5.9
	ALB (g/dL)	4.0	4.0	4.2	3.9	4.1	4.2	3.8	3.9	3.9	3.9
	GLOB (g/dL)	2.3	2.2	2.5	2.3	2.3	2.1	2.2	2.1	2.4	2.0
	A/G	1.74	1.82	1.68	1.70	1.78	2.00	1.73	1.86	1.63	1.95
	CA (mg/dL)	9.9	10.2	10.1	9.7	9.3	9.2	8.9	9.6	9.6	9.6
	PHOS (mg/dL)	5.9	4.0	7.3	6.8	6.0	5.9	5.6	4.7	6.0	6.3
	ALD (U/L)	28.1	17.4	30.3	21.9	23.2	14.7	15.8	23.2	23.5	76.2
	CKMM (U/L)	459	39	690	75	371	155	146	123	383	4502
	CKM% (%)	82.7	40.3	87.4	53.1	80.6	71.1	63.6	64.9	84.4	95.1
	CKMB (U/L)	60	30	66	26	44	28	37	33	45	99
	CMB% (%)	10.8	30.5	8.3	18.3	9.6	12.9	16.1	17.7	10.0	2.1
	CKBB (U/L)	36	29	34	40	46	35	46	33	25	133
	CKB% (%)	6.5	29.2	4.3	28.6	9.9	16.1	20.3	17.5	5.6	2.8
	COMMENT	N/A	N/A	N/A	N/A	N/A	N/A	N/A	N/A	N/A	N/A

	Group (Necropsy Day)	Group 3 (Necropsy Day 90±4)									
	Animal ID (Sex)	18-167 (Male)									
	Treatment (Dose)	GTP-206 (1.5 x 10^{13} GC/Animal – Mid-Dose)									
	**Study Day**	**BL**	**0**	**7±1**	**14±2**	**28±3**	**60±3**	**90±4**	**120±4**	**150±4**	**180±5**
**Complete Blood Counts (Hematology) & Plasma Coagulation**	**WBC (10^3/µL)**	9.58	7.16	7.78	8.04	9.56	9.83	7.28	N/A	N/A	N/A
	**RBC (10^6/µL)**	5.87	5.67	5.89	6.05	6.09	6.20	5.85	N/A	N/A	N/A
	**HB (g/dL)**	13.0	12.8	13.0	13.1	13.2	13.3	12.7	N/A	N/A	N/A
	**HCT (%)**	44.0	42.3	44.0	44.9	44.7	45.4	42.6	N/A	N/A	N/A
	**MCV (fL)**	74.9	74.7	74.8	74.2	73.4	73.3	72.9	N/A	N/A	N/A
	**MCH (pg)**	22.2	22.5	22.2	21.6	21.7	21.5	21.8	N/A	N/A	N/A
	**MCHC (g/dL)**	29.6	30.1	29.6	29.1	29.5	29.3	29.9	N/A	N/A	N/A
	**PLT (10^3/µL)**	486	566	568	516	560	532	525	N/A	N/A	N/A
	**NEU% (%)**	67.4	36.1	68.7	39.8	57.1	54.4	41.5	N/A	N/A	N/A
	**NEU (10^3/µL)**	6.45	2.58	5.34	3.20	5.46	5.34	3.02	N/A	N/A	N/A
	**LYMPH% (%)**	27.5	52.6	28.0	51.0	36.4	37.6	48.8	N/A	N/A	N/A
	**LYMPH (10^3/µL)**	2.64	3.77	2.18	4.10	3.48	3.70	3.55	N/A	N/A	N/A
	**MONO% (%)**	2.6	3.9	1.6	4.4	2.4	3.8	4.4	N/A	N/A	N/A
	**MONO (10^3/µL)**	0.25	0.28	0.12	0.35	0.23	0.38	0.32	N/A	N/A	N/A
	**EOS% (%)**	1.5	5.7	0.5	2.9	2.6	2.9	4.1	N/A	N/A	N/A
	**EOS (10^3/µL)**	0.15	0.41	0.04	0.23	0.25	0.29	0.30	N/A	N/A	N/A
	**BAS% (%)**	0.4	0.7	0.8	0.9	0.6	0.4	0.4	N/A	N/A	N/A
	**BAS (10^3/µL)**	0.04	0.05	0.06	0.08	0.06	0.04	0.03	N/A	N/A	N/A
	**LUC% (%)**	0.5	0.9	0.4	0.9	0.9	0.8	0.9	N/A	N/A	N/A
	**LUC (10^3/µL)**	0.05	0.07	0.03	0.07	0.09	0.08	0.06	N/A	N/A	N/A
	**MAC**	N/A	N/A	N/A	N/A	N/A	N/A	N/A	N/A	N/A	N/A
	**LPLT**	N/A	N/A	N/A	N/A	N/A	N/A	N/A	N/A	N/A	N/A
	**CPLT**	N/A	N/A	N/A	+	N/A	N/A	N/A	N/A	N/A	N/A
	**ANIS**	N/A	N/A	N/A	N/A	N/A	N/A	N/A	N/A	N/A	N/A
	**ATYP**	N/A	N/A	N/A	N/A	N/A	N/A	N/A	N/A	N/A	N/A
	**COMMENT**	N/A	N/A	N/A	N/A	N/A	N/A	N/A	N/A	N/A	N/A
	**PT (sec)**	10.6	10.7	10.7	10.6	10.2	10.8	11.3	N/A	N/A	N/A
	**APTT (sec)**	19.5	20.0	19.8	20.5	19.3	20.7	21.5	N/A	N/A	N/A
	**FIB (mg/dL)**	158	167	193	154	189	161	157	N/A	N/A	N/A
	**DIME (ng/mL)**	341.3	270.9	202.6	156.4	219.3	261.0	216.2	N/A	N/A	N/A
	**FDP (FEU)**	<2.5	<2.5	<2.5	<2.5	<2.5	<2.5	<2.5	N/A	N/A	N/A

	Group (Necropsy Day)	Group 3 (Necropsy Day 90±4)									
	Animal ID (Sex)	18-167 (Male)									
	Treatment (Dose)	GTP-206 (1.5 x 10^{13} GC/Animal – Mid-Dose)									
	**Study Day**	**BL**	**0**	**7±1**	**14±2**	**28±3**	**60±3**	**90±4**	**120±4**	**150±4**	**180±5**
**Serum Chemistry**	**BUN (mg/dL)**	19	19	21	23	19	24	23	N/A	N/A	N/A
	**CREA (mg/dL)**	0.5	0.5	0.5	0.5	0.4	0.4	0.4	N/A	N/A	N/A
	**GLU (mg/dL)**	82	84	83	140	61	75	133	N/A	N/A	N/A
	**NA (mmol/L)**	148	145	148	147	146	146	147	N/A	N/A	N/A
	**K (mmol/L)**	3.3	3.7	3.8	3.2	3.5	3.5	3.9	N/A	N/A	N/A
	**CL (mmol/L)**	108	107	109	107	107	107	107	N/A	N/A	N/A
	**ALP (U/L)**	1184	953	1116	975	1130	1228	1156	N/A	N/A	N/A
	**ALT (U/L)**	26	21	18	21	23	18	18	N/A	N/A	N/A
	**AST (U/L)**	39	30	28	27	31	31	35	N/A	N/A	N/A
	**TBIL (mg/dL)**	0.2	0.2	0.2	0.2	0.2	0.1	0.1	N/A	N/A	N/A
	**LDH (U/L)**	394	357	350	265	337	338	619	N/A	N/A	N/A
	**CPK (U/L)**	789	243	113	220	167	215	208	N/A	N/A	N/A
	**GGT (U/L)**	104	95	94	87	98	96	99	N/A	N/A	N/A
	**TPRO (g/dL)**	5.9	5.5	6.0	5.7	6.0	5.8	5.1	N/A	N/A	N/A
	**ALB (g/dL)**	3.9	4.0	4.0	3.9	3.9	3.8	3.4	N/A	N/A	N/A
	**GLOB (g/dL)**	2.0	1.5	2.0	1.8	2.1	2.0	1.7	N/A	N/A	N/A
	**A/G**	1.95	2.67	2.00	2.17	1.86	1.90	2.00	N/A	N/A	N/A
	**CA (mg/dL)**	9.8	10.1	10.1	9.8	9.9	9.5	9.5	N/A	N/A	N/A
	**PHOS (mg/dL)**	6.2	7.4	7.5	6.9	6.0	6.1	7.3	N/A	N/A	N/A
	**ALD (U/L)**	42.6	35.1	23.8	23.7	31.6	25.0	34.5	N/A	N/A	N/A
	**CKMM (U/L)**	679	158	56	135	100	134	128	N/A	N/A	N/A
	**CKM% (%)**	86.0	64.9	49.4	61.2	59.8	62.3	61.3	N/A	N/A	N/A
	**CKMB (U/L)**	60	41	29	41	30	43	36	N/A	N/A	N/A
	**CMB% (%)**	7.6	17.0	25.4	18.7	18.0	20.1	17.5	N/A	N/A	N/A
	**CKBB (U/L)**	50	44	28	44	37	38	44	N/A	N/A	N/A
	**CKB% (%)**	6.4	18.1	25.2	20.1	22.2	17.6	21.3	N/A	N/A	N/A
	**COMMENT**	N/A	N/A	N/A	N/A	N/A	N/A	N/A	N/A	N/A	N/A

	Group (Necropsy Day)	Group 3 (Necropsy Day 90±4)									
	Animal ID (Sex)	18-176 (Male)									
	Treatment (Dose)	GTP-206 (1.5 x 10^{13} GC/Animal – Mid-Dose)									
	Study Day	BL	0	7±1	14±2	28±3	60±3	90±4	120±4	150±4	180±5
Complete Blood Counts (Hematology) & Plasma Coagulation	WBC (10^3/µL)	11.16	12.13	12.05	10.67	18.41	10.86	8.80	N/A	N/A	N/A
	RBC (10^6/µL)	5.47	5.64	5.53	5.37	5.42	5.46	5.41	N/A	N/A	N/A
	HB (g/dL)	13.4	13.6	13.3	13.1	12.9	13.6	13.0	N/A	N/A	N/A
	HCT (%)	45.6	46.3	45.9	43.7	45.4	44.1	43.3	N/A	N/A	N/A
	MCV (fL)	83.3	82.0	83.1	81.4	83.7	80.8	80.1	N/A	N/A	N/A
	MCH (pg)	24.5	24.1	24.1	24.3	23.9	24.9	24.0	N/A	N/A	N/A
	MCHC (g/dL)	29.5	29.4	29.0	29.9	28.5	30.9	29.9	N/A	N/A	N/A
	PLT (10^3/µL)	312	444	439	484	479	288	383	N/A	N/A	N/A
	NEU% (%)	66.9	63.8	53.9	46.5	73.6	39.9	62.0	N/A	N/A	N/A
	NEU (10^3/µL)	7.47	7.74	6.50	4.96	13.54	4.33	5.46	N/A	N/A	N/A
	LYMPH% (%)	29.5	32.4	40.3	47.1	22.7	51.2	34.6	N/A	N/A	N/A
	LYMPH (10^3/µL)	3.29	3.94	4.85	5.03	4.17	5.56	3.04	N/A	N/A	N/A
	MONO% (%)	1.6	1.4	2.7	2.9	1.5	5.9	2.0	N/A	N/A	N/A
	MONO (10^3/µL)	0.18	0.17	0.33	0.30	0.28	0.64	0.18	N/A	N/A	N/A
	EOS% (%)	0.7	1.1	1.1	1.5	0.7	1.0	0.5	N/A	N/A	N/A
	EOS (10^3/µL)	0.08	0.13	0.13	0.16	0.14	0.11	0.04	N/A	N/A	N/A
	BAS% (%)	0.5	0.8	0.7	0.8	0.8	0.5	0.3	N/A	N/A	N/A
	BAS (10^3/µL)	0.05	0.09	0.09	0.08	0.14	0.06	0.03	N/A	N/A	N/A
	LUC% (%)	0.8	0.6	1.2	1.4	0.7	1.4	0.5	N/A	N/A	N/A
	LUC (10^3/µL)	0.09	0.07	0.15	0.15	0.13	0.16	0.05	N/A	N/A	N/A
	MAC	+++	+++	+++	+++	+++	++	++	N/A	N/A	N/A
	LPLT	N/A	N/A	N/A	N/A	N/A	N/A	N/A	N/A	N/A	N/A
	CPLT	N/A	N/A	N/A	N/A	N/A	+	N/A	N/A	N/A	N/A
	ANIS	N/A	N/A	N/A	N/A	N/A	N/A	N/A	N/A	N/A	N/A
	ATYP	N/A	N/A	N/A	N/A	N/A	N/A	N/A	N/A	N/A	N/A
	COMMENT	N/A	N/A	N/A	N/A	N/A	HEM	N/A	N/A	N/A	N/A
	PT (sec)	10.0	10.1	10.0	10.1	10.0	10.6	10.7	N/A	N/A	N/A
	APTT (sec)	20.8	19.9	21.1	22.2	21.4	23.7	22.9	N/A	N/A	N/A
	FIB (mg/dL)	216	199	218	232	216	181	181	N/A	N/A	N/A
	DIME (ng/mL)	790.8	9000.	259.6	588.9	407.2	783.9	448.5	N/A	N/A	N/A
	FDP (FEU)	<2.5	40.0	<2.5	2.5	<2.5	2.5	<2.5	N/A	N/A	N/A

	Group (Necropsy Day)	Group 3 (Necropsy Day 90±4)									
	**Animal ID (Sex)**	**18-176 (Male)**									
	**Treatment (Dose)**	**GTP-206 (1.5 x 10^{13} GC/Animal – Mid-Dose)**									
	**Study Day**	**BL**	**0**	**7±1**	**14±2**	**28±3**	**60±3**	**90±4**	**120±4**	**150±4**	**180±5**
**Serum Chemistry**	**BUN (mg/dL)**	19	20	16	17	21	24	21	N/A	N/A	N/A
	**CREA (mg/dL)**	0.5	0.6	0.5	0.4	0.4	0.4	0.3	N/A	N/A	N/A
	**GLU (mg/dL)**	63	71	72	72	55	68	125	N/A	N/A	N/A
	**NA (mmol/L)**	150	151	147	146	144	143	147	N/A	N/A	N/A
	**K (mmol/L)**	3.5	3.2	3.2	3.4	3.3	3.5	3.7	N/A	N/A	N/A
	**CL (mmol/L)**	108	108	106	105	104	104	108	N/A	N/A	N/A
	**ALP (U/L)**	593	566	657	566	646	617	667	N/A	N/A	N/A
	**ALT (U/L)**	45	28	37	42	49	39	39	N/A	N/A	N/A
	**AST (U/L)**	38	24	33	37	40	37	34	N/A	N/A	N/A
	**TBIL (mg/dL)**	0.1	0.1	0.1	0.1	0.1	0.1	0.1	N/A	N/A	N/A
	**LDH (U/L)**	451	262	351	348	380	339	320	N/A	N/A	N/A
	**CPK (U/L)**	238	102	395	646	253	303	409	N/A	N/A	N/A
	**GGT (U/L)**	84	84	82	79	93	90	93	N/A	N/A	N/A
	**TPRO (g/dL)**	6.6	6.7	6.4	6.1	6.2	6.1	6.0	N/A	N/A	N/A
	**ALB (g/dL)**	4.1	4.2	4.2	4.0	3.8	3.8	3.8	N/A	N/A	N/A
	**GLOB (g/dL)**	2.5	2.5	2.2	2.1	2.4	2.3	2.2	N/A	N/A	N/A
	**A/G**	1.64	1.68	1.91	1.90	1.58	1.65	1.73	N/A	N/A	N/A
	**CA (mg/dL)**	9.7	9.9	9.7	8.9	9.2	9.0	9.0	N/A	N/A	N/A
	**PHOS (mg/dL)**	6.1	5.7	5.4	5.7	5.6	5.7	6.5	N/A	N/A	N/A
	**ALD (U/L)**	30.4	12.7	27.9	32.6	27.2	24.1	19.4	N/A	N/A	N/A
	**CKMM (U/L)**	180	36	320	563	187	214	290	N/A	N/A	N/A
	**CKM% (%)**	75.5	35.0	81.0	87.2	73.9	70.7	71.0	N/A	N/A	N/A
	**CKMB (U/L)**	39	26	36	44	41	46	66	N/A	N/A	N/A
	**CMB% (%)**	16.4	25.0	9.2	6.8	16.3	15.2	16.1	N/A	N/A	N/A
	**CKBB (U/L)**	19	41	39	39	25	42	53	N/A	N/A	N/A
	**CKB% (%)**	8.1	40.0	9.8	6.0	9.8	14.0	12.9	N/A	N/A	N/A
	**COMMENT**	N/A	N/A	N/A	N/A	N/A	N/A	N/A	N/A	N/A	N/A

	Group (Necropsy Day)	Group 3 (Necropsy Day 90±4)									
	Animal ID (Sex)	18-187 (Female)									
	Treatment (Dose)	GTP-206 (1.5 x 10^{13} GC/Animal – Mid-Dose)									
	Study Day	BL	0	7±1	14±2	28±3	60±3	90±4	120±4	150±4	180±5
Complete Blood Counts (Hematology) & Plasma Coagulation	WBC (10^3/µL)	12.83	11.21	8.83	8.84	11.61	10.02	7.39	N/A	N/A	N/A
	RBC (10^6/µL)	5.65	5.70	5.45	5.64	5.65	5.79	5.32	N/A	N/A	N/A
	HB (g/dL)	13.2	13.3	12.5	12.9	12.9	13.4	12.1	N/A	N/A	N/A
	HCT (%)	42.0	43.0	40.5	41.4	42.0	42.9	39.4	N/A	N/A	N/A
	MCV (fL)	74.3	75.4	74.4	73.5	74.5	74.2	74.0	N/A	N/A	N/A
	MCH (pg)	23.3	23.3	23.0	22.9	22.9	23.1	22.7	N/A	N/A	N/A
	MCHC (g/dL)	31.4	31.0	30.9	31.1	30.7	31.1	30.7	N/A	N/A	N/A
	PLT (10^3/µL)	402	420	389	385	352	336	397	N/A	N/A	N/A
	NEU% (%)	65.0	62.9	52.1	46.4	47.9	50.1	51.4	N/A	N/A	N/A
	NEU (10^3/µL)	8.34	7.04	4.61	4.10	5.56	5.01	3.80	N/A	N/A	N/A
	LYMPH% (%)	25.7	29.7	38.2	44.4	40.9	40.8	40.1	N/A	N/A	N/A
	LYMPH (10^3/µL)	3.30	3.33	3.38	3.93	4.75	4.09	2.96	N/A	N/A	N/A
	MONO% (%)	2.1	1.5	2.4	2.0	2.0	3.4	2.1	N/A	N/A	N/A
	MONO (10^3/µL)	0.27	0.17	0.21	0.17	0.23	0.34	0.16	N/A	N/A	N/A
	EOS% (%)	6.1	4.2	5.0	4.9	6.9	4.1	5.2	N/A	N/A	N/A
	EOS (10^3/µL)	0.79	0.47	0.45	0.43	0.80	0.41	0.39	N/A	N/A	N/A
	BAS% (%)	0.4	0.7	0.6	0.8	0.8	0.5	0.2	N/A	N/A	N/A
	BAS (10^3/µL)	0.05	0.08	0.05	0.07	0.09	0.05	0.02	N/A	N/A	N/A
	LUC% (%)	0.7	1.0	1.6	1.5	1.5	1.2	0.9	N/A	N/A	N/A
	LUC (10^3/µL)	0.09	0.11	0.14	0.14	0.18	0.12	0.07	N/A	N/A	N/A
	MAC	N/A	N/A	N/A	N/A	+	N/A	N/A	N/A	N/A	N/A
	LPLT	N/A	N/A	N/A	N/A	N/A	N/A	N/A	N/A	N/A	N/A
	CPLT	N/A	N/A	N/A	N/A	N/A	N/A	N/A	N/A	N/A	N/A
	ANIS	N/A	N/A	N/A	N/A	N/A	N/A	N/A	N/A	N/A	N/A
	ATYP	N/A	N/A	N/A	N/A	N/A	N/A	N/A	N/A	N/A	N/A
	COMMENT	N/A	N/A	N/A	N/A	HEM	N/A	N/A	N/A	N/A	N/A
	PT (sec)	11.2	11.4	11.1	11.3	11.4	12.1	11.8	N/A	N/A	N/A
	APTT (sec)	20.5	21.1	21.0	21.6	22.0	23.5	24.0	N/A	N/A	N/A
	FIB (mg/dL)	179	181	163	183	164	155	154	N/A	N/A	N/A
	DIME (ng/mL)	610.2	304.4	581.8	617.0	621.4	355.6	373.7	N/A	N/A	N/A
	FDP (FEU)	<2.5	<2.5	2.5	2.5	2.5	<2.5	<2.5	N/A	N/A	N/A

	Group (Necropsy Day)	Group 3 (Necropsy Day 90±4)									
	Animal ID (Sex)	18-187 (Female)									
	Treatment (Dose)	GTP-206 (1.5 x 10^{13} GC/Animal – Mid-Dose)									
	Study Day	BL	0	7±1	14±2	28±3	60±3	90±4	120±4	150±4	180±5
Serum Chemistry	BUN (mg/dL)	21	21	15	14	19	18	22	N/A	N/A	N/A
	CREA (mg/dL)	0.4	0.5	0.5	0.4	0.4	0.4	0.3	N/A	N/A	N/A
	GLU (mg/dL)	129	104	129	131	96	87	136	N/A	N/A	N/A
	NA (mmol/L)	147	148	145	145	144	145	146	N/A	N/A	N/A
	K (mmol/L)	3.2	3.3	3.2	3.3	3.3	3.6	3.2	N/A	N/A	N/A
	CL (mmol/L)	107	107	106	106	105	107	108	N/A	N/A	N/A
	ALP (U/L)	968	924	973	924	1052	1056	1005	N/A	N/A	N/A
	ALT (U/L)	18	15	22	31	28	24	20	N/A	N/A	N/A
	AST (U/L)	34	24	29	39	36	34	26	N/A	N/A	N/A
	TBIL (mg/dL)	0.2	0.2	0.2	0.1	0.2	0.2	0.1	N/A	N/A	N/A
	LDH (U/L)	341	244	285	350	400	327	234	N/A	N/A	N/A
	CPK (U/L)	824	271	211	281	214	815	233	N/A	N/A	N/A
	GGT (U/L)	75	76	73	72	78	84	78	N/A	N/A	N/A
	TPRO (g/dL)	6.1	6.1	5.8	5.9	5.8	5.8	5.6	N/A	N/A	N/A
	ALB (g/dL)	3.9	4.0	3.8	3.8	3.6	3.7	3.5	N/A	N/A	N/A
	GLOB (g/dL)	2.2	2.1	2.0	2.1	2.2	2.1	2.1	N/A	N/A	N/A
	A/G	1.77	1.90	1.90	1.81	1.64	1.76	1.67	N/A	N/A	N/A
	CA (mg/dL)	9.6	10.0	9.6	9.4	9.3	9.3	9.2	N/A	N/A	N/A
	PHOS (mg/dL)	5.3	7.1	6.4	5.6	6.4	7.1	7.0	N/A	N/A	N/A
	ALD (U/L)	25.5	14.5	22.4	22.3	24.2	24.3	15.9	N/A	N/A	N/A
	CKMM (U/L)	712	139	133	126	97	660	146	N/A	N/A	N/A
	CKM% (%)	86.4	51.4	63.2	45.0	45.5	81.0	62.8	N/A	N/A	N/A
	CKMB (U/L)	58	44	29	66	44	56	41	N/A	N/A	N/A
	CMB% (%)	7.0	16.3	13.7	23.5	20.6	6.9	17.4	N/A	N/A	N/A
	CKBB (U/L)	54	88	49	89	73	99	46	N/A	N/A	N/A
	CKB% (%)	6.6	32.4	23.1	31.6	33.9	12.1	19.9	N/A	N/A	N/A
	COMMENT	N/A	N/A	N/A	N/A	N/A	N/A	N/A	N/A	N/A	N/A

	Group (Necropsy Day)	Group 7 (Necropsy Day 180±5)									
	Animal ID (Sex)	18-055 (Male)									
	Treatment (Dose)	GTP-206 (1.5 x 10^{13} GC/Animal – Mid-Dose)									
	Study Day	BL	0	7±1	14±2	28±3	60±3	90±4	120±4	150±4	180±5
Complete Blood Counts (Hematology) & Plasma Coagulation	WBC (10^3/µL)	7.91	7.07	7.11	6.97	9.27	8.54	7.00	9.17	7.25	5.78
	RBC (10^6/µL)	5.03	4.95	5.24	5.42	5.41	5.57	5.62	5.41	5.08	5.11
	HB (g/dL)	11.4	11.5	11.6	12.1	12.2	12.5	12.5	12.6	11.7	11.8
	HCT (%)	37.7	37.1	39.8	40.7	40.5	41.3	41.4	39.9	38.8	38.5
	MCV (fL)	74.9	75.1	76.1	75.1	74.8	74.1	73.6	73.7	76.4	75.3
	MCH (pg)	22.7	23.2	22.2	22.3	22.5	22.4	22.2	23.3	23.1	23.1
	MCHC (g/dL)	30.3	30.9	29.2	29.7	30.1	30.2	30.1	31.6	30.3	30.7
	PLT (10^3/µL)	527	572	509	544	430	465	468	442	464	433
	NEU% (%)	45.8	46.5	32.9	28.4	30.4	20.3	15.1	21.5	26.0	24.0
	NEU (10^3/µL)	3.63	3.29	2.34	1.98	2.82	1.73	1.05	1.97	1.89	1.39
	LYMPH% (%)	47.4	48.7	60.5	66.3	63.7	73.6	79.8	70.8	67.2	69.5
	LYMPH (10^3/µL)	3.75	3.44	4.30	4.62	5.90	6.28	5.58	6.49	4.88	4.02
	MONO% (%)	2.4	1.4	2.7	1.7	1.9	2.7	1.6	2.4	2.7	2.0
	MONO (10^3/µL)	0.19	0.10	0.19	0.12	0.18	0.23	0.11	0.22	0.19	0.11
	EOS% (%)	2.1	1.5	1.5	1.7	1.8	1.4	0.9	3.1	1.0	1.8
	EOS (10^3/µL)	0.17	0.11	0.11	0.12	0.17	0.12	0.07	0.28	0.07	0.10
	BAS% (%)	0.5	0.4	0.5	0.5	0.6	0.3	0.9	0.8	0.8	0.7
	BAS (10^3/µL)	0.04	0.03	0.03	0.04	0.05	0.03	0.06	0.08	0.05	0.04
	LUC% (%)	1.8	1.5	1.9	1.4	1.6	1.8	1.8	1.4	2.3	2.0
	LUC (10^3/µL)	0.14	0.11	0.14	0.10	0.15	0.15	0.12	0.13	0.17	0.11
	MAC	N/A	+	+	+	+	N/A	N/A	N/A	+	+
	LPLT	N/A	N/A	N/A	N/A	N/A	N/A	N/A	N/A	N/A	N/A
	CPLT	N/A	N/A	N/A	N/A	N/A	N/A	N/A	N/A	+	N/A
	ANIS	N/A	N/A	N/A	N/A	N/A	N/A	N/A	N/A	N/A	N/A
	ATYP	N/A	N/A	N/A	N/A	N/A	N/A	N/A	N/A	N/A	N/A
	COMMENT	N/A	N/A	N/A	N/A	N/A	N/A	N/A	N/A	N/A	N/A
	PT (sec)	10.9	10.7	10.6	10.8	10.8	11.1	11.1	11.1	10.8	10.8
	APTT (sec)	21.7	21.1	21.9	22.3	22.2	23.4	25.2	24.3	23.8	23.9
	FIB (mg/dL)	172	187	187	197	171	161	151	163	195	204
	DIME (ng/mL)	352.8	291.8	289.8	396.4	357.3	340.1	371.1	273.3	315.2	173.5
	FDP (FEU)	<2.5	<2.5	<2.5	<2.5	<2.5	<2.5	<2.5	<2.5	<2.5	<2.5

	Group (Necropsy Day)	Group 7 (Necropsy Day 180±5)									
	Animal ID (Sex)	18-055 (Male)									
	Treatment (Dose)	GTP-206 (1.5 x 10^{13} GC/Animal – Mid-Dose)									
	Study Day	BL	0	7±1	14±2	28±3	60±3	90±4	120±4	150±4	180±5
Serum Chemistry	BUN (mg/dL)	18	22	17	12	26	24	18	19	24	23
	CREA (mg/dL)	0.5	0.5	0.5	0.4	0.4	0.4	0.4	0.4	0.5	0.5
	GLU (mg/dL)	68	82	93	114	71	72	93	99	77	102
	NA (mmol/L)	148	149	147	146	145	145	147	146	145	148
	K (mmol/L)	3.3	3.1	3.1	3.3	3.6	3.3	3.4	3.4	3.6	3.5
	CL (mmol/L)	107	108	106	107	105	106	107	106	106	107
	ALP (U/L)	794	770	863	868	886	838	831	791	768	676
	ALT (U/L)	27	23	40	66	48	35	32	32	35	29
	AST (U/L)	42	23	38	38	48	29	26	27	25	25
	TBIL (mg/dL)	0.2	0.2	0.2	0.1	0.2	0.2	0.2	0.2	0.2	0.1
	LDH (U/L)	347	170	314	192	435	201	192	178	165	202
	CPK (U/L)	1819	237	1183	122	1250	161	209	360	149	370
	GGT (U/L)	87	89	91	91	104	100	102	98	94	92
	TPRO (g/dL)	5.9	6.0	6.0	5.8	5.9	5.9	5.8	6.1	6.0	6.1
	ALB (g/dL)	3.7	3.9	3.8	3.7	3.7	3.6	3.9	3.7	3.8	3.7
	GLOB (g/dL)	2.2	2.1	2.2	2.1	2.2	2.3	1.9	2.4	2.2	2.4
	A/G	1.68	1.86	1.73	1.76	1.68	1.57	2.05	1.54	1.73	1.54
	CA (mg/dL)	9.2	9.5	9.2	9.2	9.1	9.1	9.3	9.4	9.7	9.4
	PHOS (mg/dL)	6.9	6.5	6.6	6.7	7.4	6.9	6.4	6.3	7.7	7.6
	ALD (U/L)	40.6	14.2	33.9	18.4	34.6	16.1	12.8	17.6	15.7	17.1
	CKMM (U/L)	1695	141	1034	66	1084	87	119	271	83	290
	CKM% (%)	93.2	59.6	87.4	54.5	86.7	53.8	56.7	75.2	55.7	78.5
	CKMB (U/L)	62	41	51	25	69	37	36	24	34	36
	CMB% (%)	3.4	17.2	4.3	20.3	5.5	22.7	17.0	6.7	22.9	9.6
	CKBB (U/L)	62	55	98	31	98	38	55	65	32	44
	CKB% (%)	3.4	23.2	8.3	25.2	7.8	23.5	26.2	18.0	21.5	11.9
	COMMENT	N/A	N/A	N/A	N/A	N/A	N/A	N/A	N/A	N/A	N/A

	Group (Necropsy Day)	Group 7 (Necropsy Day 180±5)									
	Animal ID (Sex)	18-181 (Female)									
	Treatment (Dose)	GTP-206 (1.5 x 10^{13} GC/Animal – Mid-Dose)									
	Study Day	BL	0	7±1	14±2	28±3	60±3	90±4	120±4	150±4	180±5
Complete Blood Counts (Hematology) & Plasma Coagulation	WBC (10^3/µL)	7.97	9.12	6.80	8.67	8.69	11.04	8.43	7.08	5.18	6.06
	RBC (10^6/µL)	5.78	5.77	5.65	5.60	5.80	5.84	5.73	5.06	6.09	5.84
	HB (g/dL)	13.2	13.2	12.8	12.4	13.0	13.2	12.7	11.3	13.8	12.8
	HCT (%)	43.7	44.1	42.8	42.5	43.4	43.4	43.1	39.6	46.3	43.4
	MCV (fL)	75.6	76.4	75.8	75.9	74.8	74.2	75.3	78.4	76.0	74.3
	MCH (pg)	22.9	22.8	22.6	22.1	22.4	22.6	22.2	22.3	22.6	21.9
	MCHC (g/dL)	30.3	29.9	29.9	29.1	29.9	30.4	29.6	28.4	29.7	29.4
	PLT (10^3/µL)	297	250	319	362	291	277	265	323	194	207
	NEU% (%)	29.3	30.9	40.2	23.8	43.8	51.8	54.0	58.8	32.7	45.5
	NEU (10^3/µL)	2.33	2.82	2.74	2.06	3.80	5.72	4.56	4.16	1.70	2.76
	LYMPH% (%)	58.4	59.9	51.2	64.9	49.5	44.4	39.8	35.1	61.1	47.9
	LYMPH (10^3/µL)	4.65	5.46	3.48	5.62	4.30	4.90	3.36	2.48	3.17	2.90
	MONO% (%)	2.7	3.0	1.8	1.5	2.1	0.7	2.2	2.3	2.2	3.2
	MONO (10^3/µL)	0.22	0.27	0.12	0.13	0.18	0.08	0.19	0.16	0.11	0.19
	EOS% (%)	8.0	4.4	5.1	7.9	2.6	1.6	2.7	2.5	2.7	2.5
	EOS (10^3/µL)	0.64	0.40	0.35	0.68	0.23	0.18	0.23	0.18	0.14	0.15
	BAS% (%)	0.5	0.8	0.4	0.5	0.7	0.7	0.5	0.4	0.3	0.4
	BAS (10^3/µL)	0.04	0.08	0.03	0.05	0.06	0.08	0.04	0.03	0.02	0.02
	LUC% (%)	1.1	1.0	1.2	1.4	1.2	0.8	0.7	0.9	1.0	0.6
	LUC (10^3/µL)	0.08	0.09	0.08	0.12	0.11	0.09	0.06	0.06	0.05	0.04
	MAC	+	+	+	+	N/A	N/A	+	+	+	N/A
	LPLT	N/A	N/A	N/A	N/A	N/A	N/A	N/A	N/A	N/A	N/A
	CPLT	N/A	+	N/A	N/A	+	N/A	+	N/A	N/A	N/A
	ANIS	N/A	N/A	N/A	N/A	N/A	N/A	N/A	N/A	N/A	N/A
	ATYP	N/A	N/A	N/A	N/A	N/A	N/A	N/A	N/A	N/A	N/A
	COMMENT	N/A	N/A	N/A	N/A	N/A	N/A	N/A	N/A	N/A	N/A
	PT (sec)	11.4	10.9	10.3	10.5	11.2	10.8	10.9	10.7	10.9	10.4
	APTT (sec)	21.9	22.3	20.9	22.1	22.1	21.3	21.6	20.6	21.3	21.5
	FIB (mg/dL)	172	181	211	201	169	181	189	216	158	155
	DIME (ng/mL)	235.8	210.2	228.3	312.5	330.7	379.8	227.9	1523.	173.5	161.9
	FDP (FEU)	<2.5	<2.5	<2.5	<2.5	<2.5	<2.5	<2.5	<2.5	<2.5	<2.5

	Group (Necropsy Day)	Group 7 (Necropsy Day 180±5)									
	Animal ID (Sex)	18-181 (Female)									
	Treatment (Dose)	GTP-206 (1.5 x 10^{13} GC/Animal – Mid-Dose)									
	Study Day	BL	0	7±1	14±2	28±3	60±3	90±4	120±4	150±4	180±5
Serum Chemistry	BUN (mg/dL)	11	23	20	13	26	22	19	23	20	17
	CREA (mg/dL)	0.6	0.5	0.5	0.5	0.4	0.5	0.5	0.5	0.6	0.6
	GLU (mg/dL)	96	96	92	88	81	49	46	111	113	117
	NA (mmol/L)	155	147	148	148	147	148	149	150	151	147
	K (mmol/L)	3.8	3.2	3.5	3.5	3.4	3.1	3.3	3.5	3.6	3.4
	CL (mmol/L)	110	105	108	107	106	108	108	109	112	108
	ALP (U/L)	877	808	670	763	684	627	455	428	645	619
	ALT (U/L)	23	24	30	57	30	35	29	39	32	25
	AST (U/L)	27	21	25	45	29	30	26	49	22	23
	TBIL (mg/dL)	0.2	0.1	0.1	0.1	0.1	0.2	0.2	0.2	0.1	0.2
	LDH (U/L)	356	231	261	494	306	308	275	539	243	257
	CPK (U/L)	282	165	194	426	389	396	125	2690	222	243
	GGT (U/L)	68	63	65	65	67	61	53	49	64	60
	TPRO (g/dL)	6.0	6.1	6.3	5.9	5.7	6.2	6.2	6.4	6.9	6.3
	ALB (g/dL)	3.9	4.0	4.1	3.7	3.8	3.8	4.0	3.7	4.5	4.1
	GLOB (g/dL)	2.1	2.1	2.2	2.2	1.9	2.4	2.2	2.7	2.4	2.2
	A/G	1.86	1.90	1.86	1.68	2.00	1.58	1.82	1.37	1.88	1.86
	CA (mg/dL)	10.0	9.4	9.7	9.4	9.2	9.0	8.9	9.2	10.1	9.5
	PHOS (mg/dL)	6.3	6.3	5.1	5.9	5.5	5.6	6.5	5.8	4.6	4.9
	ALD (U/L)	23.6	18.7	17.9	33.5	20.6	18.8	22.8	51.5	16.0	14.4
	CKMM (U/L)	QNS	67	108	312	269	275	72	2566	151	188
	CKM% (%)	QNS	40.8	55.9	73.2	69.2	69.4	57.3	95.4	68.0	77.3
	CKMB (U/L)	QNS	39	40	43	53	55	23	70	33	30
	CMB% (%)	QNS	23.6	20.8	10.0	13.6	14.0	18.2	2.6	15.0	12.2
	CKBB (U/L)	QNS	59	45	72	67	66	31	51	38	26
	CKB% (%)	QNS	35.6	23.4	16.8	17.2	16.6	24.5	1.9	17.0	10.5
	COMMENT	N/A	N/A	N/A	N/A	N/A	N/A	N/A	N/A	N/A	N/A

	Group (Necropsy Day)	Group 7 (Necropsy Day 180±5)									
	Animal ID (Sex)	18-183 (Female)									
	Treatment (Dose)	GTP-206 (1.5 x 10^{13} GC/Animal – Mid-Dose)									
	**Study Day**	**BL**	**0**	**7±1**	**14±2**	**28±3**	**60±3**	**90±4**	**120±4**	**150±4**	**180±5**
**Complete Blood Counts (Hematology) & Plasma Coagulation**	**WBC (10^3/µL)**	10.52	9.72	12.40	11.29	10.47	10.99	9.93	10.44	9.09	10.89
	**RBC (10^6/µL)**	5.40	5.45	5.10	5.37	5.44	5.54	5.29	5.52	5.37	5.29
	**HB (g/dL)**	12.5	12.5	11.7	12.0	12.6	12.3	12.1	12.2	12.2	11.9
	**HCT (%)**	41.0	41.8	39.5	41.3	41.2	41.8	40.2	41.9	41.0	40.2
	**MCV (fL)**	76.1	76.7	77.4	77.0	75.7	75.3	76.0	75.9	76.3	75.9
	**MCH (pg)**	23.2	22.9	22.9	22.3	23.1	22.2	22.8	22.1	22.7	22.6
	**MCHC (g/dL)**	30.5	29.9	29.6	29.0	30.5	29.5	30.0	29.2	29.8	29.7
	**PLT (10^3/µL)**	482	415	581	573	492	403	452	572	487	499
	**NEU% (%)**	49.6	47.6	64.2	48.0	66.7	63.0	66.8	41.5	56.7	67.2
	**NEU (10^3/µL)**	5.22	4.63	7.97	5.41	6.98	6.92	6.64	4.34	5.15	7.31
	**LYMPH% (%)**	45.8	42.9	32.6	48.1	25.9	31.5	27.2	54.6	40.1	30.1
	**LYMPH (10^3/µL)**	4.82	4.17	4.04	5.43	2.71	3.47	2.70	5.70	3.65	3.27
	**MONO% (%)**	2.6	6.7	1.7	1.3	5.5	3.3	4.2	1.9	2.0	1.8
	**MONO (10^3/µL)**	0.28	0.65	0.21	0.14	0.58	0.37	0.42	0.20	0.18	0.19
	**EOS% (%)**	1.1	0.7	0.6	1.1	0.4	0.5	0.5	0.8	0.5	0.3
	**EOS (10^3/µL)**	0.11	0.07	0.07	0.13	0.04	0.05	0.05	0.08	0.05	0.04
	**BAS% (%)**	0.4	1.3	0.4	0.7	0.8	1.0	0.8	0.6	0.3	0.3
	**BAS (10^3/µL)**	0.04	0.12	0.04	0.08	0.08	0.11	0.08	0.06	0.03	0.03
	**LUC% (%)**	0.5	0.7	0.6	0.9	0.7	0.7	0.5	0.6	0.4	0.3
	**LUC (10^3/µL)**	0.05	0.07	0.07	0.10	0.08	0.07	0.05	0.07	0.04	0.04
	**MAC**	+	+	+	+	+	+	+	+	+	+
	**LPLT**	N/A	N/A	N/A	N/A	N/A	N/A	N/A	N/A	N/A	N/A
	**CPLT**	N/A	+	N/A	N/A	+	+	+	N/A	N/A	N/A
	**ANIS**	N/A	N/A	N/A	N/A	N/A	N/A	N/A	N/A	N/A	N/A
	**ATYP**	N/A	N/A	N/A	N/A	N/A	N/A	N/A	N/A	N/A	N/A
	**COMMENT**	HEM	HEM	N/A	HEM	HEM	N/A	N/A	N/A	N/A	N/A
	**PT (sec)**	11.1	10.8	10.3	10.4	10.1	10.8	11.6	10.5	11.0	9.9
	**APTT (sec)**	22.3	22.1	21.8	21.8	21.2	23.4	23.3	21.2	21.9	21.1
	**FIB (mg/dL)**	158	174	218	189	141	193	183	183	161	161
	**DIME (ng/mL)**	283.6	552.8	463.4	291.8	1477.	506.4	297.1	417.2	300.6	605.0
	**FDP (FEU)**	<2.5	<2.5	<2.5	<2.5	<2.5	2.5	<2.5	<2.5	<2.5	<2.5

	Group (Necropsy Day)	Group 7 (Necropsy Day 180±5)									
	Animal ID (Sex)	18-183 (Female)									
	Treatment (Dose)	GTP-206 (1.5 x 10^{13} GC/Animal – Mid-Dose)									
	Study Day	BL	0	7±1	14±2	28±3	60±3	90±4	120±4	150±4	180±5
Serum Chemistry	BUN (mg/dL)	QNS	21	20	15	20	21	21	22	23	21
	CREA (mg/dL)	0.5	0.4	0.4	0.4	0.4	0.4	0.4	0.4	0.5	0.5
	GLU (mg/dL)	QNS	103	134	89	75	67	99	104	106	116
	NA (mmol/L)	154	146	145	145	145	145	146	147	154	147
	K (mmol/L)	4.0	3.5	3.6	4.0	3.9	3.3	3.5	3.5	3.8	3.5
	CL (mmol/L)	109	106	108	104	106	105	106	107	112	108
	ALP (U/L)	830	908	734	1011	939	867	722	840	915	789
	ALT (U/L)	33	31	35	39	40	41	30	34	31	28
	AST (U/L)	38	28	28	39	37	35	24	29	33	25
	TBIL (mg/dL)	0.2	0.2	0.1	0.2	0.2	0.2	0.2	0.2	0.2	0.2
	LDH (U/L)	QNS	279	326	590	329	344	270	319	375	304
	CPK (U/L)	729	227	214	255	231	441	214	258	791	416
	GGT (U/L)	62	68	70	69	74	70	65	69	73	62
	TPRO (g/dL)	QNS	6.5	6.4	6.1	6.3	6.3	6.3	6.1	6.7	6.2
	ALB (g/dL)	4.2	4.4	4.1	4.0	4.3	4.0	4.3	4.1	4.7	4.3
	GLOB (g/dL)	QNS	2.1	2.3	2.1	2.0	2.3	2.0	2.0	2.0	1.9
	A/G	QNS	2.10	1.78	1.90	2.15	1.74	2.15	2.05	2.35	2.26
	CA (mg/dL)	9.9	9.6	9.4	9.5	9.6	9.3	9.4	9.8	10.5	9.5
	PHOS (mg/dL)	5.4	5.8	5.9	6.8	5.1	6.4	5.4	5.8	6.3	5.7
	ALD (U/L)	QNS	17.9	21.2	30.1	21.7	21.3	17.7	21.6	21.6	19.7
	CKMM (U/L)	QNS	129	136	155	142	331	155	190	665	301
	CKM% (%)	QNS	56.8	63.4	60.9	61.3	75.1	72.5	73.7	84.1	72.3
	CKMB (U/L)	QNS	37	38	27	36	42	24	23	51	68
	CMB% (%)	QNS	16.2	17.9	10.5	15.6	9.6	11.2	8.9	6.5	16.3
	CKBB (U/L)	QNS	61	40	73	53	67	35	45	74	47
	CKB% (%)	QNS	27.0	18.7	28.6	23.1	15.3	16.2	17.4	9.4	11.3
	COMMENT	N/A	N/A	N/A	N/A	N/A	N/A	N/A	N/A	N/A	N/A

	Group (Necropsy Day)	Group 4 (Necropsy Day 90±4)									
	Animal ID (Sex)	18-080 (Male)									
	Treatment (Dose)	GTP-206 (4.5 x 10^{13} GC/Animal – High Dose)									
	**Study Day**	**BL**	**0**	**7±1**	**14±2**	**28±3**	**60±3**	**90±4**	**120±4**	**150±4**	**180±5**
**Complete Blood Counts (Hematology) & Plasma Coagulation**	**WBC (10^3/µL)**	7.08	11.30	9.99	12.20	11.31	8.30	7.32	N/A	N/A	N/A
	**RBC (10^6/µL)**	6.00	5.58	5.88	5.95	5.88	6.05	5.90	N/A	N/A	N/A
	**HB (g/dL)**	13.1	12.5	13.1	12.9	12.7	13.4	12.8	N/A	N/A	N/A
	**HCT (%)**	43.8	40.2	42.7	43.0	41.9	43.7	41.8	N/A	N/A	N/A
	**MCV (fL)**	73.0	72.1	72.5	72.2	71.3	72.1	70.9	N/A	N/A	N/A
	**MCH (pg)**	21.9	22.4	22.2	21.8	21.7	22.2	21.8	N/A	N/A	N/A
	**MCHC (g/dL)**	30.0	31.1	30.7	30.1	30.4	30.7	30.7	N/A	N/A	N/A
	**PLT (10^3/µL)**	75	557	513	461	446	516	492	N/A	N/A	N/A
	**NEU% (%)**	58.8	46.3	46.9	48.1	72.7	43.7	55.2	N/A	N/A	N/A
	**NEU (10^3/µL)**	4.16	5.23	4.69	5.86	8.22	3.63	4.04	N/A	N/A	N/A
	**LYMPH% (%)**	37.6	47.9	49.5	46.5	25.0	51.3	41.5	N/A	N/A	N/A
	**LYMPH (10^3/µL)**	2.66	5.41	4.95	5.67	2.83	4.26	3.04	N/A	N/A	N/A
	**MONO% (%)**	1.4	2.3	1.2	2.0	0.6	2.1	1.1	N/A	N/A	N/A
	**MONO (10^3/µL)**	0.10	0.26	0.12	0.24	0.07	0.17	0.08	N/A	N/A	N/A
	**EOS% (%)**	1.0	1.2	0.5	1.9	0.5	1.2	0.6	N/A	N/A	N/A
	**EOS (10^3/µL)**	0.07	0.14	0.05	0.23	0.05	0.10	0.04	N/A	N/A	N/A
	**BAS% (%)**	0.5	0.7	0.8	0.8	0.8	0.4	0.4	N/A	N/A	N/A
	**BAS (10^3/µL)**	0.03	0.07	0.08	0.09	0.09	0.03	0.03	N/A	N/A	N/A
	**LUC% (%)**	0.7	1.6	1.0	0.9	0.4	1.3	1.2	N/A	N/A	N/A
	**LUC (10^3/µL)**	0.05	0.18	0.10	0.11	0.04	0.10	0.09	N/A	N/A	N/A
	**MAC**	N/A	N/A	N/A	N/A	N/A	N/A	N/A	N/A	N/A	N/A
	**LPLT**	N/A	N/A	N/A	N/A	N/A	N/A	N/A	N/A	N/A	N/A
	**CPLT**	N/A	N/A	N/A	N/A	N/A	N/A	N/A	N/A	N/A	N/A
	**ANIS**	N/A	N/A	N/A	N/A	N/A	N/A	N/A	N/A	N/A	N/A
	**ATYP**	N/A	N/A	N/A	N/A	N/A	N/A	N/A	N/A	N/A	N/A
	**COMMENT**	N/A	N/A	HEM	N/A	N/A	N/A	N/A	N/A	N/A	N/A
	**PT (sec)**	10.3	10.2	10.0	10.3	9.9	10.7	10.6	N/A	N/A	N/A
	**APTT (sec)**	19.9	20.3	20.7	22.7	20.8	20.7	20.7	N/A	N/A	N/A
	**FIB (mg/dL)**	171	195	244	179	224	149	143	N/A	N/A	N/A
	**DIME (ng/mL)**	447.3	389.9	426.8	417.5	2231.	360.0	398.2	N/A	N/A	N/A
	**FDP (FEU)**	<2.5	<2.5	<2.5	<2.5	5.0	<2.5	<2.5	N/A	N/A	N/A

	Group (Necropsy Day)	Group 4 (Necropsy Day 90±4)									
	Animal ID (Sex)	18-080 (Male)									
	Treatment (Dose)	GTP-206 (4.5 x 10^{13} GC/Animal – High Dose)									
	Study Day	BL	0	7±1	14±2	28±3	60±3	90±4	120±4	150±4	180±5
Serum Chemistry	BUN (mg/dL)	19	19	19	17	17	20	18	N/A	N/A	N/A
	CREA (mg/dL)	0.5	0.5	0.5	0.5	0.5	0.4	0.4	N/A	N/A	N/A
	GLU (mg/dL)	99	79	107	99	90	74	106	N/A	N/A	N/A
	NA (mmol/L)	149	147	147	147	147	146	152	N/A	N/A	N/A
	K (mmol/L)	3.1	3.6	3.3	3.2	3.1	3.4	2.9	N/A	N/A	N/A
	CL (mmol/L)	108	108	108	105	108	106	110	N/A	N/A	N/A
	ALP (U/L)	804	629	683	684	718	959	912	N/A	N/A	N/A
	ALT (U/L)	28	25	51	50	98	35	35	N/A	N/A	N/A
	AST (U/L)	27	38	28	47	80	32	28	N/A	N/A	N/A
	TBIL (mg/dL)	0.2	0.2	0.2	0.2	0.1	0.2	0.2	N/A	N/A	N/A
	LDH (U/L)	227	304	231	298	391	210	198	N/A	N/A	N/A
	CPK (U/L)	122	1443	129	270	316	153	160	N/A	N/A	N/A
	GGT (U/L)	62	57	60	60	64	71	62	N/A	N/A	N/A
	TPRO (g/dL)	6.5	6.1	6.5	6.3	6.6	6.5	5.9	N/A	N/A	N/A
	ALB (g/dL)	4.0	4.0	3.9	3.9	3.9	4.1	3.8	N/A	N/A	N/A
	GLOB (g/dL)	2.5	2.1	2.6	2.4	2.7	2.4	2.1	N/A	N/A	N/A
	A/G	1.60	1.90	1.50	1.63	1.44	1.71	1.81	N/A	N/A	N/A
	CA (mg/dL)	10.0	10.2	10.2	10.3	9.8	9.8	9.4	N/A	N/A	N/A
	PHOS (mg/dL)	5.4	6.2	5.7	5.7	4.8	6.5	6.3	N/A	N/A	N/A
	ALD (U/L)	12.4	28.1	12.0	16.8	53.6	12.2	13.8	N/A	N/A	N/A
	CKMM (U/L)	76	1234	75	180	270	79	88	N/A	N/A	N/A
	CKM% (%)	62.4	85.5	57.8	66.8	85.4	51.8	54.8	N/A	N/A	N/A
	CKMB (U/L)	26	84	29	45	22	39	38	N/A	N/A	N/A
	CMB% (%)	21.0	5.8	22.6	16.5	6.9	25.7	23.7	N/A	N/A	N/A
	CKBB (U/L)	20	126	25	45	24	34	34	N/A	N/A	N/A
	CKB% (%)	16.7	8.7	19.7	16.7	7.7	22.5	21.5	N/A	N/A	N/A
	COMMENT	N/A	N/A	N/A	N/A	N/A	N/A	N/A	N/A	N/A	N/A

	Group (Necropsy Day)	Group 4 (Necropsy Day 90±4)									
	Animal ID (Sex)	18-166 (Male)									
	Treatment (Dose)	GTP-206 (4.5 x 10^{13} GC/Animal – High Dose)									
	Study Day	BL	0	7±1	14±2	28±3	60±3	90±4	120±4	150±4	180±5
Complete Blood Counts (Hematology) & Plasma Coagulation	WBC (10^3/µL)	10.34	11.73	14.18	12.60	12.37	13.48	12.72	N/A	N/A	N/A
	RBC (10^6/µL)	5.90	5.37	5.22	4.78	5.97	6.00	5.75	N/A	N/A	N/A
	HB (g/dL)	13.3	11.7	11.2	10.7	12.9	12.8	12.5	N/A	N/A	N/A
	HCT (%)	43.2	41.4	38.6	36.2	44.8	43.5	42.1	N/A	N/A	N/A
	MCV (fL)	73.3	77.1	74.1	75.8	75.1	72.4	73.3	N/A	N/A	N/A
	MCH (pg)	22.6	21.8	21.4	22.4	21.6	21.4	21.7	N/A	N/A	N/A
	MCHC (g/dL)	30.8	28.3	28.9	29.6	28.8	29.5	29.6	N/A	N/A	N/A
	PLT (10^3/µL)	432	283	305	447	364	387	315	N/A	N/A	N/A
	NEU% (%)	16.6	27.4	51.2	38.6	69.7	70.0	65.3	N/A	N/A	N/A
	NEU (10^3/µL)	1.71	3.21	7.26	4.86	8.62	9.44	8.31	N/A	N/A	N/A
	LYMPH% (%)	73.6	63.9	40.2	56.3	25.0	25.7	26.6	N/A	N/A	N/A
	LYMPH (10^3/µL)	7.61	7.50	5.70	7.09	3.09	3.47	3.38	N/A	N/A	N/A
	MONO% (%)	4.3	4.7	5.4	1.5	2.1	1.7	4.6	N/A	N/A	N/A
	MONO (10^3/µL)	0.44	0.55	0.77	0.19	0.26	0.23	0.59	N/A	N/A	N/A
	EOS% (%)	3.2	2.2	1.0	1.5	1.2	1.2	1.9	N/A	N/A	N/A
	EOS (10^3/µL)	0.34	0.26	0.14	0.19	0.15	0.17	0.24	N/A	N/A	N/A
	BAS% (%)	0.8	0.7	1.1	1.0	1.3	0.8	1.1	N/A	N/A	N/A
	BAS (10^3/µL)	0.09	0.08	0.16	0.13	0.16	0.11	0.14	N/A	N/A	N/A
	LUC% (%)	1.5	1.1	1.1	1.1	0.6	0.5	0.6	N/A	N/A	N/A
	LUC (10^3/µL)	0.15	0.13	0.16	0.14	0.07	0.07	0.07	N/A	N/A	N/A
	MAC	N/A	+	N/A	+	+	N/A	N/A	N/A	N/A	N/A
	LPLT	N/A	N/A	N/A	N/A	N/A	N/A	N/A	N/A	N/A	N/A
	CPLT	+	+	+	N/A	+	+	+	N/A	N/A	N/A
	ANIS	N/A	N/A	N/A	+	N/A	N/A	+	N/A	N/A	N/A
	ATYP	N/A	N/A	N/A	N/A	N/A	N/A	N/A	N/A	N/A	N/A
	COMMENT	N/A	N/A	TRB	N/A	N/A	N/A	N/A	N/A	N/A	N/A
	PT (sec)	11.3	10.9	9.7	10.3	10.8	10.4	10.7	N/A	N/A	N/A
	APTT (sec)	20.5	21.9	21.6	20.7	22.2	20.3	20.8	N/A	N/A	N/A
	FIB (mg/dL)	142	138	398	161	136	142	129	N/A	N/A	N/A
	DIME (ng/mL)	256.7	222.4	2579.	2274.	466.9	220.0	543.9	N/A	N/A	N/A
	FDP (FEU)	<2.5	<2.5	10.0	5.0	<2.5	<2.5	<2.5	N/A	N/A	N/A

	Group (Necropsy Day)	Group 4 (Necropsy Day 90±4)									
	Animal ID (Sex)	18-166 (Male)									
	Treatment (Dose)	GTP-206 (4.5 x 10^{13} GC/Animal – High Dose)									
	Study Day	BL	0	7±1	14±2	28±3	60±3	90±4	120±4	150±4	180±5
Serum Chemistry	BUN (mg/dL)	27	22	17	22	24	27	18	N/A	N/A	N/A
	CREA (mg/dL)	0.5	0.5	0.5	0.5	0.5	0.5	0.4	N/A	N/A	N/A
	GLU (mg/dL)	93	111	82	74	74	90	148	N/A	N/A	N/A
	NA (mmol/L)	147	145	140	146	147	147	148	N/A	N/A	N/A
	K (mmol/L)	4.1	3.7	5.3	3.3	3.9	3.5	3.9	N/A	N/A	N/A
	CL (mmol/L)	104	104	102	104	105	108	105	N/A	N/A	N/A
	ALP (U/L)	681	642	343	572	717	724	852	N/A	N/A	N/A
	ALT (U/L)	33	25	97	67	24	27	31	N/A	N/A	N/A
	AST (U/L)	62	34	157	36	32	28	40	N/A	N/A	N/A
	TBIL (mg/dL)	0.2	0.1	0.2	0.1	0.2	0.1	0.1	N/A	N/A	N/A
	LDH (U/L)	591	367	758	311	273	210	341	N/A	N/A	N/A
	CPK (U/L)	3964	514	3266	150	451	181	1906	N/A	N/A	N/A
	GGT (U/L)	62	55	34	55	64	58	70	N/A	N/A	N/A
	TPRO (g/dL)	5.6	5.7	6.1	5.9	6.3	6.1	5.7	N/A	N/A	N/A
	ALB (g/dL)	4.2	4.0	3.3	3.5	4.2	4.1	4.0	N/A	N/A	N/A
	GLOB (g/dL)	1.4	1.7	2.8	2.4	2.1	2.0	1.7	N/A	N/A	N/A
	A/G	3.00	2.35	1.18	1.46	2.00	2.05	2.35	N/A	N/A	N/A
	CA (mg/dL)	10.1	9.7	9.4	9.3	10.2	9.9	10.0	N/A	N/A	N/A
	PHOS (mg/dL)	6.2	5.1	5.2	5.9	5.9	4.8	5.8	N/A	N/A	N/A
	ALD (U/L)	78.5	30.9	133.3	46.9	24.3	21.0	48.2	N/A	N/A	N/A
	CKMM (U/L)	3615	419	3050	98	348	116	1618	N/A	N/A	N/A
	CKM% (%)	91.2	81.6	93.4	65.6	77.2	64.1	84.9	N/A	N/A	N/A
	CKMB (U/L)	174	45	108	26	50	39	93	N/A	N/A	N/A
	CMB% (%)	4.4	8.8	3.3	17.2	11.1	21.4	4.9	N/A	N/A	N/A
	CKBB (U/L)	170	49	108	26	53	26	194	N/A	N/A	N/A
	CKB% (%)	4.3	9.6	3.3	17.2	11.8	14.5	10.2	N/A	N/A	N/A
	COMMENT	N/A	N/A	HEM	N/A	N/A	N/A	N/A	N/A	N/A	N/A

	Group (Necropsy Day)	Group 4 (Necropsy Day 90±4)									
	Animal ID (Sex)	18-185 (Female)									
	Treatment (Dose)	GTP-206 (4.5 x 10^{13} GC/Animal – High Dose)									
	Study Day	BL	0	7±1	14±2	28±3	60±3	90±4	120±4	150±4	180±5
Complete Blood Counts (Hematology) & Plasma Coagulation	WBC (10^3/µL)	14.85	17.18	21.40	15.67	12.04	11.90	9.77	N/A	N/A	N/A
	RBC (10^6/µL)	5.74	5.21	5.07	4.52	5.50	5.77	5.50	N/A	N/A	N/A
	HB (g/dL)	12.7	11.5	11.1	10.1	12.0	12.6	11.8	N/A	N/A	N/A
	HCT (%)	43.0	39.9	37.5	34.2	41.7	43.2	40.4	N/A	N/A	N/A
	MCV (fL)	74.9	76.7	74.0	75.7	75.8	74.9	73.5	N/A	N/A	N/A
	MCH (pg)	22.2	22.0	21.9	22.5	21.9	21.9	21.5	N/A	N/A	N/A
	MCHC (g/dL)	29.7	28.7	29.6	29.7	28.9	29.2	29.2	N/A	N/A	N/A
	PLT (10^3/µL)	339	299	231	329	283	310	318	N/A	N/A	N/A
	NEU% (%)	16.9	63.3	55.4	59.5	66.5	42.3	43.3	N/A	N/A	N/A
	NEU (10^3/µL)	2.51	10.88	11.85	9.32	8.00	5.03	4.23	N/A	N/A	N/A
	LYMPH% (%)	62.6	31.4	36.2	36.0	28.3	53.7	51.9	N/A	N/A	N/A
	LYMPH (10^3/µL)	9.30	5.40	7.74	5.64	3.41	6.39	5.07	N/A	N/A	N/A
	MONO% (%)	7.3	2.4	4.6	1.2	1.5	1.0	1.1	N/A	N/A	N/A
	MONO (10^3/µL)	1.09	0.41	0.99	0.19	0.18	0.12	0.10	N/A	N/A	N/A
	EOS% (%)	4.4	1.0	1.1	1.3	2.3	1.0	1.8	N/A	N/A	N/A
	EOS (10^3/µL)	0.65	0.17	0.23	0.21	0.28	0.12	0.18	N/A	N/A	N/A
	BAS% (%)	1.3	0.7	1.5	0.7	0.5	0.7	0.6	N/A	N/A	N/A
	BAS (10^3/µL)	0.19	0.11	0.32	0.11	0.06	0.08	0.06	N/A	N/A	N/A
	LUC% (%)	7.5	1.2	1.2	1.3	1.0	1.3	1.3	N/A	N/A	N/A
	LUC (10^3/µL)	1.11	0.21	0.26	0.20	0.12	0.16	0.13	N/A	N/A	N/A
	MAC	+	+	N/A	+	+	+	N/A	N/A	N/A	N/A
	LPLT	N/A	N/A	+	N/A	N/A	N/A	N/A	N/A	N/A	N/A
	CPLT	N/A	N/A	+	N/A	N/A	N/A	N/A	N/A	N/A	N/A
	ANIS	N/A	N/A	N/A	+	N/A	N/A	N/A	N/A	N/A	N/A
	ATYP	+	N/A	N/A	N/A	N/A	N/A	N/A	N/A	N/A	N/A
	COMMENT	N/A	N/A	TRB	N/A	N/A	N/A	N/A	N/A	N/A	N/A
	PT (sec)	10.7	10.1	9.8	10.0	10.7	10.9	10.6	N/A	N/A	N/A
	APTT (sec)	25.6	26.2	30.6	22.9	26.7	25.8	23.5	N/A	N/A	N/A
	FIB (mg/dL)	213	201	425	211	142	140	137	N/A	N/A	N/A
	DIME (ng/mL)	346.9	305.2	7524.	1929.	347.5	298.8	464.3	N/A	N/A	N/A
	FDP (FEU)	<2.5	<2.5	20.0	5.0	<2.5	<2.5	<2.5	N/A	N/A	N/A

	Group (Necropsy Day)	Group 4 (Necropsy Day 90±4)									
	Animal ID (Sex)	18-185 (Female)									
	Treatment (Dose)	GTP-206 (4.5 x 10^{13} GC/Animal – High Dose)									
	Study Day	BL	0	7±1	14±2	28±3	60±3	90±4	120±4	150±4	180±5
Serum Chemistry	BUN (mg/dL)	QNS	17	17	23	25	26	18	N/A	N/A	N/A
	CREA (mg/dL)	0.4	0.5	0.4	0.4	0.4	0.4	0.3	N/A	N/A	N/A
	GLU (mg/dL)	QNS	83	94	89	74	73	116	N/A	N/A	N/A
	NA (mmol/L)	148	144	143	145	144	144	147	N/A	N/A	N/A
	K (mmol/L)	3.8	3.9	3.7	3.7	3.2	3.4	3.5	N/A	N/A	N/A
	CL (mmol/L)	108	106	99	106	105	105	109	N/A	N/A	N/A
	ALP (U/L)	563	607	337	425	654	698	776	N/A	N/A	N/A
	ALT (U/L)	30	30	106	76	34	37	25	N/A	N/A	N/A
	AST (U/L)	35	72	192	54	34	33	26	N/A	N/A	N/A
	TBIL (mg/dL)	0.2	0.2	0.1	0.1	0.2	0.1	0.1	N/A	N/A	N/A
	LDH (U/L)	303	697	624	375	205	233	321	N/A	N/A	N/A
	CPK (U/L)	594	4822	1927	1230	152	140	214	N/A	N/A	N/A
	GGT (U/L)	51	50	31	41	49	59	58	N/A	N/A	N/A
	TPRO (g/dL)	5.5	6.1	5.8	6.4	5.9	5.8	5.8	N/A	N/A	N/A
	ALB (g/dL)	3.8	3.8	2.7	3.3	3.5	3.6	3.7	N/A	N/A	N/A
	GLOB (g/dL)	1.7	2.3	3.1	3.1	2.4	2.2	2.1	N/A	N/A	N/A
	A/G	2.24	1.65	0.87	1.06	1.46	1.64	1.76	N/A	N/A	N/A
	CA (mg/dL)	9.2	9.2	8.6	9.0	8.7	8.6	8.9	N/A	N/A	N/A
	PHOS (mg/dL)	7.1	6.8	4.5	6.1	7.4	6.5	7.2	N/A	N/A	N/A
	ALD (U/L)	QNS	83.0	165.4	51.8	20.1	21.5	22.4	N/A	N/A	N/A
	CKMM (U/L)	QNS	4518	1821	1100	73	50	132	N/A	N/A	N/A
	CKM% (%)	QNS	93.7	94.5	89.4	47.9	35.5	61.8	N/A	N/A	N/A
	CKMB (U/L)	QNS	174	54	59	38	55	27	N/A	N/A	N/A
	CMB% (%)	QNS	3.6	2.8	4.8	25.3	39.4	12.8	N/A	N/A	N/A
	CKBB (U/L)	QNS	130	52	73	41	35	54	N/A	N/A	N/A
	CKB% (%)	QNS	2.7	2.7	5.9	26.8	25.1	25.4	N/A	N/A	N/A
	COMMENT	N/A	N/A	N/A	N/A	N/A	N/A	N/A	N/A	N/A	N/A

	Group (Necropsy Day)	Group 8 (Necropsy Day 180±5)									
	Animal ID (Sex)	18-038 (Female)									
	Treatment (Dose)	GTP-206 (4.5 x 10^{13} GC/Animal – High Dose)									
	Study Day	BL	0	7±1	14±2	28±3	60±3	90±4	120±4	150±4	180±5
Complete Blood Counts (Hematology) & Plasma Coagulation	WBC (10^3/µL)	9.68	7.48	7.76	9.39	9.29	10.70	CLOT	10.14	9.88	9.39
	RBC (10^6/µL)	5.51	5.61	5.70	5.80	5.76	6.03	CLOT	5.56	5.70	5.25
	HB (g/dL)	12.5	12.5	12.6	12.7	12.8	13.2	CLOT	11.9	12.6	11.3
	HCT (%)	39.6	40.7	41.7	42.2	42.5	43.3	CLOT	40.6	41.3	37.6
	MCV (fL)	71.8	72.5	73.2	72.7	73.7	71.7	CLOT	73.1	72.5	71.6
	MCH (pg)	22.7	22.2	22.1	21.8	22.1	21.8	CLOT	21.4	22.1	21.6
	MCHC (g/dL)	31.7	30.7	30.2	30.0	30.0	30.4	CLOT	29.2	30.4	30.1
	PLT (10^3/µL)	377	177	394	372	156	362	CLOT	226	396	421
	NEU% (%)	64.3	53.3	46.3	49.4	57.2	31.9	CLOT	63.5	40.9	36.7
	NEU (10^3/µL)	6.22	3.99	3.60	4.64	5.32	3.42	CLOT	6.44	4.04	3.45
	LYMPH% (%)	31.6	40.1	45.5	42.0	37.3	60.8	CLOT	32.9	53.0	57.2
	LYMPH (10^3/µL)	3.05	3.00	3.53	3.94	3.47	6.51	CLOT	3.33	5.24	5.37
	MONO% (%)	2.5	1.7	3.2	2.0	2.1	2.4	CLOT	1.2	1.4	1.9
	MONO (10^3/µL)	0.24	0.12	0.25	0.19	0.19	0.26	CLOT	0.13	0.14	0.18
	EOS% (%)	0.9	2.6	2.2	4.4	1.0	2.0	CLOT	1.5	3.1	2.3
	EOS (10^3/µL)	0.09	0.19	0.17	0.41	0.09	0.22	CLOT	0.15	0.31	0.22
	BAS% (%)	0.3	1.0	0.5	0.5	0.8	1.1	CLOT	0.3	0.4	0.7
	BAS (10^3/µL)	0.03	0.08	0.04	0.04	0.08	0.12	CLOT	0.03	0.04	0.07
	LUC% (%)	0.5	1.2	2.3	1.8	1.6	1.7	CLOT	0.5	1.1	1.1
	LUC (10^3/µL)	0.05	0.09	0.18	0.17	0.15	0.18	CLOT	0.06	0.11	0.10
	MAC	N/A	N/A	N/A	N/A	N/A	N/A	N/A	N/A	N/A	N/A
	LPLT	N/A	N/A	N/A	N/A	N/A	N/A	N/A	N/A	N/A	N/A
	CPLT	N/A	N/A	N/A	N/A	N/A	N/A	N/A	N/A	N/A	N/A
	ANIS	N/A	N/A	N/A	N/A	N/A	N/A	N/A	N/A	N/A	N/A
	ATYP	N/A	N/A	N/A	N/A	N/A	N/A	N/A	N/A	N/A	N/A
	COMMENT	HEM	N/A	HEM	N/A	HEM	HEM	N/A	N/A	N/A	N/A
	PT (sec)	10.6	10.6	9.9	9.9	10.6	10.7	10.9	10.5	10.4	10.1
	APTT (sec)	20.3	22.0	21.1	21.5	21.1	21.6	21.6	21.5	20.7	21.9
	FIB (mg/dL)	187	179	258	265	174	172	164	138	191	191
	DIME (ng/mL)	269.7	374.2	533.2	691.2	552.1	327.2	345.2	1250.	368.5	450.9
	FDP (FEU)	<2.5	<2.5	<2.5	2.5	<2.5	<2.5	<2.5	10.0	<2.5	<2.5

	Group (Necropsy Day)	Group 8 (Necropsy Day 180±5)									
	Animal ID (Sex)	18-038 (Female)									
	Treatment (Dose)	GTP-206 (4.5 x 10^{13} GC/Animal – High Dose)									
	Study Day	BL	0	7±1	14±2	28±3	60±3	90±4	120±4	150±4	180±5
Serum Chemistry	BUN (mg/dL)	27	26	22	19	19	23	22	19	17	17
	CREA (mg/dL)	0.6	0.4	0.3	0.4	0.3	0.4	0.3	0.4	0.4	0.3
	GLU (mg/dL)	103	71	84	107	131	71	95	90	86	71
	NA (mmol/L)	155	149	149	148	145	146	146	149	146	146
	K (mmol/L)	3.2	3.3	3.1	3.2	3.5	3.6	3.3	3.6	3.3	3.1
	CL (mmol/L)	111	109	111	110	109	106	107	109	107	107
	ALP (U/L)	494	492	382	489	486	517	444	527	608	534
	ALT (U/L)	31	29	34	38	42	39	34	31	27	19
	AST (U/L)	29	34	32	41	40	34	31	38	24	21
	TBIL (mg/dL)	0.1	0.2	0.1	0.1	0.1	0.2	0.2	0.2	0.1	0.2
	LDH (U/L)	286	352	293	343	324	281	254	319	209	184
	CPK (U/L)	175	348	138	267	420	197	161	552	187	119
	GGT (U/L)	68	70	73	74	78	77	74	74	80	72
	TPRO (g/dL)	6.4	6.1	6.4	6.4	6.4	6.3	6.3	6.1	6.1	6.4
	ALB (g/dL)	3.8	3.7	3.8	3.8	3.8	3.6	3.9	3.6	3.8	3.7
	GLOB (g/dL)	2.6	2.4	2.6	2.6	2.6	2.7	2.4	2.5	2.3	2.7
	A/G	1.46	1.54	1.46	1.46	1.46	1.33	1.63	1.44	1.65	1.37
	CA (mg/dL)	10.1	9.6	9.3	9.6	9.6	9.8	9.7	9.6	10.0	9.7
	PHOS (mg/dL)	4.2	5.2	4.6	4.8	5.1	4.4	4.5	6.7	5.0	5.7
	ALD (U/L)	15.6	19.6	13.2	18.4	20.8	16.8	13.0	18.8	11.2	10.7
	CKMM (U/L)	QNS	261	83	185	329	91	112	487	89	65
	CKM% (%)	QNS	75.1	60.0	69.2	78.3	46.2	69.8	88.2	47.7	54.7
	CKMB (U/L)	QNS	36	30	32	45	45	17	27	39	21
	CMB% (%)	QNS	10.3	21.8	11.9	10.7	22.7	10.7	4.9	20.8	18.0
	CKBB (U/L)	QNS	51	25	50	46	61	31	38	59	32
	CKB% (%)	QNS	14.6	18.1	18.9	11.0	31.1	19.5	6.9	31.5	27.3
	COMMENT	N/A	N/A	N/A	N/A	N/A	N/A	HEM	HEM	N/A	N/A

	Group (Necropsy Day)	Group 8 (Necropsy Day 180±5)									
	Animal ID (Sex)	18-158 (Female)									
	Treatment (Dose)	GTP-206 (4.5 x 10^{13} GC/Animal – High Dose)									
	Study Day	BL	0	7±1	14±2	28±3	60±3	90±4	120±4	150±4	180±5
Complete Blood Counts (Hematology) & Plasma Coagulation	WBC (10^3/µL)	10.31	11.95	9.95	10.80	8.71	9.19	6.51	8.48	9.23	11.40
	RBC (10^6/µL)	5.90	5.90	5.92	5.81	6.13	6.26	6.26	6.20	6.06	6.18
	HB (g/dL)	12.9	12.4	12.6	12.3	12.9	13.1	13.2	13.0	12.9	13.0
	HCT (%)	42.4	41.7	41.8	41.3	43.6	43.9	44.6	44.9	43.1	43.7
	MCV (fL)	71.8	70.7	70.6	71.0	71.1	70.1	71.1	72.4	71.2	70.8
	MCH (pg)	21.8	21.1	21.3	21.1	21.1	21.0	21.0	21.0	21.3	21.1
	MCHC (g/dL)	30.4	29.8	30.1	29.7	29.7	29.9	29.6	29.0	30.0	29.8
	PLT (10^3/µL)	365	371	457	407	145	359	349	245	410	220
	NEU% (%)	61.0	67.8	47.7	46.0	60.6	28.7	34.0	50.7	38.2	55.3
	NEU (10^3/µL)	6.29	8.10	4.75	4.97	5.28	2.64	2.21	4.30	3.52	6.31
	LYMPH% (%)	34.3	27.1	46.2	46.6	33.9	63.9	59.7	43.5	55.9	39.7
	LYMPH (10^3/µL)	3.54	3.24	4.59	5.04	2.95	5.87	3.88	3.69	5.16	4.52
	MONO% (%)	3.1	2.7	3.2	2.3	2.8	4.2	3.4	2.7	2.7	2.2
	MONO (10^3/µL)	0.32	0.32	0.32	0.25	0.25	0.38	0.22	0.23	0.25	0.25
	EOS% (%)	0.6	1.1	0.7	2.5	0.5	1.2	1.2	1.2	1.9	1.2
	EOS (10^3/µL)	0.06	0.13	0.07	0.27	0.04	0.11	0.08	0.10	0.17	0.14
	BAS% (%)	0.3	0.7	0.5	0.9	0.8	0.7	0.6	1.0	0.4	1.0
	BAS (10^3/µL)	0.03	0.08	0.05	0.10	0.07	0.06	0.04	0.08	0.04	0.11
	LUC% (%)	0.7	0.6	1.6	1.7	1.5	1.4	1.2	0.8	0.9	0.6
	LUC (10^3/µL)	0.07	0.07	0.16	0.18	0.13	0.13	0.08	0.07	0.08	0.07
	MAC	N/A	N/A	N/A	N/A	N/A	N/A	N/A	N/A	N/A	N/A
	LPLT	N/A	N/A	N/A	N/A	N/A	N/A	N/A	N/A	N/A	N/A
	CPLT	N/A	+	N/A	N/A	+	+	N/A	N/A	N/A	N/A
	ANIS	N/A	N/A	N/A	N/A	N/A	N/A	N/A	N/A	N/A	N/A
	ATYP	N/A	N/A	N/A	N/A	N/A	N/A	N/A	N/A	N/A	N/A
	COMMENT	N/A	N/A	N/A	N/A	HEM	HEM	HEM	HEM	N/A	N/A
	PT (sec)	11.1	10.7	10.4	10.0	10.9	11.4	11.4	10.5	10.7	10.4
	APTT (sec)	21.5	21.9	20.7	20.3	21.1	21.9	21.7	20.6	20.9	20.5
	FIB (mg/dL)	141	142	189	208	150	129	131	141	157	147
	DIME (ng/mL)	482.6	395.5	334.5	631.8	401.6	439.7	251.1	416.4	347.1	403.1
	FDP (FEU)	2.5	<2.5	<2.5	2.5	<2.5	<2.5	<2.5	<2.5	<2.5	<2.5

	Group (Necropsy Day)	Group 8 (Necropsy Day 180±5)									
	Animal ID (Sex)	18-158 (Female)									
	Treatment (Dose)	GTP-206 (4.5 x 10^{13} GC/Animal – High Dose)									
	Study Day	BL	0	7±1	14±2	28±3	60±3	90±4	120±4	150±4	180±5
Serum Chemistry	BUN (mg/dL)	QNS	21	22	20	19	22	18	18	13	13
	CREA (mg/dL)	0.6	0.4	0.4	0.5	0.4	0.4	0.4	0.5	0.4	0.4
	GLU (mg/dL)	QNS	73	79	75	122	61	102	94	99	122
	NA (mmol/L)	157	147	148	147	147	146	146	146	146	148
	K (mmol/L)	3.3	3.2	3.1	3.2	3.2	3.2	3.2	3.1	3.2	3.3
	CL (mmol/L)	113	108	108	106	109	105	108	107	107	111
	ALP (U/L)	693	587	526	702	677	689	599	714	808	873
	ALT (U/L)	35	27	27	31	31	34	29	31	36	35
	AST (U/L)	43	36	34	44	42	47	35	38	36	86
	TBIL (mg/dL)	0.2	0.2	0.2	0.2	0.2	0.2	0.2	0.2	0.2	0.2
	LDH (U/L)	QNS	265	254	383	299	342	204	272	252	605
	CPK (U/L)	222	255	188	211	171	345	407	193	247	6087
	GGT (U/L)	QNS	74	79	79	81	85	80	83	94	100
	TPRO (g/dL)	QNS	6.1	6.5	6.4	6.1	6.1	6.1	5.9	6.0	6.5
	ALB (g/dL)	4.4	4.2	4.3	4.1	4.1	4.0	4.3	4.1	4.3	4.3
	GLOB (g/dL)	QNS	1.9	2.2	2.3	2.0	2.1	1.8	1.8	1.7	2.2
	A/G	QNS	2.21	1.95	1.78	2.05	1.90	2.39	2.28	2.53	1.95
	CA (mg/dL)	10.4	9.7	9.9	9.9	9.3	9.7	9.7	9.4	10.0	10.0
	PHOS (mg/dL)	QNS	4.9	3.8	4.6	5.1	5.0	4.3	5.4	4.6	5.4
	ALD (U/L)	QNS	18.3	15.1	23.0	22.8	24.4	15.7	19.1	17.0	84.5
	CKMM (U/L)	QNS	183	126	153	100	238	350	147	179	5661
	CKM% (%)	QNS	71.8	66.8	72.4	58.4	68.9	86.1	76.0	72.5	93.0
	CKMB (U/L)	QNS	34	36	33	40	52	26	21	37	243
	CMB% (%)	QNS	13.4	19.2	15.6	23.2	15.0	6.5	10.8	15.0	4.0
	CKBB (U/L)	QNS	38	26	25	31	56	30	25	31	183
	CKB% (%)	QNS	14.8	14.0	12.0	18.3	16.1	7.4	13.1	12.5	3.0
	COMMENT	N/A	N/A	N/A	N/A	HEM	HEM	N/A	N/A	N/A	N/A

	Group (Necropsy Day)	Group 8 (Necropsy Day 180±5)									
	Animal ID (Sex)	18-170 (Male)									
	Treatment (Dose)	GTP-206 (4.5 x 10^{13} GC/Animal – High Dose)									
	Study Day	BL	0	7±1	14±2	28±3	60±3	90±4	120±4	150±4	180±5
Complete Blood Counts (Hematology) & Plasma Coagulation	WBC (10^3/µL)	17.44	6.86	7.99	12.56	19.33	24.34	8.77	10.33	9.63	7.42
	RBC (10^6/µL)	5.73	5.35	5.64	5.28	5.57	5.79	5.76	5.46	5.55	5.63
	HB (g/dL)	13.5	13.1	13.1	12.4	13.2	13.6	13.6	12.7	13.0	13.3
	HCT (%)	44.6	41.1	43.9	41.0	43.4	44.1	44.1	42.1	42.9	43.9
	MCV (fL)	77.9	76.8	77.8	77.6	78.0	76.2	76.6	77.1	77.2	78.0
	MCH (pg)	23.5	24.5	23.2	23.4	23.8	23.6	23.6	23.3	23.4	23.7
	MCHC (g/dL)	30.2	32.0	29.9	30.1	30.5	30.9	30.8	30.2	30.4	30.3
	PLT (10^3/µL)	312	352	305	307	488	383	376	467	414	475
	NEU% (%)	79.4	50.1	57.0	71.3	81.7	84.7	62.5	25.0	66.4	43.0
	NEU (10^3/µL)	13.85	3.44	4.55	8.95	15.79	20.62	5.48	2.58	6.40	3.19
	LYMPH% (%)	16.9	41.7	36.5	23.8	14.3	11.2	32.1	65.0	27.3	48.5
	LYMPH (10^3/µL)	2.95	2.86	2.92	2.99	2.76	2.72	2.82	6.72	2.63	3.59
	MONO% (%)	1.8	4.9	3.6	2.5	2.4	2.3	2.5	2.4	2.7	2.8
	MONO (10^3/µL)	0.32	0.34	0.29	0.32	0.46	0.56	0.22	0.25	0.26	0.21
	EOS% (%)	1.0	1.2	1.3	1.1	0.7	0.5	1.4	5.5	2.5	3.8
	EOS (10^3/µL)	0.17	0.08	0.10	0.14	0.13	0.12	0.12	0.57	0.24	0.28
	BAS% (%)	0.4	0.9	0.7	0.5	0.5	0.9	0.5	0.6	0.3	0.4
	BAS (10^3/µL)	0.07	0.06	0.06	0.06	0.10	0.21	0.05	0.07	0.03	0.03
	LUC% (%)	0.4	1.1	0.9	0.8	0.5	0.4	1.0	1.5	0.8	1.5
	LUC (10^3/µL)	0.07	0.08	0.07	0.10	0.09	0.11	0.09	0.15	0.08	0.11
	MAC	+	+	+	+	+	N/A	+	+	+	+
	LPLT	N/A	N/A	+	N/A	N/A	N/A	N/A	N/A	N/A	N/A
	CPLT	+	+	+	+	+	N/A	+	N/A	+	N/A
	ANIS	N/A	N/A	N/A	N/A	N/A	N/A	N/A	N/A	N/A	N/A
	ATYP	N/A	N/A	N/A	N/A	N/A	N/A	N/A	N/A	N/A	N/A
	COMMENT	N/A	N/A	N/A	N/A	N/A	HEM	N/A	N/A	HEM	N/A
	PT (sec)	10.6	11.2	10.7	10.4	10.8	12.0	12.1	11.4	11.6	11.1
	APTT (sec)	22.4	22.9	23.4	23.9	23.1	24.7	25.1	24.1	23.6	24.4
	FIB (mg/dL)	179	167	195	201	191	107	155	183	176	166
	DIME (ng/mL)	351.0	242.0	232.9	351.0	372.1	6881.	364.6	347.9	419.1	209.9
	FDP (FEU)	<2.5	<2.5	<2.5	2.5	2.5	20.0	<2.5	<2.5	<2.5	<2.5

	Group (Necropsy Day)	Group 8 (Necropsy Day 180±5)									
	Animal ID (Sex)	18-170 (Male)									
	Treatment (Dose)	GTP-206 (4.5 x 10^{13} GC/Animal – High Dose)									
	Study Day	BL	0	7±1	14±2	28±3	60±3	90±4	120±4	150±4	180±5
Serum Chemistry	BUN (mg/dL)	19	18	22	15	24	24	18	14	19	17
	CREA (mg/dL)	0.6	0.6	0.6	0.6	0.5	0.4	0.4	0.4	0.5	0.4
	GLU (mg/dL)	70	69	65	73	48	46	68	76	75	71
	NA (mmol/L)	150	150	151	149	148	143	150	150	147	146
	K (mmol/L)	3.1	3.3	3.3	3.0	3.4	3.4	3.4	3.7	4.0	3.6
	CL (mmol/L)	109	109	111	110	109	107	111	112	109	109
	ALP (U/L)	883	881	679	771	790	1044	1076	901	905	743
	ALT (U/L)	27	25	20	26	33	33	26	26	36	28
	AST (U/L)	48	31	33	38	40	40	30	32	44	32
	TBIL (mg/dL)	0.2	0.2	0.2	0.2	0.2	0.3	0.2	0.2	0.2	0.2
	LDH (U/L)	384	205	202	252	269	239	221	247	381	243
	CPK (U/L)	1097	125	126	181	418	386	218	265	980	266
	GGT (U/L)	76	69	67	68	84	87	88	90	93	81
	TPRO (g/dL)	6.5	6.2	6.5	6.1	6.5	6.3	6.2	5.9	6.3	6.4
	ALB (g/dL)	4.0	4.0	4.1	3.9	4.1	4.1	3.9	3.8	3.9	3.9
	GLOB (g/dL)	2.5	2.2	2.4	2.2	2.4	2.2	2.3	2.1	2.4	2.5
	A/G	1.60	1.82	1.71	1.77	1.71	1.86	1.70	1.81	1.63	1.56
	CA (mg/dL)	9.6	9.5	9.8	9.4	9.5	9.3	9.3	9.6	9.7	9.7
	PHOS (mg/dL)	4.6	4.9	5.6	5.0	5.6	6.2	5.0	5.2	6.3	6.7
	ALD (U/L)	34.7	18.7	14.0	23.8	22.2	18.2	17.1	19.6	31.5	20.9
	CKMM (U/L)	963	58	95	114	298	268	128	164	859	164
	CKM% (%)	87.8	46.2	75.1	62.9	71.3	69.5	58.7	61.7	87.7	61.8
	CKMB (U/L)	77	33	18	28	52	42	36	41	47	31
	CMB% (%)	7.0	26.0	14.4	15.4	12.4	10.8	16.6	15.4	4.8	11.7
	CKBB (U/L)	57	35	13	39	68	76	54	60	73	70
	CKB% (%)	5.2	27.7	10.4	21.7	16.2	19.7	24.7	22.8	7.4	26.5
	COMMENT	N/A	N/A	N/A	N/A	N/A	N/A	N/A	N/A	HEM	N/A

CEREBROSPINAL FLUID

Treatment	Dose (GC/Animal)	Group	Necropsy Day	Animal ID	Sex	Study Day	WBC Count (per µL)	RBC Count (per µL)	Total Protein (mg/dL)	Glucose (mg/dL)	Color	Clarity
ITFFB	N/A	1	90±4	18-162	F	0	0	0	6	65	colorless	clear
						7±1	1	1300	8	40	colorless	slightly cloudy
						14±2	0	0	7	58	colorless	clear
						28±3	0	80	5	41	colorless	clear
						60±3	1	3	6	34	colorless	clear
						90±4	1	450	11	59	colorless	clear
		5	180±5	18-159	M	0	1	0	9	59	colorless	clear
						7±1	0	10	10	53	colorless	clear
						14±2	2	1	10	43	colorless	clear
						28±3	0	600	12	59	colorless	clear
						60±3	1	10	12	39	colorless	clear
						90±4	4	1250	19	44	colorless	slightly cloudy
						120±4	1	0	13	46	colorless	clear
						150±4	2	3200	19	59	colorless	slightly cloudy
						180±5	1	10	13	53	colorless	clear
GTP-206	4.5×10^{12} (Low Dose)	2	90±4	18-091	M	0	1	10	8	57	colorless	clear
						7±1	1	1	11	59	colorless	clear
						14±2	0	10	11	53	colorless	clear
						28±3	1	0	9	52	colorless	clear
						60±3	1	20	8	54	colorless	clear
						90±4	1	1	10	49	colorless	clear
				18-168	F	0	2	0	11	49	colorless	clear
						7±1	0	1	12	39	colorless	clear
						14±2[a]	8	10	12	40	colorless	clear
						28±3[b]	1	0	15	39	colorless	clear
						60±3	1	1	14	37	colorless	clear
						90±4	4	50	14	50	colorless	clear

Treatment	Dose (GC/Animal)	Group	Necropsy Day	Animal ID	Sex	Study Day	WBC Count (per µL)	RBC Count (per µL)	Total Protein (mg/dL)	Glucose (mg/dL)	Color	Clarity
GTP-206	4.5 x 10^{12} (Low Dose)	2	90±4	18-173	F	0	1	0	7	64	colorless	clear
						7±1	0	0	8	58	colorless	clear
						14±2	1	10	7	54	colorless	clear
						28±3	0	0	8	60	colorless	clear
						60±3	1	10	7	57	colorless	clear
						90±4	1	150	9	55	colorless	clear
		6	180±5	18-042	F	0	0	0	9	60	colorless	clear
						7±1	2	0	10	44	colorless	clear
						14±2	0	20	9	45	colorless	clear
						28±3	3	30	12	52	colorless	clear
						60±3	1	20	11	51	colorless	clear
						90±4	0	10	10	45	colorless	clear
						120±4	2	1	11	50	colorless	clear
						150±4	0	0	11	47	colorless	clear
						180±5	0	0	9	54	colorless	clear
			180±5	18-121	F	0	1	10	8	58	colorless	clear
						7±1	8	7500	62	60	colorless	cloudy
						14±2	0	0	7	51	colorless	clear
						28±3	1	450	10	59	colorless	clear
						60±3	2	1	11	59	colorless	clear
						90±4	0	0	11	54	colorless	clear
						120±4	1	0	15	54	colorless	clear
						150±4	1	3	13	56	colorless	clear
						180±5	3	350	12	53	colorless	clear

Treatment	Dose (GC/Animal)	Group	Necropsy Day	Animal ID	Sex	Study Day	WBC Count (per µL)	RBC Count (per µL)	Total Protein (mg/dL)	Glucose (mg/dL)	Color	Clarity
GTP-206	4.5×10^{12} (Low Dose)	6	180±5	18-171	M	0	0	0	4	67	colorless	clear
						7±1	0	0	6	44	colorless	clear
						14±2	0	0	5	63	colorless	clear
						28±3	0	0	5	47	colorless	clear
						60±3	0	1	5	44	colorless	clear
						90 ±4	0	0	6	53	colorless	clear
						120±4	0	0	6	55	colorless	clear
						150±4	0	1	7	42	colorless	clear
						180±5	0	10	5	44	colorless	clear
	1.5×10^{13} (Mid-Dose)	3	90±4	18-167	M	0	0	40	8	57	colorless	clear
						7±1	0	0	8	49	colorless	clear
						14±2	0	1	9	49	colorless	clear
						28±3	1	0	8	41	colorless	clear
						60±3	1	1	10	47	colorless	clear
						90±4	0	0	8	55	colorless	clear
				18-176	M	0	0	0	7	56	colorless	clear
						7±1	0	0	7	57	colorless	clear
						14±2	0	10	7	58	colorless	clear
						28±3	1	0	9	40	colorless	clear
						60±3	0	0	8	46	colorless	clear
						90±4	26	2500	16	51	colorless	clear
				18-187	F	0	0	1	5	65	colorless	clear
						7±1	0	20	5	79	colorless	clear
						14±2	0	1190	10	75	colorless	clear
						28±3	1	1080	13	69	colorless	clear
						60±3	1	10	8	57	colorless	clear
						90±4	0	0	7	55	colorless	clear

Treatment	Dose (GC/Animal)	Group	Necropsy Day	Animal ID	Sex	Study Day	WBC Count (per µL)	RBC Count (per µL)	Total Protein (mg/dL)	Glucose (mg/dL)	Color	Clarity
GTP-206	1.5 x 10^{13} (Mid-Dose)	7	180±5	18-055	M	0	0	1	7	50	colorless	clear
						7±1	0	1	6	55	colorless	clear
						14±2	0	0	5	53	colorless	clear
						28±3	0	90	9	48	colorless	clear
						60±3	4	1750	10	44	colorless	clear
						90 ±4	0	1	7	52	colorless	clear
						120±4	0	0	20	59	colorless	clear
						150±4	1	0	80	52	colorless	clear
						180±5	0	0	16	48	colorless	clear
				18-181	F	0	2	550	7	56	colorless	clear
						7±1	0	0	4	64	colorless	clear
						14±2	1	10	7	58	colorless	clear
						28±3	4	10	8	55	colorless	clear
						60±3	1	20	9	33	colorless	clear
						90 ±4	0	20	5	34	colorless	clear
						120±4	0	10	7	70	colorless	clear
						150±4	1	0	9	61	colorless	clear
						180±5	18	12500	86	80	light red	cloudy
				18-183	F	0	4	10	17	58	colorless	cloudy
						7±1	1	0	9	62	colorless	clear
						14±2	0	0	8	64	colorless	clear
						28±3	1	10	7	50	colorless	clear
						60±3	1	10	9	45	colorless	clear
						90 ±4	0	30	8	60	colorless	clear
						120±4	2	10	10	53	colorless	clear
						150±4	1	100	9	57	colorless	clear
						180±5	63	22500	172	67	light red	cloudy
	4.5 x 10^{13} (High Dose)	4	90±4	18-080	M	0	0	0	6	49	colorless	clear
						7±1	0	10	9	57	colorless	clear
						14±2	0	0	7	58	colorless	clear
						28±3	1	0	10	50	colorless	clear
						60±3	0	1	6	50	colorless	clear
						90 ±4	0	1	7	50	colorless	clear

Treatment	Dose (GC/Animal)	Group	Necropsy Day	Animal ID	Sex	Study Day	WBC Count (per µL)	RBC Count (per µL)	Total Protein (mg/dL)	Glucose (mg/dL)	Color	Clarity
GTP-206	4.5×10^{13} (High Dose)	4	90±4	18-166	M	0	1	160	7	73	colorless	clear
						7±1	0	0	5	54	colorless	clear
						14±2	0	0	6	48	colorless	clear
						28±3	1	0	7	57	colorless	clear
						60±3	0	0	7	53	colorless	clear
						90 ±4	0	0	9	57	colorless	clear
				18-185	F	0	1	1	7	47	colorless	clear
						7±1	2	0	6	50	colorless	clear
						14±2	1	20	7	54	colorless	clear
						28±3	5	10	8	45	colorless	clear
						60±3	2	1	9	47	colorless	clear
						90 ±4	0	40	8	51	colorless	clear
		8	180±5	18-038	F	0	1	10	5	47	colorless	clear
						7±1	1	0	6	55	colorless	clear
						14±2	22	30	8	57	colorless	clear
						28±3	0	0	11	73	colorless	clear
						60±3	4	10	26	61	colorless	clear
						90 ±4	0	20	21	58	colorless	clear
						120±4	1	10	9	61	colorless	clear
						150±4	1	0	11	52	colorless	clear
						180±5	1	10	10	59	colorless	clear
				18-158	F	0	1	0	9	43	colorless	clear
						7±1	1	0	10	50	colorless	clear
						14±2	20	50	13	56	colorless	clear
						28±3	7	10	13	60	colorless	clear
						60±3	9	10	18	45	colorless	clear
						90 ±4	0	10	11	57	colorless	clear
						120±4	1	3	10	53	colorless	clear
						150±4	1	0	12	52	colorless	clear
						180±5	0	1	12	67	colorless	clear

Treatment	Dose (GC/Animal)	Group	Necropsy Day	Animal ID	Sex	Study Day	WBC Count (per µL)	RBC Count (per µL)	Total Protein (mg/dL)	Glucose (mg/dL)	Color	Clarity
GTP-206	4.5×10^{13} (High Dose)	8	180±5	18-170	M	0	0	2	8	59	colorless	clear
						7±1	0	1	9	49	colorless	clear
						14±2	0	0	9	47	colorless	clear
						28±3	1	120	13	34	colorless	clear
						60±3	1	10	9	35	colorless	clear
						90 ±4	1	10	11	47	colorless	clear
						120±4	0	0	9	55	colorless	clear
						150±4	8	11750	31	61	colorless	slightly cloudy
						180±5	1	290	12	55	colorless	clear

[a]Animal 18-168 exhibited neutrophilic pleocytosis on Day 14±2.
[b]Animal 18-168 exhibited excessive cytolysis on Day 28±3.
Abbreviations: CSF, cerebrospinal fluid; F, female; GC, genome copies; ID, identification number; ITFFB, intrathecal final formulation buffer; M, male; N/A, not applicable; RBC, red blood cell; WBC, white blood cell.
