# Supplementary material for: Efficacy and Safety of a Krabbe Disease Gene Therapy
Source: Hum Gene Ther. 2022 May 16;33(9-10):499–517. doi: 10.1089/hum.2021.245 (PMC9142772; doi:10.1089/hum.2021.245)
Supplement: Supplemental data [file Suppl_TableS1.docx]

Table S1. Neurobehavioral scoring, Twitcher mice.

| **Assessment Category** | **Observation** | **Clinical**  **Score** |
| --- | --- | --- |
| Hind Limb Clasping | No clasping | 0 |
|  | Non-permanent clasping | 1 |
|  | Permanent clasping | 2 |
| Gait | Normal | 0 |
|  | Slightly abnormal gait but mouse moves easily and spontaneously | 1 |
|  | Markedly abnormal gait, reduced spontaneous mobility | 2 |
|  | Severe difficulty moving forward, dragging of hind legs | 3 |
| Tremors | Normal | 0 |
|  | Minimal tremors, only visible when mice are immobile | 1 |
|  | Moderate tremors, noticeable at rest and while moving. Twitching | 2 |
|  | Marked tremors and twitching, obvious both at rest and while moving. | 3 |
| Spinal Curvature | Normal | 0 |
|  | Mild kyphosis (curved spine), but able to straighten spine completely | 1 |
|  | Unable to straighten spine completely; maintenance of persistent mild kyphosis | 2 |
|  | Maintenance of pronounced kyphosis while walking or sitting | 3 |
| Fur Quality | Normal | 0 |
|  | Any abnormality (scruffed, alopecia, etc.) | 1 |
