# Supplementary material for: Efficacy and Safety of a Krabbe Disease Gene Therapy
Source: Hum Gene Ther. 2022 May 16;33(9-10):499–517. doi: 10.1089/hum.2021.245 (PMC9142772; doi:10.1089/hum.2021.245)
Supplement: Supplemental data [file Suppl_TableS2.docx]

**Table S2.** Neurological scoring, Krabbe dog study.

| Dog # | Genotype | Treatment | Age (weeks) | Proprioception deficit (0-2) | Ataxia (0-3) | Head tremors, truncal sway (0-3) | Spinal reflexes (0-2) | Muscle atrophy (0-3) | Lack of Menace response (0-1) |
| --- | --- | --- | --- | --- | --- | --- | --- | --- | --- |
| K948 | Krabbe | Vehicle | 11 | 2 | 3 | 1 | 1 | 1 | 1 |
| K928 | WT | Vehicle | 13 | 0 | 1 | 0 | 0 | 0 | 0 |
|  |  |  | 24 | 0 | 1 | 0 | 0 | 0 | 0 |
|  |  |  | 38 | 0 | 0 | 0 | 0 | 0 | 0 |
|  |  |  | 52 | 0 | 0 | 0 | 0 | 0 | 0 |
|  |  |  | 81 | 0 | 0 | 0 | 0 | 0 | 0 |
| K933 | Krabbe | AAV | 13 | 0 | 0 | 0 | 0 | 0 | 0 |
|  |  |  | 24 | 0 | 1 | 0 | 0 | 0 | 0 |
|  |  |  | 38 | 0 | 0 | 0 | 0 | 0 | 0 |
|  |  |  | 52 | 0 | 1 | 0 | 0 | 0 | 0 |
|  |  |  | 81 | 0 | 1 | 0 | 0 | 0 | 0 |
| K937 | Krabbe | AAV | 12 | 0 | 0 | 0 | 0 | 0 | 0 |
|  |  |  | 24 | 0 | 1 | 0 | 0 | 0 | 0 |
| K938 | Krabbe | AAV | 12 | 0 | 0 | 0 | 0 | 0 | 0 |
|  |  |  | 24 | 0 | 1 | 0 | 0 | 0 | 0 |
| K939 | Krabbe | AAV | 12 | 0 | 0 | 0 | 0 | 0 | 0 |
|  |  |  | 24 | 0 | 1 | 0 | 0 | 0 | 0 |
