# Supplementary material for: Efficacy and Safety of a Krabbe Disease Gene Therapy
Source: Hum Gene Ther. 2022 May 16;33(9-10):499–517. doi: 10.1089/hum.2021.245 (PMC9142772; doi:10.1089/hum.2021.245)
Supplement: Supplemental data [file Supp_FigureS1.docx]

**
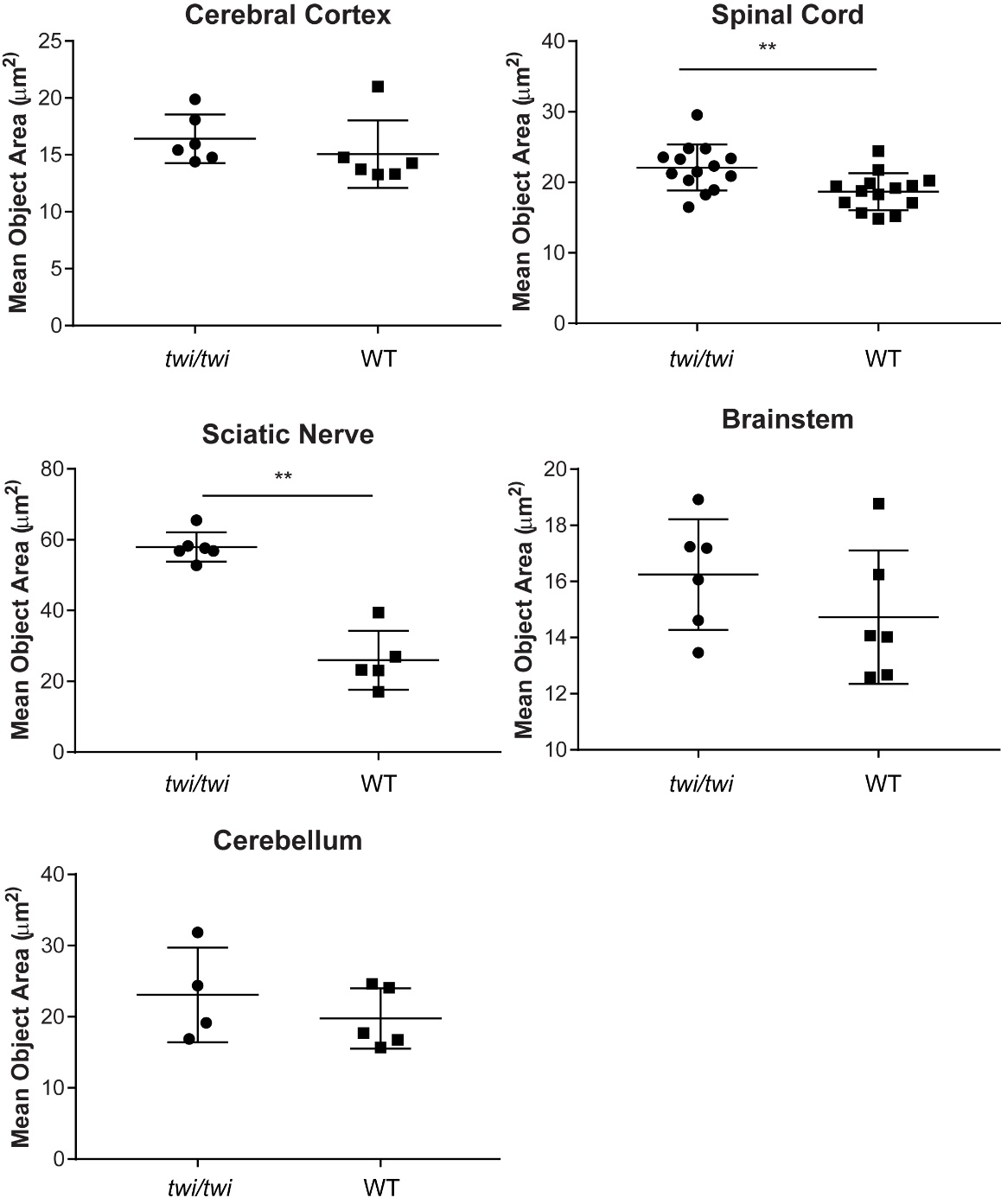
**

**Figure S1. Baseline neuroinflammation in PND12 juvenile Twitcher mice**

Quantification of neuroinflammation measured by mean object area of IBA1-positive cells on sections of brain (cortex, cerebellum, brainstem), spinal cord, and sciatic nerve stained by immunohistochemistry against IBA1 (macrophage/microglial marker). Untreated Twitcher mice at PND 12-14 (baseline age at injection for the MED study). ** *p* < 0.01 Mann-Whitney rank test, alpha = 0.05.
