# Supplementary material for: Efficacy and Safety of a Krabbe Disease Gene Therapy
Source: Hum Gene Ther. 2022 May 16;33(9-10):499–517. doi: 10.1089/hum.2021.245 (PMC9142772; doi:10.1089/hum.2021.245)
Supplement: Supplemental data [file Supp_FigureS2.docx]

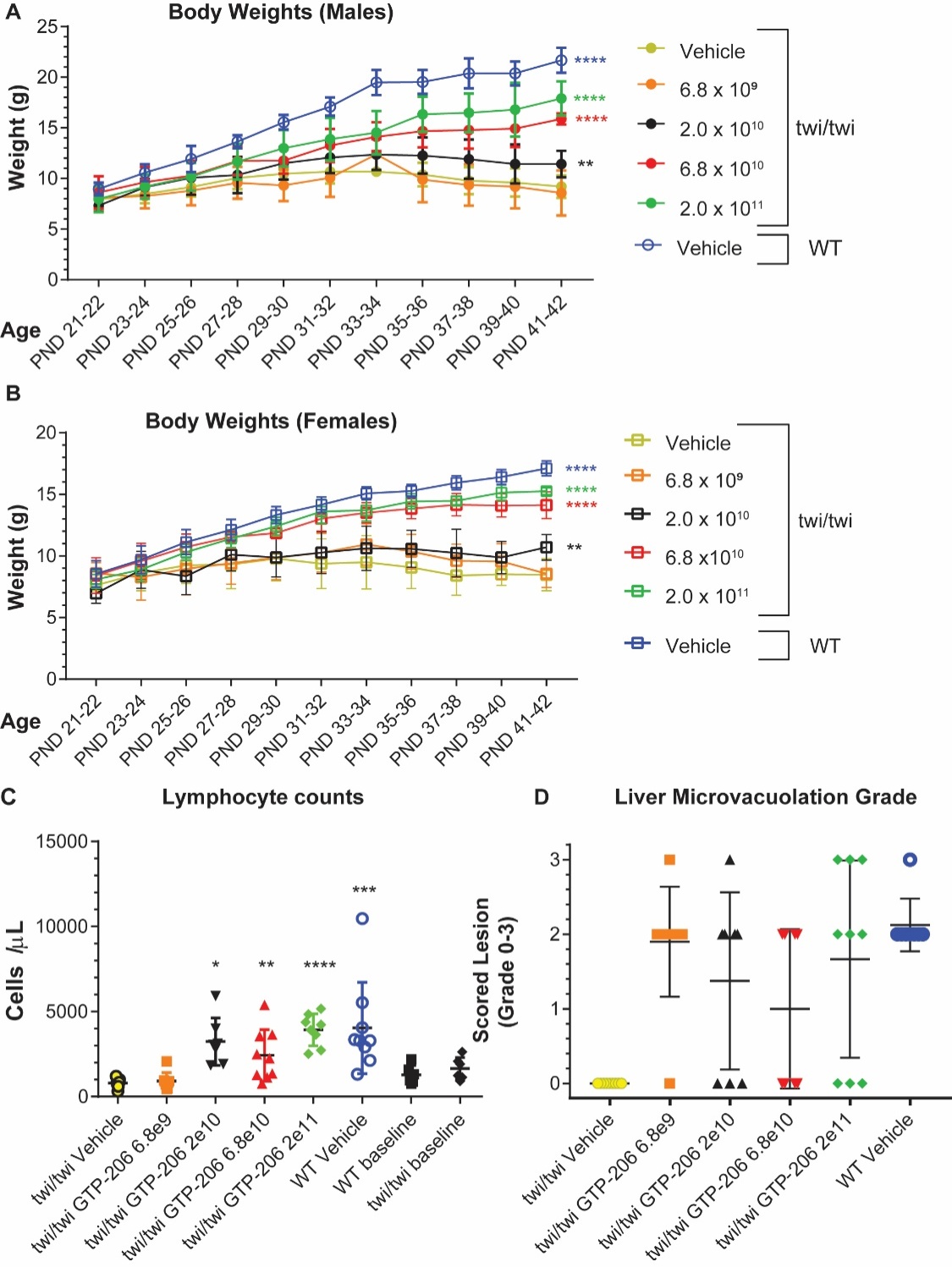


**Figure S2. Somatic pathology correction in twitcher mice**

Twitcher mice were treated on PND 12-14 ICV with either 4 µl of artificial CSF (vehicle, n = 17), or 4 µl of AAVhu68.CB7.hGALCco.rBG at the following doses: 6.8 x 10^9^ GC (n = 16), 2 x 10^10^ GC (n = 17), 6.8 x 10^10^ (n = 17), or 2 x 10^11^ GC (n = 16). WT littermates were treated ICV with 4 µl of artificial CSF (n = 17). The operator was blinded to the mice genotype and treatment. A-B Body weight gain in males (A) and females (B) **** p < 0.000, ** p < 0.01 linear mixed-effect modeling change over time compared to the vehicle Twitcher group, alpha = 0.05. C. Whole blood lymphocyte counts at necropsy timepoint * p < 0.05 ** p < 0.01, *** p < 0.001, **** p < 0.0001, Kruskal-Wallis test followed by post-hoc Dunn’s multiple comparison test, alpha = 0.05, comparison to vehicle Twitcher mice. D. Liver hepatocellular microvacuolation grade on histopathology (0 = absence, 1 = minimal, 2 = mild, 3 = moderate).
