# Supplementary material for: Efficacy and Safety of a Krabbe Disease Gene Therapy
Source: Hum Gene Ther. 2022 May 16;33(9-10):499–517. doi: 10.1089/hum.2021.245 (PMC9142772; doi:10.1089/hum.2021.245)
Supplement: Supplemental data [file VideoS1-S3.zip › Video_Legends.docx]

**Supplemental Video File Legends**

**Supplemental Data Video 1** – Sham-treated Krabbe Dog at 12 weeks

The animal was recorded one week before reaching humane endpoint. He presents severe ataxia and hindlimb weakness that cause frequent falls and limits ability to ambulate. Head tremors are noticeable. Despite the marked neurological and motor signs, the dog is in good spirits and engages in play with caretakers when offered toys.

**Supplemental Data Video 2** – AAV-treated Krabbe Dog at 38 weeks

The animal was recorded one week prior to scheduled euthanasia per study protocol. He presents normal ambulation with occasional slipping on the smooth floor surface. The dog is playful and can walk, run, and fetch toys.

**Supplemental Data Video 3** – AAV-treated Krabbe Dog at 82 weeks

The animal was recorded one week before reaching humane endpoint due to feeding difficulties and marked weight loss. He presents normal ambulation and gait. The dog is playful and can walk, run, and stand on his hindlimbs.
