# Supplementary material for: Efficacy and Safety of a Krabbe Disease Gene Therapy
Source: Hum Gene Ther. 2022 May 16;33(9-10):499–517. doi: 10.1089/hum.2021.245 (PMC9142772; doi:10.1089/hum.2021.245)
Supplement: Supplemental data [file Supp_FigureS3.docx]

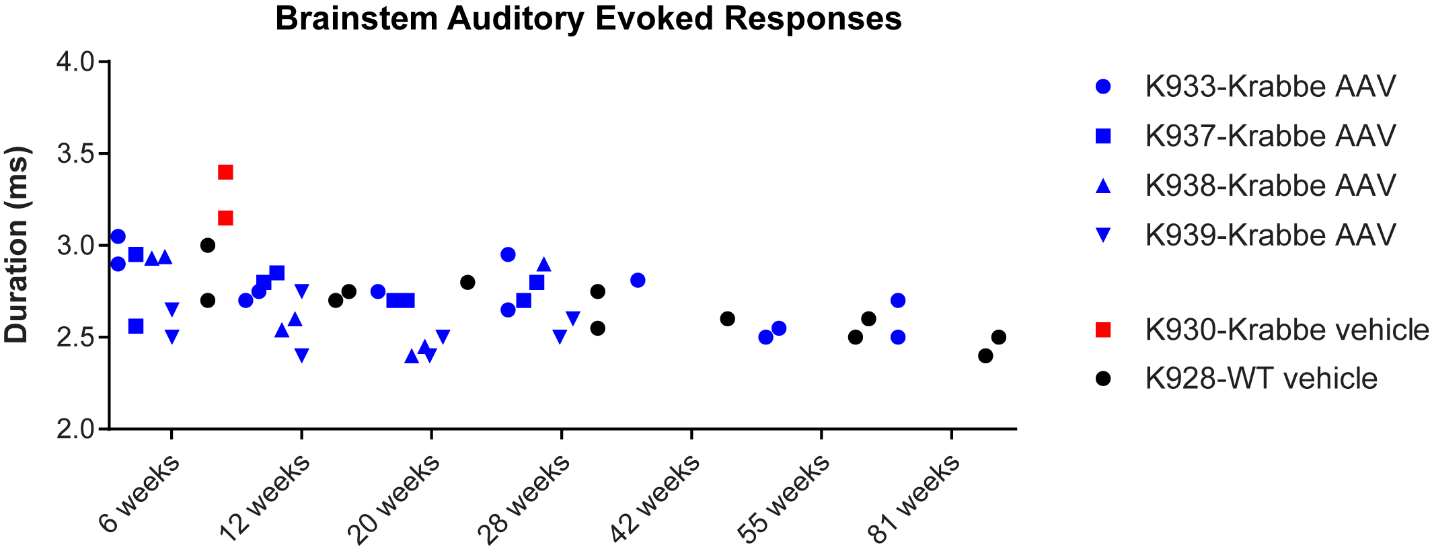


**Figure S3. Brainstem auditory evoked responses in Krabbe dogs**

Interpeak latency (IPL) between the first and the fifth wave representing the conduction velocity within the central auditory pathways of the medulla. Krabbe dogs treated ICM at 2-3 weeks of age with 1 ml of artificial CSF (vehicle, n=2) or with 3 x 10^13^ GC of AAVhu68.CB7.cGALCco.rBG in 1 ml (n = 4). A WT littermate that received 1 ml of artificial CSF ICM is used as control. Two treated dogs were sacrificed at scheduled timepoint 6 months post injection for tissue collection while 2 treated dogs and the vehicle-treated Krabbe dogs were followed until humane endpoint. One Krabbe vehicle dog is missing (K948) because no response could be recorded after the highest stimulation.
