# Supplementary material for: Efficacy and Safety of a Krabbe Disease Gene Therapy
Source: Hum Gene Ther. 2022 May 16;33(9-10):499–517. doi: 10.1089/hum.2021.245 (PMC9142772; doi:10.1089/hum.2021.245)
Supplement: Supplemental data [file Supp_FigureS6.docx]

**
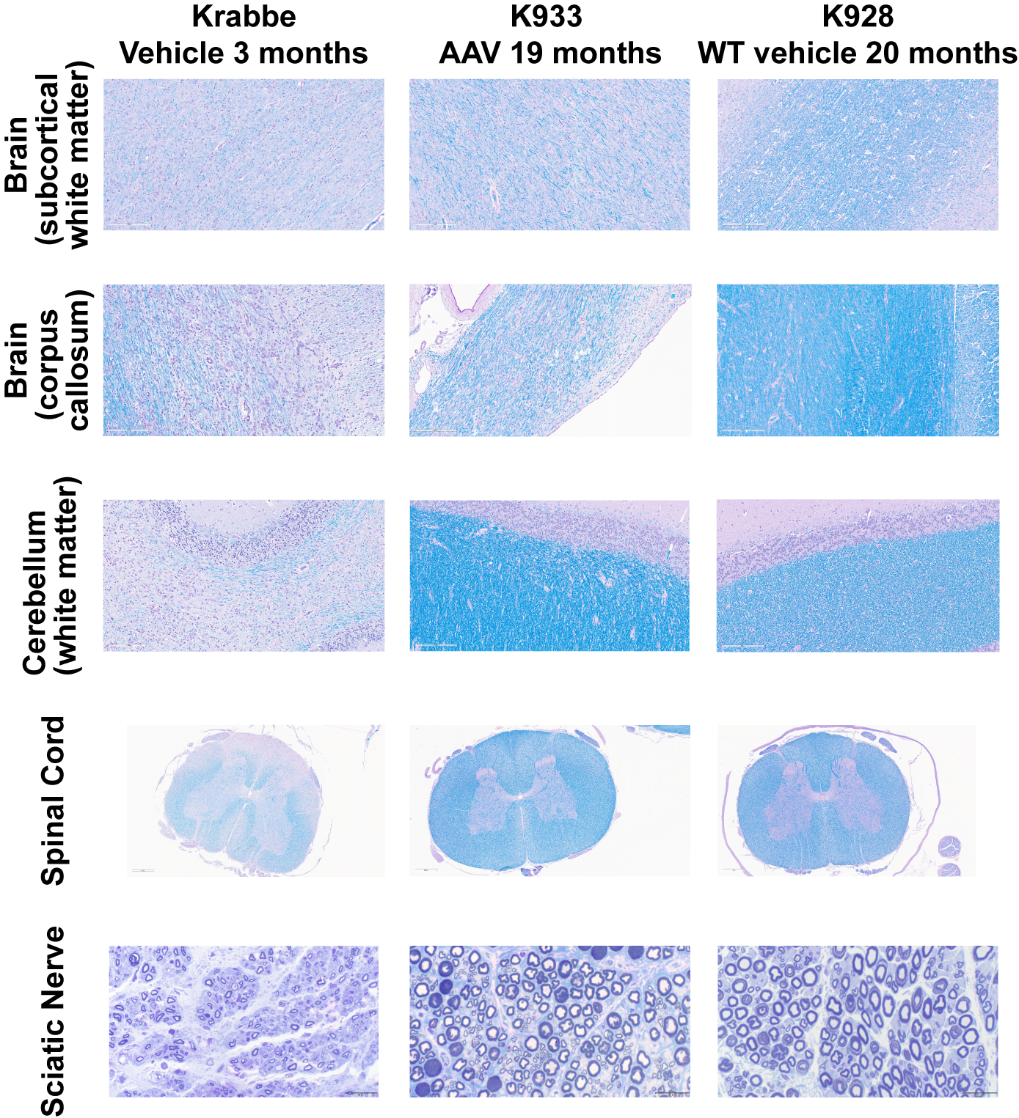
**

**Figure S6. ICM efficacy study in Krabbe dogs, myelination**

Representative pictures from luxol fast blue – PAS-stained sections obtained from the brain, cerebellum, and spinal cord; toluidine blue-stained semi-thin sections from sciatic nerve in Krabbe dogs that received an ICM administration of artificial CSF (vehicle, 3 months of age, humane endpoint) or AAV. A WT littermate that received artificial CSF and was euthanized at 20 months of age is shown as control. Treated dogs were euthanized at scheduled endpoint (K938, 6.5 months of age) or humane endpoint (K937 and K933 9 to 19.5 months of age). Myelin is stained in blue.
