# Supplementary material for: Efficacy and Safety of a Krabbe Disease Gene Therapy
Source: Hum Gene Ther. 2022 May 16;33(9-10):499–517. doi: 10.1089/hum.2021.245 (PMC9142772; doi:10.1089/hum.2021.245)
Supplement: Supplemental data [file Supp_FigureS4.docx]

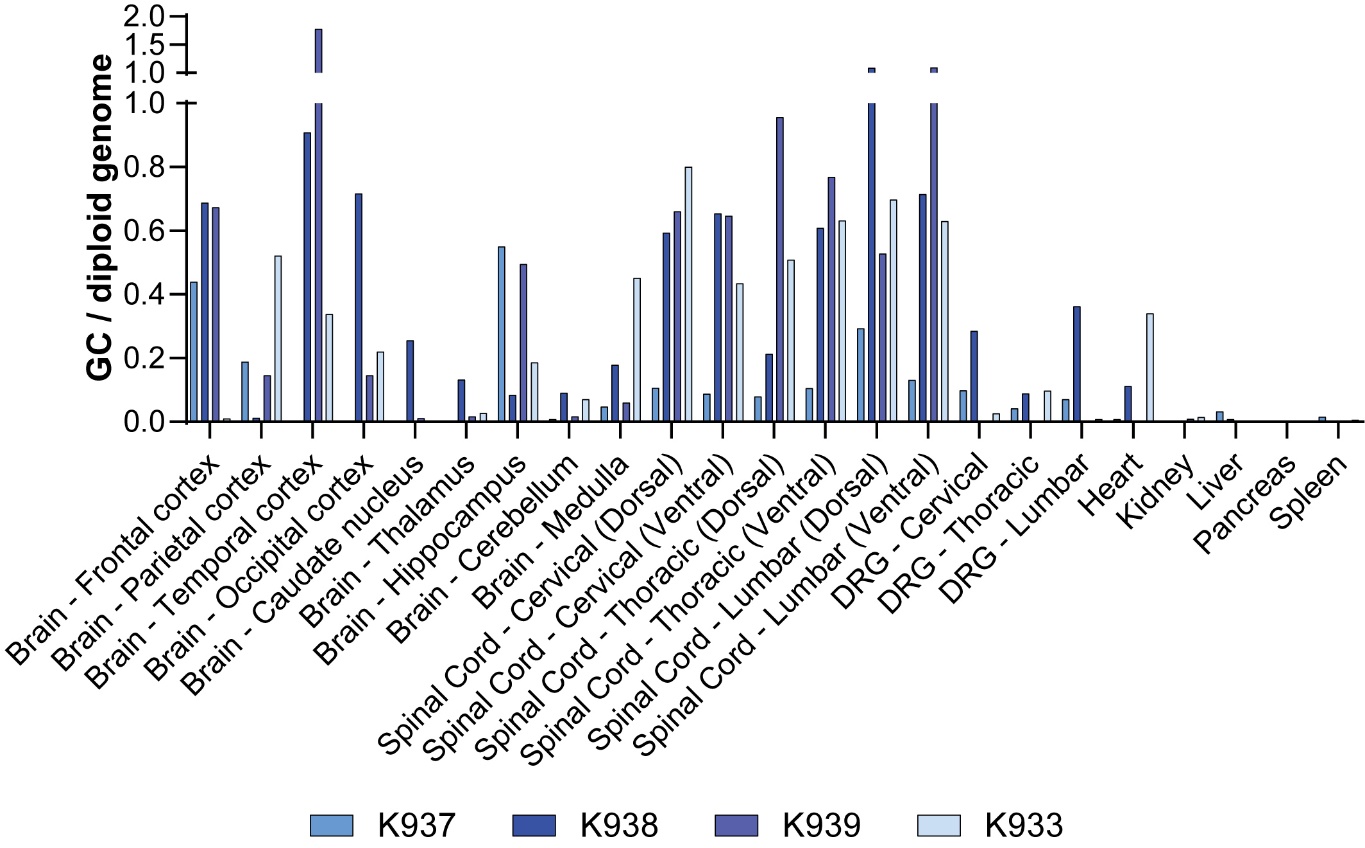


**Figure S4. Vector genome biodistribution in Krabbe dogs**

Vector genome copies were quantified by taqman qPCR with primers and probe targeting the rabit beta globin polyA. Results expressed in genomes copies (GC) per diploid genome.
