# Supplementary material for: Efficacy and Safety of a Krabbe Disease Gene Therapy
Source: Hum Gene Ther. 2022 May 16;33(9-10):499–517. doi: 10.1089/hum.2021.245 (PMC9142772; doi:10.1089/hum.2021.245)
Supplement: Supplemental data [file Supp_FigureS5.docx]

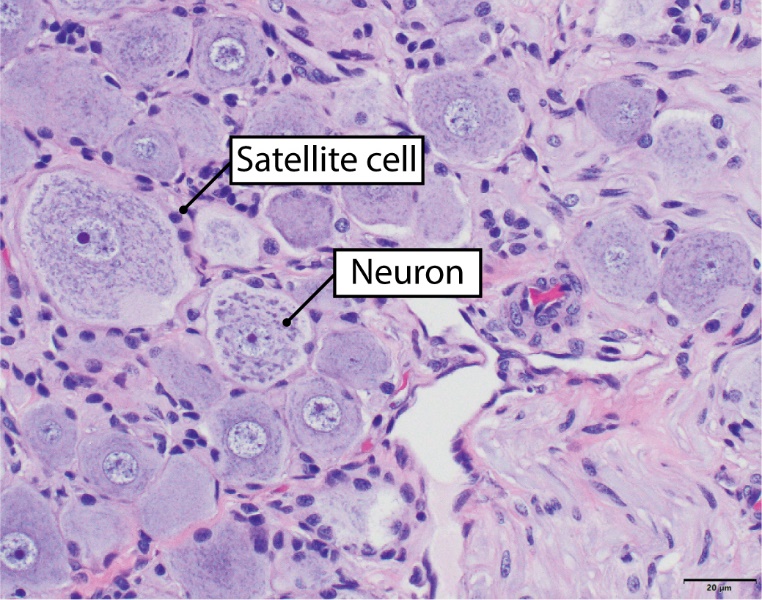


**Figure S5. Dorsal root ganglia histology in dogs treated with ICM AAV.cGALC**

Representative picture from hematoxylin-eosin stained section of a lumbar dorsal root ganglion from a Krabbe dog that received an ICM administration of 3 x 10^13^ GC AAVhu68.CB7.cGALC at 2 weeks of age and was euthanized 6 months post injection (K938). Normal histology, absence of findings.
