# Supplementary material for: Efficacy and Safety of a Krabbe Disease Gene Therapy
Source: Hum Gene Ther. 2022 May 16;33(9-10):499–517. doi: 10.1089/hum.2021.245 (PMC9142772; doi:10.1089/hum.2021.245)
Supplement: Supplemental data [file Supp_FigureS7.docx]

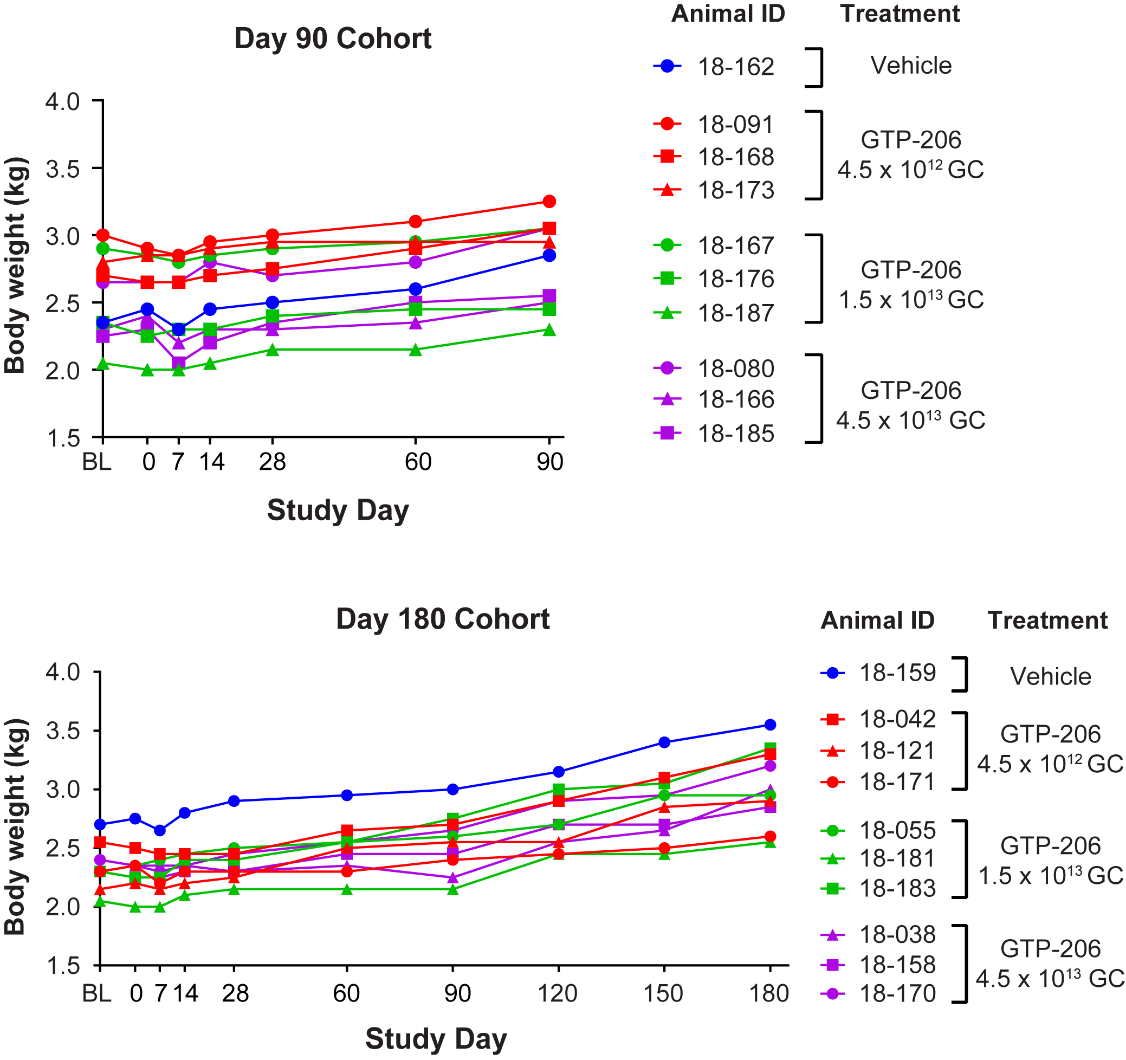


**Figure S7. Body weight gains, NHP toxicology study**

Body weight gain of juvenile rhesus macaques treated ICM with artificial CSF (vehicle, n=2) or AAVhu68.CB7.hGALCco.rBG at the following doses: 4.5 x 10^12^ GC (low dose, n=6), 1.5 x 10^13^ GC (mid dose, n-6), or 4.5 x 10^13^ (high dose, n = 6). Half of the animals were euthanized for tissue collection 3 months post dosing (Day 90 cohort); the other half were euthanized 6 months post dosing (Day 180 cohort).
