# Supplementary material for: Efficacy and Safety of a Krabbe Disease Gene Therapy
Source: Hum Gene Ther. 2022 May 16;33(9-10):499–517. doi: 10.1089/hum.2021.245 (PMC9142772; doi:10.1089/hum.2021.245)
Supplement: Supplemental data [file Suppl_TableS3.docx]

**Table S3.** Neutralizing antibody titers to AAVhu68, NHP toxicology study

| Group # | Test Article | Dose (GC) | ID | Necropsy Date | AAVhu68 NAb in HEK293 cells | | | | | | |
| --- | --- | --- | --- | --- | --- | --- | --- | --- | --- | --- | --- |
|  |  |  |  |  |  |  |  |  |  |  |  |
|  |  |  |  |  | BSL | Day 28 | Day 60 | Day 90 | Day 120 | Day 150 | Day 180 |
| 1 | ITFFB | N/A | 18-162 | Day 90 | 80 | 320 | 160 | 80 | N/A | | |
| 5 | ITFFB | N/A | 18-159 | Day 180 | < 5 | < 5 | < 5 | < 5 | < 5 | < 5 | < 5 |
| 2 | AAVhu68.  hGALCco | 4.5x10^12^ | 18-091 | Day 90 | < 5 | 320 | 320 | 640 | N/A | | |
|  |  |  | 18-168 |  | 10 | 640 | 1280 | 1280 |  |  |  |
|  |  |  | 18-173 |  | 80 | 2560 | 1280 | 1280 |  |  |  |
| 6 |  | 4.5x10^12^ | 18-042 | Day 180 | 80 | 320 | 640 | 160 | 320 | 1280 | 1280 |
|  |  |  | 18-121 |  | 5 | 320 | 640 | 1280 | 640 | 1280 | 1280 |
|  |  |  | 18-171 |  | 5 | 320 | 640 | 640 | 640 | 160 | 160 |
| 3 |  | 1.5x10^13^ | 18-167 | Day 90 | 160 | 2560 | 640 | 640 | N/A | | |
|  |  |  | 18-176 |  | < 5 | 640 | 320 | 640 |  |  |  |
|  |  |  | 18-187 |  | < 5 | 2560 | 5120 | 2560 |  |  |  |
| 7 |  | 1.5x10^13^ | 18-055 | Day 180 | < 5 | 80 | 320 | 320 | 160 | 160 | 160 |
|  |  |  | 18-181 |  | < 5 | 320 | 1280 | 320 | 320 | 320 | 640 |
|  |  |  | 18-183 |  | < 5 | 320 | 2560 | 1280 | 2560 | 1280 | 1280 |
| 4 |  | 4.5x10^13^ | 18-080 | Day 90 | 10 | 1280 | 2560 | 1280 | N/A | | |
|  |  |  | 18-166 |  | 80 | 10240 | 5120 | 1280 |  |  |  |
|  |  |  | 18-185 |  | 5 | 10240 | 5120 | 2560 |  |  |  |
| 8 |  | 4.5x10^13^ | 18-038 | Day 180 | < 5 | 320 | 640 | 640 | 1280 | 1280 | 1280 |
|  |  |  | 18-158 |  | 80 | 2560 | 5120 | 1280 | 1280 | 2560 | 2560 |
|  |  |  | 18-170 |  | 40 | 2560 | 1280 | 1280 | 2560 | 2560 | 640 |
