# Supplementary material for: Efficacy and Safety of a Krabbe Disease Gene Therapy
Source: Hum Gene Ther. 2022 May 16;33(9-10):499–517. doi: 10.1089/hum.2021.245 (PMC9142772; doi:10.1089/hum.2021.245)
Supplement: Supplemental data [file Supp_FigureS8.docx]

**
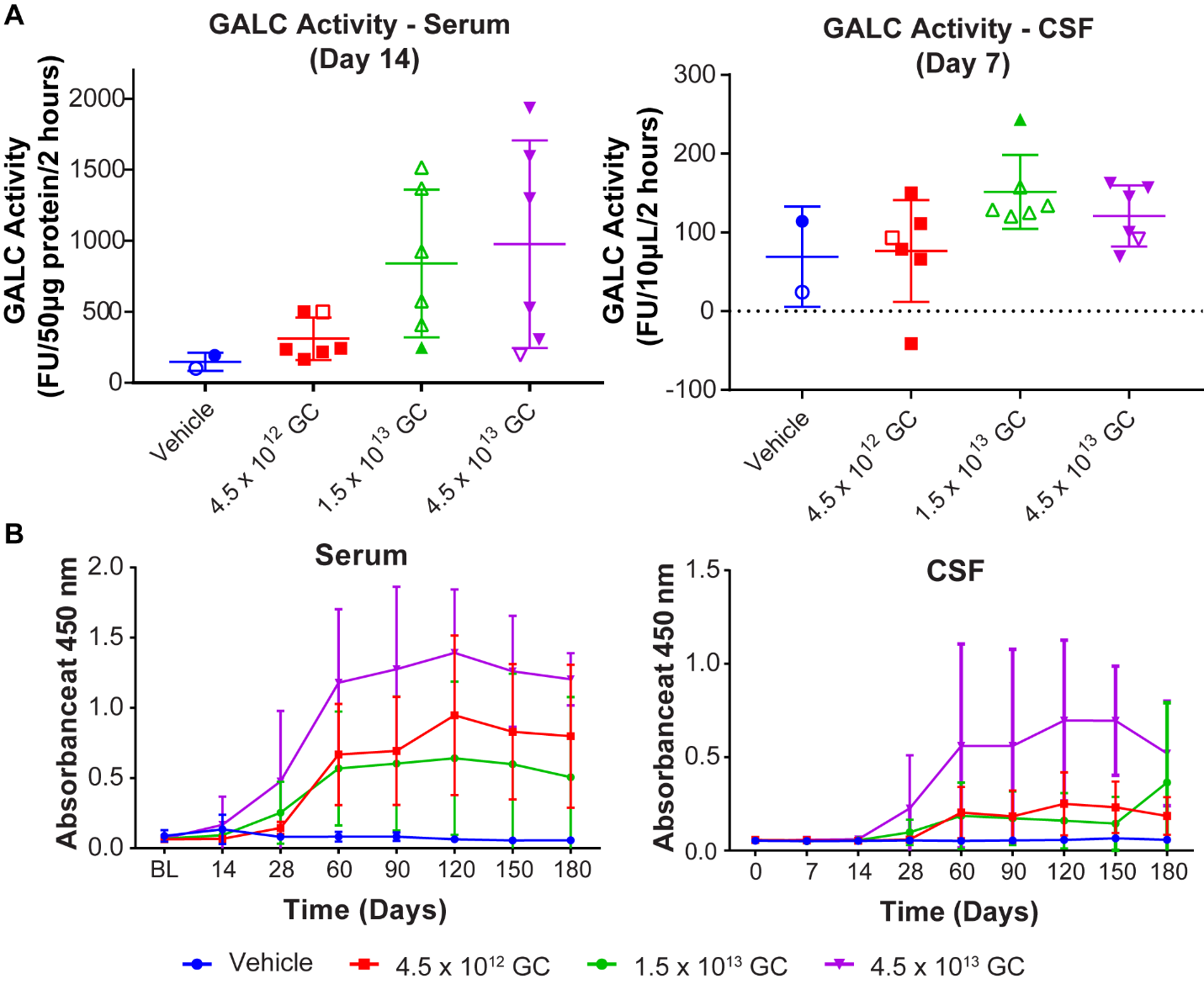
**

**Figure S8. GLP toxicology study in rhesus macaques, pharmacology, and immunology**

A. GALC enzyme activity 2 weeks post dosing in the serum and 1 week post dosing in CSF of juvenile rhesus macaques treated ICM with either 1 ml artificial CSF (vehicle, n = 2) or AAVhu68.CB7.hGALCco.rBG at one of three doses: 4.5 x 10^12^ GC (low dose, n=6), 1.5 x 10^13^ GC (mid dose, n-6), or 4.5 x 10^13^ (high dose, n = 6). Open symbols represent animals that had pre-existing neutralizing antibodies to AAVhu68. B. Anti-human GALC antibody ELISA in the serum and CSF of the same animals as in (A). Dilution 1:1,000 for serum and 1:20 for CSF.
