# Supplementary material for: Efficacy and Safety of a Krabbe Disease Gene Therapy
Source: Hum Gene Ther. 2022 May 16;33(9-10):499–517. doi: 10.1089/hum.2021.245 (PMC9142772; doi:10.1089/hum.2021.245)
Supplement: Supplemental data [file Suppl_TableS4.docx]

**Table S4.** ELISPOT responses to the capsid, NHP toxicology study

| **AAVhu68 peptide pools** | | **PBMC** | | | | | | | **Lymph node** | **Spleen** | **Bone Marrow** | **Liver** |
| --- | --- | --- | --- | --- | --- | --- | --- | --- | --- | --- | --- | --- |
|  |  | D0 | D28 | D60 | D90 | D120 | D150 | D180 | D90 | D90 | D90 | D90 |
| LD | 18-091 | **-** | **-** | **-** | **95** | **N/A** | **N/A** | **N/A** | **-** | **-** | **-** | **-** |
|  | 18-168 | **-** | **-** | **-** | **-** |  |  |  | **-** | **-** | **-** | **105** |
|  | 18-173 | **-** | **-** | **-** | **-** |  |  |  | **-** | **-** | **-** | **-** |
|  | 18-042 | **-** | **-** | **-** | **-** | **-** | **-** | **73** | **-** | **-** | **-** | **60** |
|  | 18-121 | **-** | **-** | **-** | **-** | **-** | **-** | **-** | **-** | **-** | **-** | **-** |
|  | 18-171 | **-** | **-** | **-** | **-** | **-** | **-** | **-** | **-** | **-** | **-** | **-** |
| MD | 18-167 | **-** | **-** | **-** | **-** | **N/A** | **N/A** | **N/A** | **-** | **-** | **-** | **-** |
|  | 18-176 | **-** | **-** | **-** | **-** |  |  |  | **-** | **-** | **-** | **-** |
|  | 18-187 | **-** | **-** | **-** | **-** |  |  |  | **-** | **-** | **-** | **-** |
|  | 18-055 | **-** | **-** | **-** | **-** | **-** | **-** | **138** | **-** | **-** | **-** | **-** |
|  | 18-181 | **-** | **-** | **-** | **-** | **-** | **-** | **-** | **-** | **-** | **-** | **-** |
|  | 18-183 | **-** | **-** | **-** | **-** | **-** | **-** | **-** | **-** | **-** | **-** | **-** |
| HD | 18-080 | **-** | **-** | **-** | **-** | **N/A** | **N/A** | **N/A** | **-** | **-** | **-** | **178** |
|  | 18-166 | **-** | **-** | **-** | **-** |  |  |  | **-** | **-** | **-** | **85** |
|  | 18-185 | **-** | **-** | **-** | **60** |  |  |  | **-** | **-** | **-** | **-** |
|  | 18-038 | **-** | **-** | **-** | **-** | **-** | **-** | **-** | **-** | **-** | **-** | **-** |
|  | 18-158 | **-** | **-** | **-** | **-** | **-** | **-** | **-** | **-** | **-** | **-** | **-** |
|  | 18-170 | **-** | **-** | **-** | **-** | **-** | **-** | **-** | **-** | **-** | **-** | **-** |
| ctrl | 18-162 | **-** | **-** | **-** | **-** | **-** | **-** | **-** | **-** | **-** | **-** | **-** |
|  | 18-159 | **-** | **-** | **-** | **-** | **N/A** | **N/A** | **N/A** | **-** | **-** | **-** | **-** |

Blue indicates a negative response; red indicates a positive response. For positive responses, the number indicates the average spot-forming units (SFU) per million cells from duplicate analysis. Three peptide pools were used to stimulate the cells. In cases when multiple pools generated a positive response, only the highest value was reported
